# A Stochastic Model Correctly Predicts Changes in Budding Yeast Cell Cycle Dynamics upon Periodic Expression of *CLN2*

**DOI:** 10.1371/journal.pone.0096726

**Published:** 2014-05-09

**Authors:** Cihan Oguz, Alida Palmisano, Teeraphan Laomettachit, Layne T. Watson, William T. Baumann, John J. Tyson

**Affiliations:** 1 Department of Biological Sciences, Virginia Tech, Blacksburg, Virginia, United States of America; 2 Department of Computer Science, Virginia Tech, Blacksburg, Virginia, United States of America; 3 Bioinformatics and Systems Biology Program, School of Bioresources and Technology, King Mongkut's University of Technology Thonburi, Bangkok, Thailand; 4 Department of Electrical and Computer Engineering, Virginia Tech, Blacksburg, Virginia, United States of America; 5 Department of Mathematics, Virginia Tech, Blacksburg, Virginia, United States of America; 6 Department of Aerospace and Ocean Engineering, Virginia Tech, Blacksburg, Virginia, United States of America; Fondazione Edmund Mach, Research and Innovation Centre, Italy

## Abstract

In this study, we focus on a recent stochastic budding yeast cell cycle model. First, we estimate the model parameters using extensive data sets: phenotypes of 110 genetic strains, single cell statistics of wild type and *cln3* strains. Optimization of stochastic model parameters is achieved by an automated algorithm we recently used for a deterministic cell cycle model. Next, in order to test the predictive ability of the stochastic model, we focus on a recent experimental study in which forced periodic expression of *CLN2* cyclin (driven by *MET3* promoter in *cln3* background) has been used to synchronize budding yeast cell colonies. We demonstrate that the model correctly predicts the experimentally observed synchronization levels and cell cycle statistics of mother and daughter cells under various experimental conditions (numerical data that is not enforced in parameter optimization), in addition to correctly predicting the qualitative changes in size control due to forced *CLN2* expression. Our model also generates a novel prediction: under frequent *CLN2* expression pulses, G1 phase duration is bimodal among small-born cells. These cells originate from daughters with extended budded periods due to size control during the budded period. This novel prediction and the experimental trends captured by the model illustrate the interplay between cell cycle dynamics, synchronization of cell colonies, and size control in budding yeast.

## Introduction

A major objective in systems biology is the development of predictive mathematical models. This allows researchers to test hypotheses and also guides future experimental studies. The combined use of mathematical models and experiments can impact real life applications, such as drug discovery, when the models can accurately predict the changes in the behavior of an organism under specific perturbations. The particular model structure used in a study is largely determined by the existing experimental data that needs to be incorporated into the model and the kinds of predictions one intends to make. Deterministic models are ideal for reproducing population averaged experimental observations, such as Western blot data. On the other hand, one resorts to stochastic models to describe noisy gene expression patterns and behaviors of heterogeneous cell populations.

For the last two decades, our research group has been interested in modeling the cell cycle of budding yeast. Experimentally, budding yeast is an ideal system for studying the cell cycle due to its rapid cell growth and proliferation, relatively small genome, and ease of genetic perturbations. In order to investigate how budding yeast cells respond to particular inputs and the regulatory mechanisms that shape these responses, one has to account for the cell cycle phase dependent nature of these mechanisms [1]. This approach requires synchronized cell populations [2]. In other words, cells need to be in the same state with respect to their cell cycle stage [3], size or other features so that the observed cell cycle progression would start from the same point among all cells in the population. However, under normal growth conditions, budding yeast cells are asynchronous. There are two widely used approaches to synchronize populations of yeast cells [4]. The first one is block and release that is used to force all cells within a population into synchrony, whereas the second method is centrifugal elutriation in which synchronous subpopulations within an asynchronous population of cells can be selected. In the block and release approach, an agent is used to uniformly arrest a cell population. Then, when this block is released, synchronized cells move into subsequent cell cycle phases, and samples can be collected at different time points. This efficient method has one significant drawback: agent specific effects separate from the cell cycle effects can be present, which can bias the experimental analysis and lead to wrong conclusions about the cell cycle's inner workings [2]. In the second method (centrifugal elutriation), cells from an asynchronous population are separated based on their density. The need for specialized expensive equipment and possible induction of stress responses are the disadvantages of this approach.

The synchronization approach that we will focus on here involves external perturbations to the budding yeast cell cycle control system to synchronize the activity of a key cell cycle protein among cells in colonies. Before we describe this approach in detail, we provide some background on the budding yeast cell cycle. Events required for cell cycle progression in budding yeast are controlled by cyclin-dependent kinases (CDKs) [5]. Cyclins regulate the cell cycle by controlling the activities of CDKs. By phosphorylating several target proteins, cyclin-CDK complexes drive the timely execution of cell cycle events [6]. Periodic changes in the levels of cyclins direct the events that lead to cell growth, DNA synthesis, and cell division. For instance, in order for the G1-S transition to occur, at least one of Cln1, Cln2, or Cln3 is needed. In wild type cells, Cln3-CDK complex accumulates as the cell gets bigger and when a certain cell size is reached, the synthesis of Cln1 and Cln2 is activated. Cln1 and Cln2 promote budding as well as the activation of Clb5 and Clb6, involved in the activation of replication origins, which is a key step for DNA synthesis. Clb1 and Clb2 drive mitosis and are responsible for events such as mitotic spindle formation. In other words, cyclin levels and the cell state (e.g., size, extent of budding/spindle formation/DNA synthesis) are highly correlated. Hence, it is natural to think of experiments to equalize levels (or activities) of cyclins among cells (by means of external perturbations) to obtain populations of cells that are synchronized with respect to their state. Recently, budding yeast cells have been successfully synchronized by periodic induction of *CLN2* (a G1 cyclin) expression using a *MET3-CLN2* [7]. The microfluidic device used in that study enabled tight control of *CLN2* expression from the *MET3* promoter by rapid changes in the methionine concentration (*MET3* off/on when methionine is present/absent in the media), while allowing imaging of the monolayer cell culture to measure the efficiency of synchronization under different experimental conditions and also to quantify the responses of mother and daughter cells in terms of cell cycle statistics. Furthermore, upon periodic pulsing of *CLN2* expression, significant changes in size control within different cell cycle phases have been observed. G1 phase size control refers to the dependence of G1 duration on the cell size at birth, whereas size control past G1 phase (S/G2/M) refers to the dependence of the budded period on cell size at budding [7].

In this paper, we use a stochastic model of the budding yeast cell cycle [8] that has been parameterized by fitting deterministic simulations to the observed phenotypes of 110 mutant strains of budding yeast [9] and by fitting stochastic simulations to statistical distributions of cell cycle properties in populations of yeast cells [7], [10]. Then, we assess the model's predictive ability by comparing experimental results on forced synchronization of cells [7] with model predictions. Along the way, we gain insights into changes in cell dynamics under external perturbations and we make a novel, testable prediction about cell synchronization in response to short-period pulses of cyclin expression.

## Methods

### A recent stochastic model of the budding yeast cell cycle

The budding yeast cell cycle model we use in this study is based on a recent model developed by Laomettachit [8]. This model contains three classes of variables. The first class of variables are modeled by mass action kinetics of transcription factor synthesis and proteolytic degradation, whereas the second class of variables are modeled by sigmoidal functions that describe the phosphorylation and dephosphorylation reactions. The third class of variables consist of protein complexes modeled by maximum or minimum functions based on the quasi steady state assumption due to the fast time scale of these complex formation processes. We recently modified this model by adding a more detailed spindle checkpoint mechanism using four new variables ([Mad2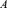], [UDNA], [SPNALIGN], and [ORIFLAG]) that are described in Table S1. This modification forces spindle checkpoint to be intact only during the time period from the onset of DNA synthesis (after the relicensing of origins of replication) until the time of spindle alignment at which point the checkpoint is lifted. Among the new variables, [UDNA] represents the state of DNA replication, [ORIFLAG] represents the state of replication origins relicensing, whereas [Mad2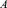] is the level of active Mad2 that sequesters Cdc20 to prevent premature mitotic exit until spindle alignment, which is represented by [SPNALIGN]. We refer the former model in [8] as “Model 1”, whereas the newer version is named as “Model 2”. Having a more detailed spindle checkpoint mechanism, Model 2 was found to be more robust compared to Model 1 in terms of maintaining the correct progression of cell cycle events against random parametric perturbations (results not shown). This provided us an advantage during the parameter optimization stage during which a global search is performed in the parameter space for model fitting with experimental single cell data.

For stochastic implementation of Model 2, Langevin approach is used to add molecular noise into the deterministic model equations. Conversion between the stochastic version of Model 2 (describing the time evolution of the numbers of molecules) and the deterministic version of Model 2 (describing the time evolution of the molecular concentrations) requires characteristic concentrations of each species in nM (listed in Table S2), typical budding yeast cell volume (28 fL), and Avogadro's number. Details of conversion can be found in [8], [11]. All simulation results reported in this study are generated by Model 2.

### Parameter optimization

Development of accurate and predictive mathematical models requires incorporation of experimental data into mathematical models. To this end, we first tuned the parameters of Model 2 in order to capture as many of the 119 experimental phenotypes observed with different genetic strains (wild type cells in glucose and galactose in addition to 117 mutants) in deterministic simulations. Of the 119 phenotypes that are listed in Table S3, Model 2 captured 110 phenotypes after this initial calibration of parameters. The remaining nine phenotypes that are not captured by Model 2 are listed in the Table S4 with the details of the mismatches between the model and experimental phenotypes. For viability, a specific order of cell cycle events is enforced as shown in Table S5. Our motivation behind integrating this extensive set of phenotypes into our model was to constrain the model parameters as previously demonstrated by the sensitivity analysis in [9]. We note that the parameters in the deterministic version of Model 2 are also used in stochastic simulations, whereas there is an additional group of parameters (listed in Table S2) that are exclusive to the stochastic version of Model 2. In order to estimate these additional parameters, we used the experimental single cell statistics obtained with the *CLN3* deletion (*cln3*) strain, since *cln3* is the background strain used in the experiments with forced *CLN2* expression pulses [7]. During this estimation process, we preserve the deterministic model's ability to capture 110 phenotypes by only varying the model parameters exclusive to the stochastic model. The descriptions and the values of the parameters that are present both in the stochastic and deterministic models are listed in Tables S6 and S7, respectively.

Moment closure based approaches are widely used for estimating stochastic model parameters [12], [13]. Due to their mathematical complexity, these approaches require simple models with relatively few parameters. An alternative path is to systematically explore the parameter space by a global optimization approach [14] while iteratively improving model's fit to experimentally measured statistics. We choose the latter approach due to the complexity of the budding yeast cell cycle model in this study. Starting from initial values, we optimize the parameters that are exclusive to the stochastic version of Model 2 using a combination of Latin hypercube (LH) sampling and differential evolution (DE). Previously, we used the same approach on a deterministic version of Model 1 as described in [9]. We first perform LH sampling within the parameter ranges listed in Table S2 to generate a group of parameter vectors (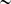
20 vectors as in [9]). Starting from this group, the fitting error (described in the caption of Table 1) is minimized using DE.

**Table 1:** Single cell statistics of *cln3* strain (mass doubling time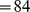 min): experimental and simulation values (before and after parameter optimization).

	Experiment [7]	Simulation before optimization	Simulation after optimization
Mean cycle time (M)	71.00 min	77.10 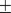 0.44 min	73.46 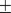 0.19 min
Mean cycle time (D)	94.00 min	87.18 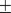 0.69 min	92.54 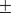 0.49 min
Mean 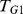 (M)	18.00 min	28.65 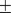 0.36 min	24.75 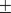 0.18 min
Mean 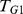 (D)	36.00 min	39.97 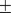 0.51 min	45.63 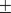 0.38 min
CV cycle time (M)	0.17	0.19 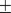 0.01	0.13 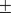 0.00
CV cycle time (D)	0.24	0.31 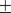 0.01	0.27 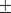 0.00
CV 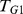 (M)	0.38	0.51 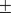 0.01	0.38 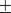 0.01
CV 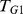 (D)	0.52	0.64 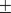 0.01	0.51 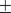 0.01
# complete cycles	-	2558 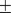 226	2573 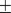 241
Cycle failure ratio	-	0.00 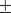 0.00	0.00 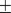 0.00
Fitting error	-	0.23 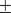 0.01	0.14 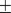 0.00

Parameter optimization results in 39% reduction in the fitting error. Fitting error is defined as 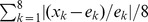, where 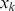 and 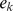 are the statistical data points in simulations and experiments, respectively (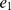: mean cycle time of mothers, 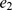: mean cycle time of daughters, 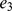: mean G1 duration of mothers, 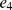: mean G1 duration of daughters, 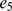: CV of cycle time among mothers, 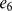: CV of cycle time among daughters, 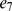: CV of G1 duration among mothers, 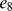: CV of G1 duration among daughters, and 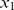 through 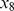 denote the simulation values for the same statistics). Simulation statistics (mean 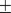 standard deviation) are computed from 15 independent realizations. In each realization, eight pedigrees are generated. Each pedigree of cells is initiated by a single daughter (D) or mother (M) cell. CV denotes coefficient of variation (standard deviation normalized by the mean), whereas 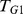 represents the G1 duration. Experimental mass doubling time of 84 minutes [7] is used in the simulations. The number of failed cycles (due to event execution errors listed in Table S9) normalized by the number of complete cycles is the cycle failure ratio. Matlab script to reproduce the mean and CV values (rightmost column) is provided as File A in File S1.

Among the *cln3* statistics, it is especially important for the stochastic model to capture average cycle times for mothers and daughters, since distinct period values of *CLN2* expression pulses in [7] were chosen based on these average cycle times. Other important statistics to capture are the coefficients of variation of G1 duration and cycle time among *cln3* mother and daughter cells. Strength of pulses of *CLN2* expression in the experiments are such that these coefficients of variation among daughter cells are reduced to the mother variability levels [7]. We note that this reduction among daughters is crucial for the synchronization of cell colonies.

Our starting parameter vector for the stochastic model is already in good agreement with the wild type single cell statistics reported in [10] before optimizing the parameters that are exclusive to the stochastic version of Model 2 with *cln3* statistics. Table 1 shows that after parameter optimization (six generations of DE or 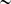
120 function evaluations), *cln3* statistics are captured much better by the model (39% reduction in the fitting error), while overall fitting error in terms of wild type statistics (not enforced during optimization) remain unchanged (Table 2). We also capture the abundances of key cell cycle proteins within threefold of experimental values [15], [16] as shown in Table S8. In addition, we note that the stochastic simulation statistics presented in Tables 1, 2, and S8 have coefficient of variation (CV) values of less than 10% (low variability) among 15 independent realizations.

**Table 2:** Single cell statistics of the wild type strain (mass doubling time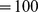 min): experimental and simulation values (before and after parameter optimization).

	Experiment [10]	Simulation before optimization	Simulation after optimization
Mean cycle time (M)	87.00 min	86.70 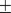 0.73 min	84.26 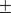 0.55 min
Mean cycle time (D)	112.00 min	111.95 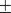 0.77 min	114.71 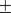 0.57 min
Mean 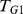 (M)	16.00 min	24.11 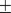 0.43 min	22.89 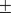 0.39 min
Mean 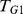 (D)	37.00 min	36.40 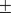 1.75 min	43.41 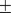 0.74 min
CV cycle time (M)	0.14	0.15 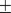 0.00	0.14 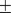 0.01
CV cycle time (D)	0.22	0.19 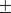 0.01	0.21 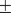 0.00
CV 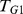 (M)	0.50	0.51 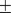 0.01	0.49 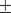 0.02
CV 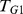 (D)	0.50	0.69 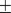 0.01	0.68 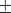 0.01
# complete cycles	-	1111 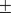 91	1098 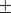 94
Cycle failure ratio	-	0.00 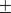 0.00	0.00 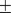 0.00
Fitting error	-	0.14 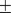 0.01	0.14 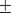 0.01

Parameter optimization using the experimental data in Table 1 does not affect the overall fitting error in terms of wild type statistics (these data are not enforced during optimization). Fitting error is defined as 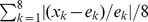, where 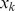 and 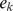 are the statistical data points in simulations and experiments, respectively (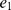: mean cycle time of mothers, 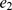: mean cycle time of daughters, 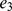: mean G1 duration of mothers, 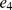: mean G1 duration of daughters, 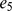: CV of cycle time among mothers, 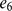: CV of cycle time among daughters, 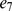: CV of G1 duration among mothers, 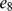: CV of G1 duration among daughters, and 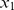 through 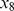 denote the simulation values for the same statistics). Simulation statistics (mean 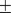 standard deviation) are computed from 15 independent realizations. In each realization, eight pedigrees are generated. Each pedigree of cells is initiated by a single daughter (D) or mother (M) cell. CV denotes coefficient of variation (standard deviation normalized by the mean), whereas 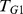 represents the G1 duration. Experimental mass doubling time of 100 minutes [10] is used in the simulations. The number of failed cycles (due to event execution errors listed in Table S9) normalized by the number of complete cycles is the cycle failure ratio. Matlab script to reproduce the mean and CV values (rightmost column) is provided as File B in File S1.

### Asynchrony among budding yeast cells

**Figure 1:**
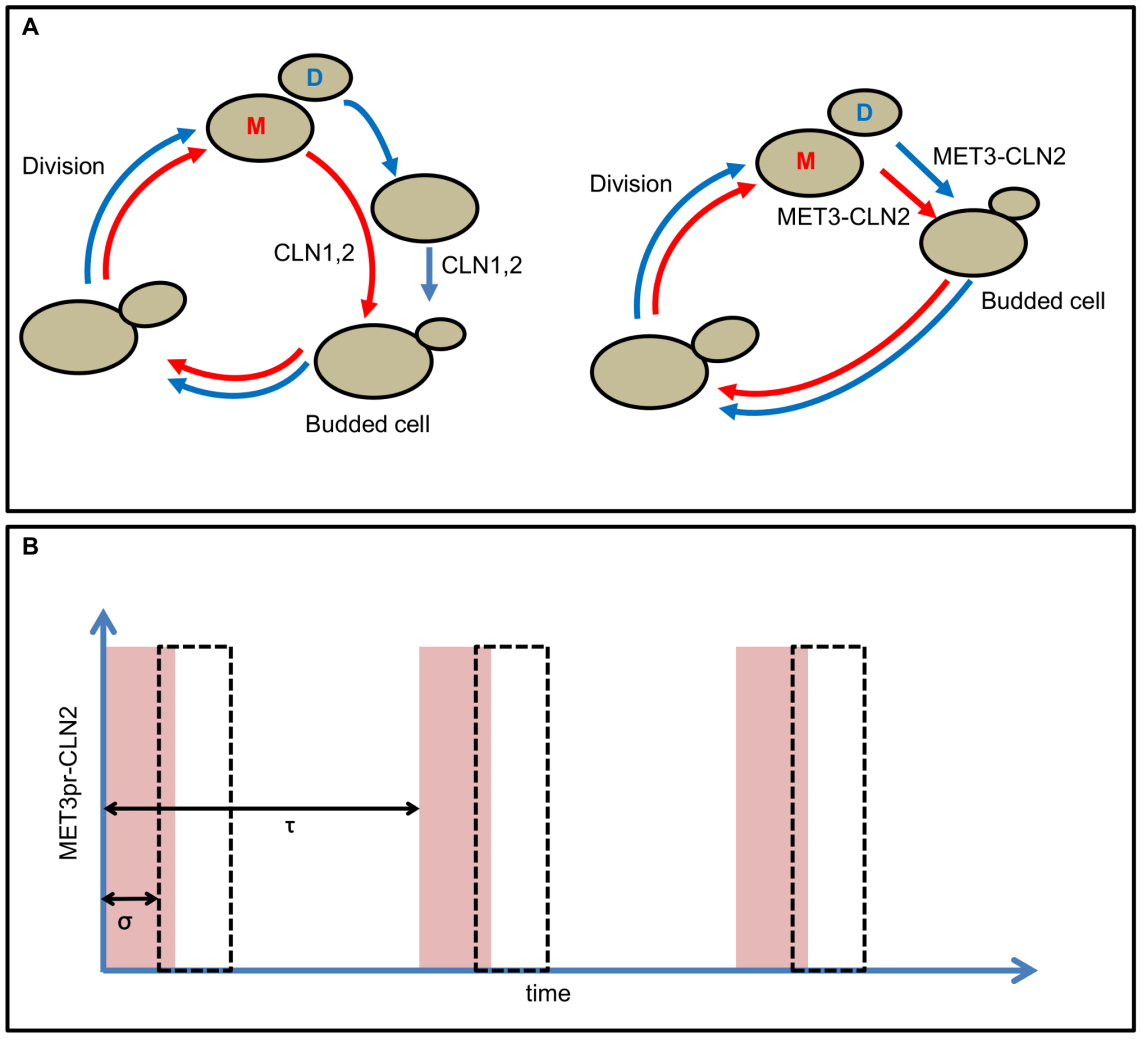
Synchronization of budding yeast cells. (A) Illustration of the forced *CLN2* expression experiment (reproduced from [7]). Normally, bud formation is driven by *CLN1* and *CLN2* expression in the budding yeast cell cycle. Daughter cells have longer unbudded periods since they are significantly smaller than the mother cells at birth. Forced periodic expression of *CLN2* from the *MET3* promoter forces mother and daughter cells to bud earlier compared to unforced conditions. (B) 20 min pulses of *CLN2* expression are driven by the periodic activation of the *MET3* promoter (period of 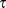). Pink shaded areas show the time intervals during which methionine is removed from the media. Dashed lines indicate the time periods during which the *MET3* promoter is actually active in the simulations, taking into account the 16.8 min lag time (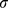) reported for this promoter in [17]. We assume the same lag time for *MET3* turn-on and turn-off.

A population of growing budding yeast cells is normally asynchronous. One reason for this asynchrony is the asymmetric division process of budding yeast cells, producing a large mother cell and a small daughter cell (Figure 1A). In our stochastic simulations, an average of 58% (with 2.9% standard deviation) of the size (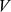) of a dividing cell goes to the “mother” cell at birth, whereas the remaining part is retained by the “daughter cell” as it returns to G1 phase. Consequently, on average, daughter cells have a longer interdivision time than mother cells (94 min vs. 71 min for *cln3* cells [7]). The observed difference in cycle times is due in large part to different times spent, on average, in G1 phase of the cell cycle. Another important factor causing asynchrony, even among mother (or daughter) populations, is the significant CV value of cycle time. Since the major source of this variability is the variable duration of G1 phase (CV of G1 duration is 0.38 for mothers and 0.52 for daughters among *cln3* cells [7]), externally forcing a population of cells to bud, regardless of their size at a given point of time, is an appealing strategy for synchronizing budding yeast cells. In fact, pulses of *CLN2* expression driven by the *MET3* promoter successfully synchronize budding yeast cells as reported in [7], [17].

### Simulations with pulses of *CLN2* expression

In order to test the predictive ability of our model against the experimental data in [7], we perform simulations with periodic *CLN2* expression conditions. In these simulations, we attempt to mimic the experimental conditions as well as possible. The induction of the *MET3* promoter (driving the periodic *CLN2* expression), which is synchronous among cells upon removal of methionine from the media, occurs with a delay of 16.8 min [17]. Both the synchrony of induction and the delay are taken into account in our simulations. Furthermore, we assume that upon addition of methionine into the media, MET3 shuts off completely (not gradually) with the same delay observed before turning on. For instance, in order to simulate expression from *MET3-CLN2* (with methionine removal from 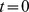 min to 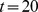 min), we turn on Cln2 synthesis from 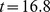 min to 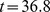 min (see Figure 1B). This is an approximation of the promoter dynamics characterized in [17]. Later, we will report results without this approximation and show that the model predictions are consistent even with a more complex promoter dynamics used in the simulations.

Next, we tune the strength of the *MET3* promoter (constitutive Cln2 synthesis rate from the *MET3-CLN2* construct) in the simulations. Among the three *MET3-CLN2* synthesis rates (low, medium, and high strengths listed in Table 3), the medium strength is chosen in the subsequent simulations for the following reasons.

**Table 3:** Single cell statistics of *cln3 MET3-CLN2* with periodic *CLN2* expression (period 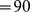 min): experimental and simulation values with three different *MET3pr* strengths.

	Experiment [7]	Low strength	Medium strength	High strength
*MET3pr* strength	-	0.5	1	4
Mean cycle time (M)	78.00 min	75.33 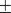 0.23 min	78.01 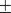 0.26 min	79.29 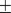 0.70 min
Mean cycle time (D)	89.00 min	89.45 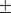 0.43 min	85.95 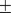 0.33 min	85.57 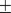 0.58 min
Mean 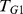 (M)	24.00 min	28.96 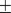 0.20 min	32.86 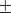 0.31 min	33.96 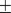 0.72 min
Mean 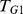 (D)	31.50 min	42.93 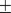 0.32 min	40.28 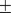 0.23 min	40.65 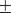 0.48 min
CV cycle time (M)	0.15	0.13 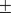 0.00	0.14 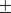 0.00	0.16 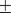 0.01
CV cycle time (D)	0.16	0.24 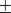 0.01	0.18 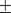 0.00	0.15 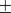 0.01
CV 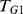 (M)	0.37	0.37 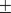 0.01	0.38 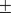 0.01	0.47 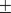 0.02
CV 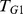 (D)	0.37	0.47 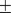 0.01	0.36 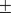 0.01	0.35 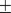 0.01
# complete cycles	-	2868 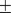 281	3369 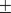 210	3025 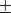 480
Cycle failure ratio	-	0.00 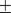 0.00	0.00 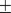 0.00	0.05 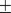 0.00
Fitting error	-	0.19 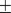 0.01	0.12 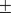 0.00	0.15 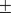 0.00

For each *MET3pr* strength value, the simulation statistics (mean 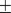 standard deviation) are computed from 15 independent realizations. In each realization, eight pedigrees are generated. Each pedigree of cells is initiated by a single daughter (D) or mother (M) cell. Fitting error is defined as 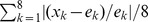, where 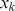 and 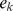 are the statistical data points in simulations and experiments, respectively (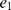: mean cycle time of mothers, 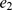: mean cycle time of daughters, 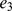: mean G1 duration of mothers, 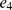: mean G1 duration of daughters, 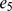: CV of cycle time among mothers, 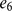: CV of cycle time among daughters, 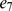: CV of G1 duration among mothers, 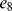: CV of G1 duration among daughters, and 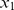 through 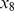 denote the simulation values for the same statistics). *MET3pr* (*MET3* promoter strength) is the transcription rate of *MET3-CLN2* divided by the transcription rate from the native *CLN2* copy. Since the native copy is regulated by SBF, we normalize the full transcription rate by the time-averaged SBF concentration (0.3) in the simulations with no forced *CLN2* expression (*cln3*). The experimental time window 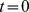 min to 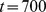 min is used in these simulations. As the results indicate, the medium strength *MET3pr* mimics experimental conditions the best among the three strengths (comparison between the strengths made in the main text). The number of failed cycles (due to event execution errors listed in Table S9) normalized by the number of complete cycles is the cycle failure ratio. Matlab script to reproduce the mean and CV values (medium *MET3pr* strength) is provided as File C in File S1.

- In low and medium strength *MET3-CLN2* simulations with a forcing period of 90 min, no cycles fail to complete due to an incorrect order of cycle events (correct order is enforced as described in Table S9). However, 5% of the cycles fail in the high strength *MET3-CLN2* simulations.
- The medium strength *MET3-CLN2* synthesis rate matches the native *CLN2* synthesis rate (second row of Table 3). This is in accord with the experimental characterization of the *MET3-CLN2* construct [7], [17].
- *cln3 MET3-CLN2* simulations (with medium *MET3-CLN2* strength) reproduce the single cell statistics in [7], namely the averages and CV values of cycle times and G1 durations among mothers and daughters with *CLN2* expression pulses reported in Figure S2B in [7] (forcing period of 90 min, forcing duration of 20 min per pulse), with significantly less fitting error compared to the low and high strength *MET3-CLN2* simulations (last row of Table 3). Using medium promoter strength in the simulations is the best choice here in order to avoid a mismatch with the experiments in terms of the effects of periodic forcing on cell cycle dynamics. All the results reported through the remainder of this paper are with medium *MET3-CLN2* strength unless otherwise stated.

In [7], the mass doubling time was measured as 84 min for the *cln3* background strain (no forcing) and it did not change significantly when various forcing periods ranging from 63 to 100 min were used to generate *CLN2* expression pulses. Therefore, we use the same mass doubling time (84 min) in all our simulations regardless of the presence of the pulse (or forcing) and the exact value of the forcing period.

Each *cln3 MET3-CLN2* simulation starts with a single cell. The initial condition set of this cell comes from the endpoint of a 2000 min (simulation clock) *cln3* simulation. For each *cln3 MET3-CLN2* simulation, a new initial condition set is generated from an independent *cln3* simulation. The same initial condition set that is used in all *cln3* simulations is given in Table S10. As each new cell is born, its trajectory (in terms of the numbers of molecules of all species) is followed until it gives birth to two cells (one mother and one daughter that are also followed). This process generates a pedigree of cells. Each pedigree simulation lasts for 700 min, which is also the total duration of the experiments in [7]. Stochastic differential equations (SDEs) of the model are solved by Euler's method with a fixed step size of 0.01 min.

### Monitoring the level of synchrony among budding yeast cells

In order to study the effects of periodic *CLN2* expression conditions, we monitor the evolution of the budding index (fraction of budded cells) with different periods of forced *CLN2* expression in our simulations. We use the experimental period values from [7]. To generate a budding index trajectory (from a single pedigree of cells), the budding index is recorded at 1000 equidistant time points between zero and 700 min during the simulations. In a perfectly synchronous population, the budding index would evolve as a periodic step function alternating between one (all cells budded) and zero (no cells budded) with a period equal to the period of forced *CLN2* expression. This makes the budding index a good measure of the degree of synchrony among a population of cells.

**Figure 2:**
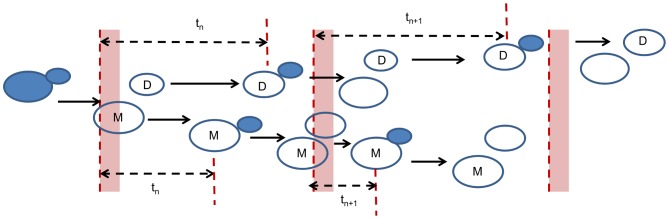
Generating return maps from the simulation data. Successive mother and daughter cells (denoted with “M” and “D”), are followed as illustrated here (reproduced from [7]). The elapsed time between the budding and the start point of the nearest preceding pulse in the current cycle is 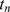, whereas the same elapsed time in the subsequent cycle is 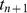. The same strategy was used to analyze the experimental data in [7].

In order to quantify the degree of synchrony among the mother cells and daughter cells separately, we generate return maps [7] from the simulation data. These maps are generated by following successive mothers and daughters and quantifying the time elapsed between the time of budding (end of G1) and the starting point of the nearest preceding pulse (illustrated in Figure 2). To achieve this, we extract subsequent mother and daughter cycles from each pedigree generated during the simulations. Synchrony among mothers (or daughters) with the pulses of cyclin expression would mean that the elapsed time between budding and start of the nearest preceding pulse is approximately equal in subsequent mother (or daughter) cycles. These subsequent elapsed time intervals are called 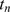 (for current cycle) and 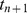 (for following cycle), respectively, as shown in Figure 2. Synchrony among cells would force data points (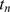, 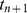) to lie close to the line 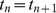 on the return maps. Moreover, if the cells are highly synchronized during the complete simulation time window (0–700 min), all successive mother (or daughter) pairs would be confined into a small region around the line 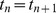 as long as the values of 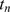 and 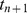 have very low variation. Color coding on the return maps is used to quantify the fraction of data points (data density) within map regions. For each return map, elapsed time intervals between budding and the start point of the nearest preceding pulse are extracted from the aggregation of eight independently generated pedigrees. The data is binned, which results in one data density value per bin (map region). Starting points of the pulses with different periods are listed in Table S11. While generating the return maps, we do not take into account the lag of promoter turn on/off upon methionine concentration changes in the media. This choice is consistent with the experimental return maps in [7]. Each return map has 400 regions: Horizontal (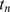) and vertical (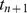) axes are divided equally into 20 subintervals, while the range of both axes is 0–90 min (4.5 min per subinterval). The statistics of the degree of synchrony are computed from independently generated 15 return maps (or 120 cell pedigrees) per forcing period.

### Quantifying the changes in G1 and S/G2/M size control due to *CLN2* expression pulses

Budding yeast cells are known to grow exponentially according to experimental measurements [18]. Exponential growth is also implemented in our model. As a result, cell size at budding (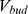) is related to the size at birth (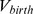) through 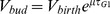 [10], where 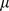 is the rate of exponential cell growth and 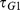 is the length of the G1 period during which the cells are unbudded. Hence, 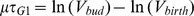. We define 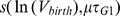 as the slope of the best least squares linear fit from the simulation data, where the vertical and horizontal axes are 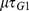 and 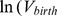), respectively. If the G1 duration is completely independent of the cell size at birth (no G1 size control), 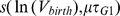 has a value around zero. As the strength of G1 size control increases, 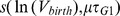 becomes more negative. Hence, we define G1 size control strength as 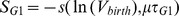. Similarly, the strength of the budded period size control (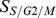) can be computed as the negative of the slope of 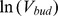 against 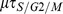. This slope is denoted by 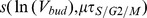, where 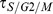 is the budded period duration.

The change in G1 size control upon forced *CLN2* expression is computed as
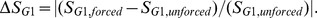
Here, “forced” and “unforced” size control strengths correspond to *cln3 MET3-CLN2* and *cln3* strains, respectively. Likewise, the same change in budded period size control is computed as
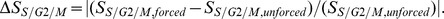



## Results and Discussion

### Varying the period of *CLN2* expression pulses

**Figure 3:**
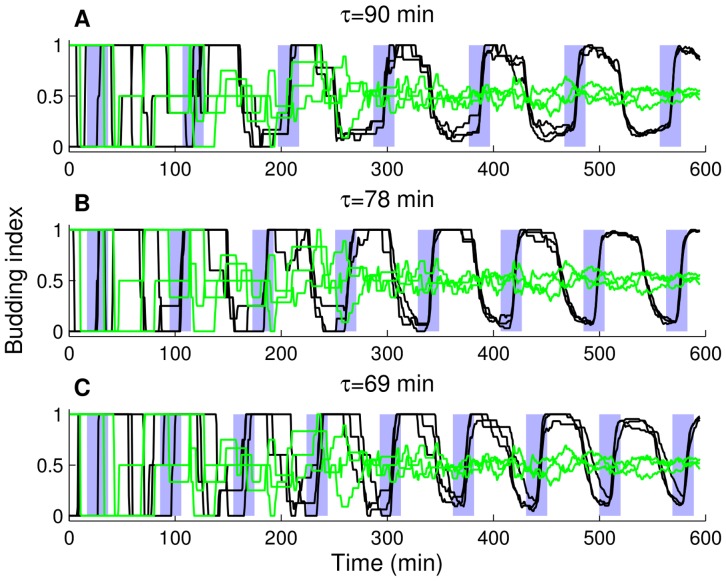
Budding index trajectories under different conditions. Simulation results. Evolution of the budding index (fraction of budded cells in a cell population at a given time) for the unforced cells (*cln3*, green lines) and the cells with forced *CLN2* expression (*cln3 MET3-CLN2*, black lines). Forcing period is 90 min in (A), 78 min in (B), and 69 min in (C). Each individual trajectory represents a pedigree initiated by a single daughter cell, three trajectories are shown per forcing period. Blue shaded areas represent the time intervals in which *MET3-CLN2* is active (time lag for the MET3 promoter turn on/off is taken into account).

Figure 3B shows that 78 min period pulses result in higher synchrony (more “step-function” like budding index trajectories) than 90 and 69 min period pulses (Figures 3A and 3C), whereas without any pulse (*cln3*) cell populations lack synchrony: the budding index settles around 0.5 (half of the population budded) after about 300 min. Later, we will quantitatively show that 78 min is the optimal period for synchronizing budding yeast cells among these three period values. Intuitively, this can be explained in terms of the observed mother and daughter natural cycle times without forced *CLN2* expression: pulses with a period of 69 min come much faster than the natural cycle time of daughter cells (94 min), and pulses with a period of 90 min come much slower than the natural cycle time of mother cells (71 min). The 78 min pulse period is midway between these cycle times, leading to good overall synchrony within the population compared to 90 and 69 min periods. We note that Figure 3B (evolution of budding index with 78 min pulse period) is in good qualitative agreement with Figure 1D in [7].

When *CLN3* is not deleted (for the *MET3-CLN2* strain), 78 min period pulses result in low synchrony (Figure S1). This is due to *CLN2* activation by *CLN3*, which eliminates the need for *CLN2* expression pulses to trigger budding. As a result, after the first cycle, the budding index does not reach values where nearly all cells are unbudded. This behavior was also observed experimentally (right column of Figure S1 in [7]).

Next, we quantify the degree of synchrony in our simulations using 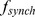, which is defined as the fraction of time points after 300 min (point of cell colony formation) at which the budding index is greater than 0.95 or less than 0.05. According to the statistical characterization of 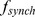 with the three different forcing periods in Table 4, the mean value of 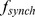 with 78 min forcing period is 92% higher than the mean 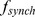 values with 69 and 90 min forcing periods (mean values computed from 15 independent realizations per forcing period). In addition, the variability of 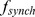 with 69 min period (most frequent pulses) is the highest (about 75% of the mean) due to noisy budding index trajectories, whereas the variability is about 35% of the mean with 90 min period, and 25% of the mean with 78 min period. Based on these statistical results, the 78 min forcing period is optimal, whereas 69 and 90 min forcing periods are equivalent in terms of 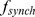, which measures the overall synchrony of a pedigree of cells based on the budding index trajectories. Also, the variability of 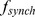 increases as the forcing period is moved away from the 78 min forcing period, which also supports the optimality of this forcing period for synchronizing cells.

**Table 4:** Synchronization levels with different forcing periods.

Forcing period (min)	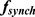
No forced *CLN2* expression	0.00 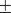 0.00 (0.00 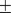 0.00)
90	0.13 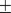 0.05 (0.13 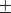 0.04)
78	0.25 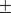 0.06 (0.23 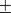 0.08)
69	0.13 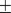 0.10 (0.14 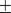 0.11)

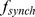
 is the fraction of time points (between 300 min and 700 min) at which more than 95% or less than 5% of the cells are budded during 700 min simulations. The simulation statistics (mean 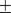 standard deviation) of 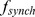 are computed from 15 independent realizations per forcing period. In each realization, a budding index trajectory is generated from a single pedigree of cells. Each pedigree starts from a single cell and the number of cells within the pedigree increases exponentially due to cell division. Values that are in parentheses are from the pedigrees that are initiated by single mother cells, whereas the remaining 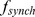 values are from the pedigrees that start with daughter cells. Results are consistent with these different initial condition choices.

On the budding index trajectories in Figure 3, the budding index value is initially one or zero (depending on the randomized initial condition) since we start each simulation with a single cell and 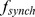 remains around one or zero until a cell colony is formed. After about 300 min (about 4 cycles), the colony has about 16 cells. On the *cln3* budding index trajectories in Figure 3, after 300 min, the budding index settles around 0.5 and fluctuates mildly around 0.5 until the end of the simulations. As shown in Table 4, the mean value of 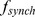 among 15 independent realizations is zero due to complete lack of synchrony among fifteen independently generated *cln3* cell pedigrees, whereas the mean value of 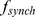 is at least 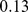 in *cln3 MET3-CLN2* simulations with 90, 78, and 69 min pulse periods.

### Return maps

As shown in Table 3, with 90 min period pulses, mother cell cycles (average duration of 78 min) slow down and daughter cell cycles (average duration of 89 min) speed up compared to the natural cycles (average durations of 71 and 94 min for mothers and daughters, respectively) [7]. In other words, forced *CLN2* expression pulls mother and daughter cycle times towards each other leading to a high degree of overall synchrony.

Let's first consider how the lack of synchrony can be visualized on the return maps. For this purpose, we use control return maps [7] that are generated by quantifying 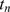 and 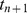 (depicted in Figure 2) from *cln3* simulation pedigrees without forced *CLN2* expression, but only by using the pulse start points in Table S11. The lack of synchrony can be observed in four different ways depending on the period of these start points and the level of variability of G1 duration and cycle time.

- If the natural cycle time (in the absence of periodic *CLN2* expression) is significantly shorter than the period that is used to make the return map, cells will move through the cycle faster than this period. Hence, the time elapsed between budding and the start point of the nearest preceding pulse will get shorter from one cycle to the next (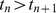) resulting in higher data density below the line 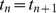 compared to the rest of the return map. For example, Figure S2D (73 min average mother simulation cycle time and 90 min period) shows a density plot where mothers are accumulated quite uniformly under the line 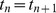. Cycles of mother cells are so much faster than the 90 min period that some of them bud twice within some of the individual 90 min subintervals. This causes the presence of some mothers at the upper left corner in Figure S2D.
- If the period that is used to make the return map is shorter than the natural cycle time, budding to start point of nearest preceding pulse duration lengthens from one cycle to the next (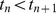). In this case, the return map has higher data density above the line 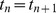 as shown in Figure S2F (73 min average mother simulation cycle time and 60 min period). Occasionally, when 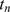 has a value around the pulse period, some mother cells may need two more pulses before the subsequent budding. In this case, subsequent budding may take place right after the second of these pulses (much smaller 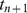 compared to 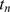). The small cluster (Figure S2F) below the line 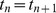 (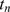/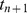 around 60 min/10 min) illustrates such mother cells.
- If the period that is used to make the return map is approximately equal to the natural cycle time, the return map will be dominated by data points along the line 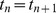 as shown in Figure S2E (73 min average mother simulation cycle time and 69 min period). These asynchronous mothers are not confined to a small region on the map, but rather cover the whole diagonal.
- Daughter control maps (with no forced expression) have rough and low density features (Figures S2A, S2B, and S2C). Regardless of the exact pulse period, these daughter maps do not exhibit any of the three cases described above due to the much higher variability of cycle time and G1 compared to mothers (Table 1). This qualitative observation was also made in [7] (Figure 2C).

### Extent of locking under different forcing periods

**Figure 4:**
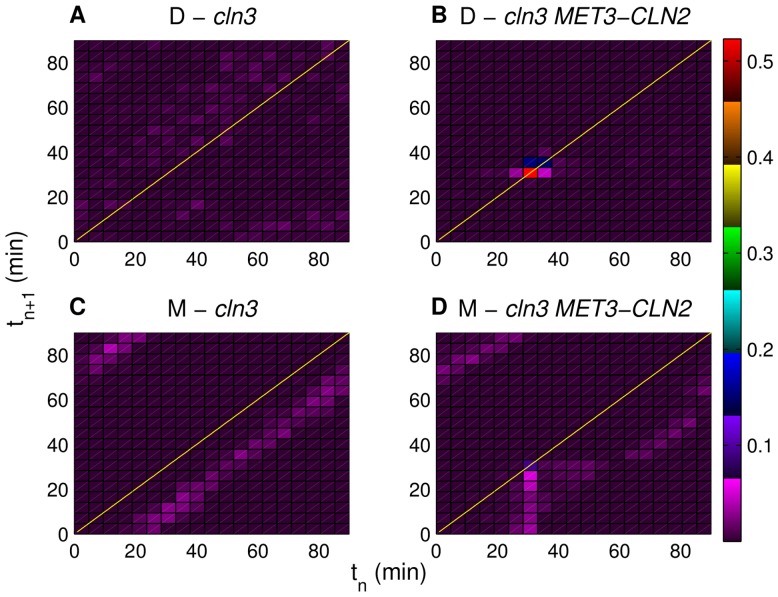
Simulated return maps for successive cells with and without forced *CLN2* expression. No forcing is applied in (A) and (C), whereas the forcing period is 90 min in (B) and (D). “D” and “M” stand for daughters and mothers, respectively. Each map divides the 0–90 min time interval into 20 equally sized subintervals. Colors represent the fraction of data points in each map region as depicted in the color map on the right. Only the bright colors of this map are used in the return maps except for the map regions with very low data density. Lines 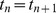 are depicted in yellow. Each return map is made using the data collected from eight independently generated pedigrees.

Figure 4 compares the return maps (with period of 90 min) generated with forced *CLN2* expression (*cln3 MET3-CLN2*) against the control maps (*cln3*). According to Figure 4B, half of the daughters (red map region with 0.5 data density) are on the diagonal at 30 min. These daughter cells are so called “locked cells” [7] since they are synchronized with the pulses of *CLN2* expression. Similar to Figure 2C in [7] (bottom left map), about 300 data points are visualized on this map. On the other hand, the return map for successive mothers with 90 min of forced *CLN2* expression (Figure 4D) show that only about 10% of the mothers (map region with 0.1 data density) are on the diagonal, whereas the majority are below the diagonal since the mother cycles are significantly faster than the incoming pulses (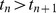). Figures 4A and 4C show the corresponding daughter and mother control maps, respectively. Both have low density map regions in striking contrast with the higher density map regions in Figures 4B and 4D. In the control maps, daughters are spread over the whole map, whereas mothers are mostly below the diagonal. Simulation results shown in Figure 4 qualitatively agree with Figure 2C in [7] that illustrates the experimental results (*cln3* versus *cln3 MET3-CLN2* maps) under the same conditions.

**Figure 5:**
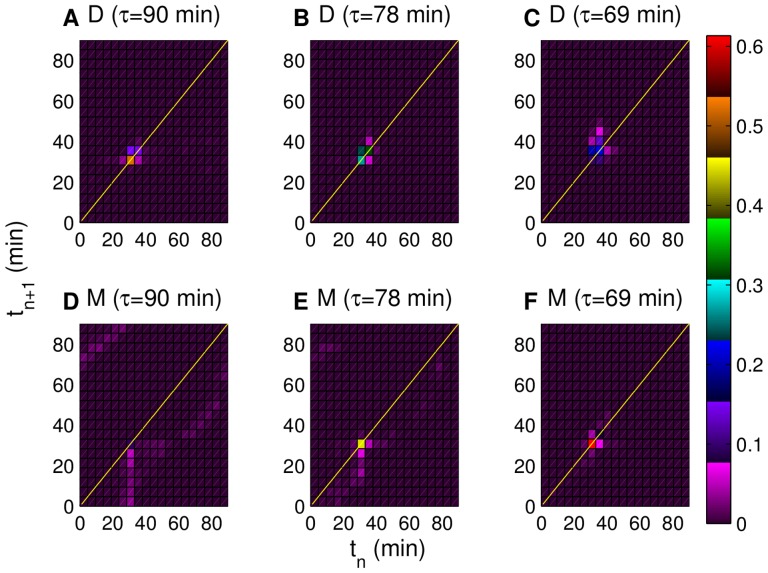
Simulated return maps for successive mother and daughter cells. Forced *CLN2* expression periods are 90 min in (A) and (D), 78 min in (B) and (E) and 69 min in (C) and (F). “D” and “M” stand for daughters and mothers, respectively. As the forcing period approaches the mother/daughter natural cycle time (71/94 min) the maximum data density on the mother/daughter return map increases. Colors represent the fraction of data points in each map region as depicted in the color map on the right. Only the bright colors of this map are used in the return maps except for the map regions with very low data density. Lines 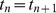 are depicted in yellow. Each return map is made using the data collected from eight independently generated pedigrees.

Next, we look at the effects of the pulse period variation in *cln3 MET3-CLN2* simulations. One way to quantitatively assess this is to compare the maximum data density values on the lines 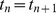 of the return maps with different pulse periods (90,78, and 69 min). According to Figure 5, for the mothers, the maximum density value is about 0.6 with pulse period of 69 min (Figure 5F), and 0.1 with pulse period of 90 min (Figure 5D). For daughters, the maximum data density is about 0.5 with pulse period of 90 min (Figure 5A), and 0.2 with pulse period of 69 min (Figure 5C). These results indicate that when the pulse period is significantly different than the natural cycle time, the maximum data density along the diagonal drops significantly. In other words, as the pulse period gets closer to the natural cycle time (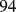 and 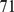 min for daughters and mothers, respectively) the extent of locking increases, which is the primary reason that the medium pulse period (78 min return maps in Figures 5B and 5E) is the optimal period for synchronizing the cell population as a whole.

To demonstrate this quantitatively, we specify a square (locking time window) around the line 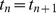 on each return map and compute the fractions of mothers and daughters that fall within the square (also done in [7]) as the extent of locking. Our goal is to compare the extent of locking in the simulations to the experimental values. We first determine our model's locking time window as described in Text S1 and Figure S3. Next, using this time window, we compute the fractions of locked mothers and daughters with different periods of forced *CLN2* expression. Summing the locked fractions of mothers and daughters with six different pulse periods (Table 5), we see that the 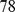 min pulse period is optimal for overall locking in the cell population according to both the model and the experiments in [7] (highest sum of locked fractions in Table 5). Among the six forcing periods, the sum of locked fractions among 15 independent realizations (eight pedigrees per realization) have low variability. The highest variability (standard deviation is about 5% of the mean) is with 63 min period (most frequent pulses).

**Table 5:** Sum of the fractions of locked mother and daughter cells at different forcing periods.

Forcing period (min)	Experiment [7]	Model
63	0.92	0.98 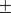 0.05
69	1.33	1.40 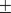 0.04
78	1.68	1.47 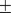 0.03
84	1.41	1.28 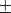 0.04
90	1.35	1.06 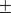 0.03
100	0.53	0.85 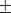 0.03

The simulation statistics (mean 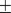 standard deviation) of the sums are computed from 15 independent realizations (return maps) per forcing period. In each realization, eight independent pedigrees are generated. Half of these pedigrees start from individual mother cells, whereas the remaining half start from individual daughter cells. The number of cells within each pedigree increases exponentially due to cell division. Locked cells in simulations occupy the optimal locking regime (red square in Figure S3) on the return maps with different forcing periods.

**Figure 6:**
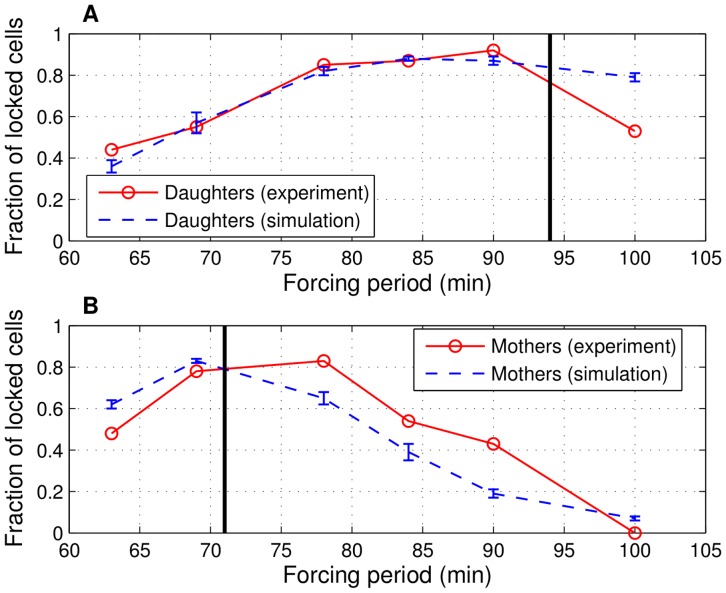
Fractions of locked daughters and mothers. Forced *CLN2* expression with six forcing periods: experimental [7] and simulation values for daughters in (A) and mothers in (B). Black vertical lines represent the natural (*cln3*, no forced *CLN2* expression) mother and daughter cycle times. The range of each locked fraction in the simulations (mean 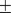 standard deviation) is depicted by the blue error bars, whereas the red circles correspond to experimental values. Each range from the simulations is computed from 15 independent realizations. Each realization contains eight independently generated pedigrees of cells generated over the course of 700 min starting from a single daughter or mother cell.

The evolutions of the fractions of locked daughters and mothers with six different pulse periods ranging from 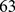 to 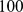 min are shown in Figures 6A and 6B, respectively. Mothers and daughters exhibit higher degrees of locking as the pulse period approaches their natural cycle times (marked with black vertical lines in Figures 6A and 6B). With 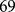 min period pulses, the majority of the mothers are locked (Figure 6B), whereas about 40% of the daughters are outside the locking regime (Figure 6A). Similarly, with 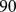 min period pulses, the majority of the daughters are locked, whereas the majority of the mothers are outside the locking regime. The maximum CV among 15 independently generated return maps is for the fraction of locked mothers (14%) with 100 min forcing period, whereas nine of the remaining twelve fractions have CV values of less than 10% (low variability). Hence, the stochastic model simulation results are consistent among different realizations. For the locked fractions, Pearson's correlation coefficient of the experimental values and the mean simulation values is found to be 0.87. This indicates that the model is successful in terms of capturing the trends in the evolution of the locked fraction of cells with respect to forcing period.

Among the total of six locked fraction values for daughters, the model correctly predicts four values within 5% of the experimental measurements, whereas the remaining values are predicted within 18% (forcing period of 63 min) and 49% (forcing period of 100 min) of the experimentally measured values. For mothers, four of the locked fractions are predicted within 29% of the experimental measurements, one value is predicted within 56% (90 min forcing period), whereas the remaining prediction is 0.07 (100 min forcing period) against the zero value measured in the experiments. Overall, the model predictions follow the experiments closely as nine of the twelve predictions have less than 30% error compared to the experimental values of locked fractions. We would like to note that the simulations with more complex promoter dynamics described in Text S2 (gradual turn-on and turn-off of the *MET3* promoter depicted in Figure S4) produce similar results as shown in Figure S5.

A significant mismatch between the model predictions and the experimental data in [7] is for the trend followed by the fraction of locked mothers as the forcing period changed from 69 min to 78 min (Figure 6B). When the forcing period is increased from 69 min to 78 min, the model predicts a significant drop in locked fraction of mothers (0.83 to 0.65), whereas the two fractions of locked mothers are approximately the same in the experiments as shown in Figure 6B. Before analyzing this outcome, we note that cells that are cycling faster than the pulses have reduced pulse to budding duration from one cycle to the next. Hence, 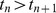 holds for fast cells and they appear below the diagonal (represented by 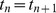) on the return maps. Conversely, slow cells appear above the diagonal. Experimental results (Figure 3B in [7]) indicate that the fast mothers running ahead of the pulses with 78 min period compensate for the slow mothers running behind the pulses with 69 min period. As a result, the fractions of locked (neither fast nor slow) mother cells with these two forcing periods are approximately equal. However, in our simulations, diminishing fraction of fast mothers as the forcing period changes from 78 min (Figure 5E: several fast mothers below the diagonal) to 69 min (Figure 5F: nearly no fast mothers below the diagonal) is not compensated by any significant increase (from Figure 5E to Figure 5F) in the extent of slow mothers above the diagonal with this change in the forcing period. It is the lack of such compensation that causes the model to predict significantly higher fraction of locked mothers with the 69 min forcing period (0.83) compared to the 78 min forcing period (0.65) despite the approximately equal fractions of locked mothers in the experiments. One potential reason for this discrepancy is the budded period size control that is shaped by a strength (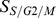 defined in Methods) and a size threshold (cell size at budding) below which the size control is active. It is possible that the model and the actual biological system have different values of these size control parameters for which we do not have experimental data. Cells that are affected by the budded period size control have longer budded periods compared to the remaining cells. The length of the budded period affects the cell state at division, which determines the states of the offspring cells (mother and daughter) at birth. The cell state at birth (i.e., size, concentrations of cyclins) is a major factor in terms of deciding how quickly the newborn cells are going to respond (bud formation) to incoming *CLN2* expression pulses. In fact, locked mothers with 78 min forcing period in [7], were found to be first generation mothers (with smaller birth size than the subsequently born mothers), which are born from daughter cells that were forced to bud by the *CLN2* expression pulses. Having long budded periods due to the budded period size control, these first generation mothers could not produce subsequent mothers that run ahead of the pulses. As a result, early generation mothers appeared as locked cells in the experiments [7]. The strength of the budded period size control and the size threshold below which this type of size control acts are very critical in terms of shaping the birth state of the cells born in subsequent generations. This makes the budded period size control (potentially different strengths and size thresholds in model and experiments) a likely reason for the absence of a locked state among mother cells in the simulations with 78 min forcing period despite the experimental locking range of 69–78 min.

### Single cell size trajectories under different forcing periods

If the cells were perfectly synchronized with the *CLN2* expression pulses, we would observe one budding event per pulse (between two consecutive pulses) [7]. In the absence of such synchronization, cells that cycle faster than the pulses would occasionally bud twice between consecutive pulses, whereas slow cells may sometimes skip a pulse (no budding between consecutive pulses) [7]. Next, we will look at the level of alignment between the timings of budding events and forced *MET3-CLN2* expression pulses under different forcing periods. One way to achieve this is to follow individual trajectories of cell size for mothers and daughters separately and compare these trajectories with the pulse intervals, while also keeping track of the budded and unbudded time intervals during individual cycles.

Cell size measurements in [7] are based on quantification of the pixel areas of cell profiles from an automatic cell segmenter. Before we start analyzing the simulation results in terms of cell size dynamics, we introduce a conversion scheme to ensure that the numerical range of cell size in the simulations aligns with the corresponding experiments. Figure S6 illustrates this conversion: values of average cell sizes in simulations (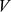) and cell areas in experiments (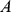) at birth/budding for mothers and daughters with/without 90 min period *MET3-CLN2* expression pulses (six data points in total) are plotted against each other. This is followed by the extraction of the best linear least squares fit (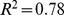). Cell size values generated in the simulations are converted to cell area values by using this best linear fit before comparing simulation results with experimental data.

**Figure 7:**
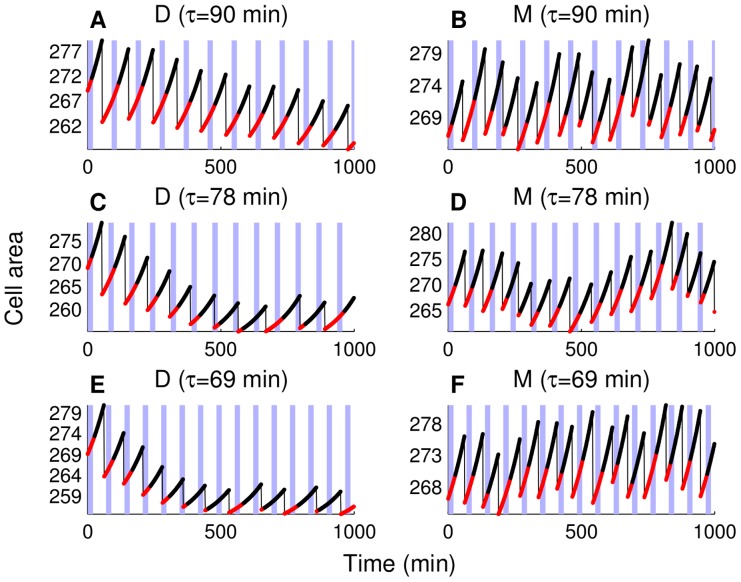
Cell size trajectories for successive mother and daughter cells. Data is collected during the simulations with three different periods of forced *CLN2* expression: 90 min (A) and (B), 78 min (C) and (D), and 69 min (E) and (F). Shaded blue areas show the time intervals with forced expression (without delay in the *MET3* turn-on/turn-off as in Figure 3C of [7]). Unbudded parts of the trajectories are plotted with red, budded parts are black, and thin black lines represent division events. The correct order of cell cycle events is enforced during the simulations (Table S9). Daughter simulations (marked with “D”) start from a daughter initial condition set, whereas mother simulations (marked with “M”) start from a mother initial condition set. These initial condition sets are extracted from the endpoints of 2000 min simulations with no forced *CLN2* expression.

Figure 7 shows the cell size trajectories for mothers and daughters with pulses of 90, 78, and 69 min periods. In these simulations, no pedigrees are generated. Instead, we follow a single mother or daughter cell after each division. The mother initial conditions are from the end points of 2000 min *cln3* mother simulations during which the fraction of cell mass retained after each division is a random number with a mean value of 0.58 and a standard deviation value of 0.029 (5% of the mean). Conversely, the daughter initial conditions come from the end points of *cln3* simulations during which the fraction of cell mass retained after each division is the remainder of the total mass after the mother fraction is assigned. As seen in Figure 7A, budding events in daughter cycles are perfectly synchronized with 90 min period pulses, whereas the budding events show the same behavior in mother cycles (Figure 7F) when the pulse period is 69 min period (one budding event per pulse in both cases). On the other hand, daughters skip pulses three times with fast pulses (69 min forcing period in Figure 7E), whereas mothers exhibit multiple budding events four times with slow pulses (90 min forcing period in Figure 7B) in 1000 min simulations. The 78 min forcing period (Figures 7C and 7D) is a good compromise between the slow daughters and fast mothers that tend to stay behind or run ahead of the pulses. The results in Figures 7A–7F agree qualitatively with Figure 3C in [7].

It is also interesting to note that (with 78 min forcing period) in the last 300 minutes of the 1000 minute simulations, daughters add two pulse skipping events (Figure 7C), whereas mothers add one multiple budding event (Figure 7D). This results in a threefold increase (one to four) in the total number of such events that are out of synchrony with the pulses. According to Table 6, until 700 min, mothers exhibit only one multiple budding event, whereas daughters never skip a pulse. This justifies the choice of the 0–700 min experimental time window used in [7], which results in a high degree of synchronization; a 50% increase in the length of this time window is predicted to produce a significant increase in the number of events showing asynchrony. Daughter cells need to become small enough to invoke budded period size control resulting in pulse skipping and it takes several cycles for the birth size to reach such a small value. Cell size keeps decreasing with each division as shown in Figure 7 (also observed in [7]). However, once the size control is invoked, small newborn cells (compared to the cells born in the first few cycles) will be more likely to skip pulses that will result in asynchrony among the cells in the population. Mothers, which cycle faster than the 78 min pulses, have smaller 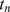 values (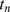 is depicted in Figure 2) as the time progresses and this results in some mothers budding twice between subsequent pulses.

**Table 6:** Analysis of the trajectories shown in Figure 7.

	# Pulse skipping	# Multiple budding	Total # cycles
D (90 min period)	0 (0)	-	11 (8)
M (90 min period)	-	4(2)	15 (10)
D (78 min period)	2 (0)	-	10 (8)
M (78 min period)	-	2(1)	15 (10)
D (69 min period)	3 (1)	-	11 (8)
M (69 min period)	-	0 (0)	14 (10)
D (no forcing)	-	-	10 (7)
M (no forcing)	-	-	15 (10)

Shown is the number of budding events that show lack of synchrony with the pulse for mother (M) or daughter (D) cells in 1000 min simulations. The number of observations until 700 min is shown in parentheses. Pulse skipping happens when the cell does not bud between two subsequent pulses, whereas observing multiple budding events between subsequent pulses is a consequence of the natural cycle time (with no forced *CLN2* expression) being significantly shorter than the forcing period. The total number of cycles without forced *CLN2* expression (*cln3*) is given in the last two rows for daughters and mothers, respectively. The numbers of pulse skipping events, multiple budding events, and total number of cycles are computed from a single mother or daughter trajectory per forcing period.

Finally, as shown in Table 6, we observe that the total number of cycles of mothers and daughters remains approximately the same regardless of the forcing period (also with no forcing): 10–11 daughter cycles and 14–15 mother cycles. This is another experimental trend captured by the model: in [7], the authors report that pulse skipping and multiple budding events are compensated for by the external perturbations (pulses of gene expression) resulting in a balance between the mass doubling time and the cycle time.

### Changes in G1 and S/G2/M period size control upon forced *CLN2* expression

**Figure 8:**
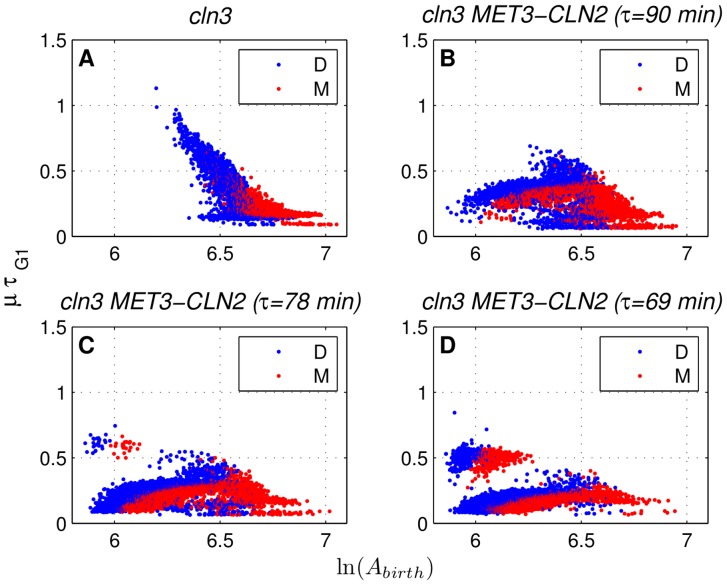
Characterization of size control in the G1 phase. Raw data from the *cln3* simulations (A) and the simulations with 90 min (B), 78 min (C), and 69 min (D) periods of forced *CLN2* expression. Cell area at birth is denoted by 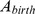, whereas 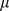 is the rate of exponential cell growth and 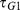 is the G1 duration. “D” and “M” stand for daughters and mothers, respectively. Simulation data is collected from eight independently generated pedigrees per forcing period.

**Figure 9:**
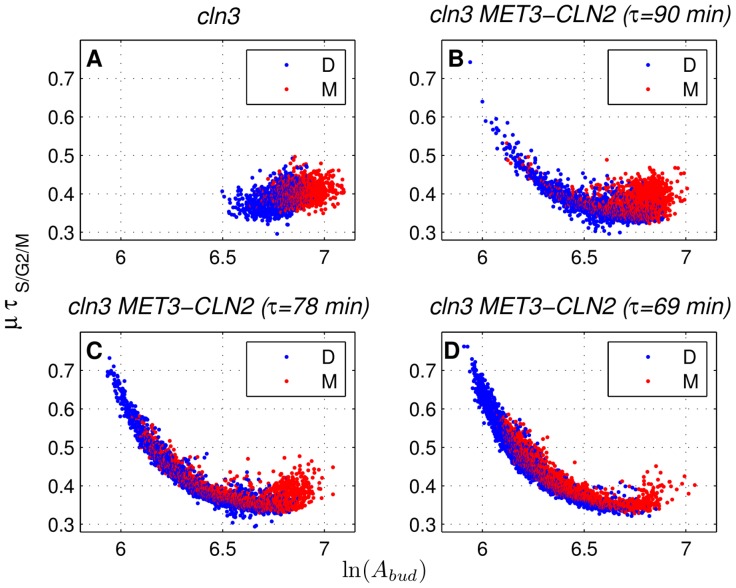
Characterization of size control in the S/G2/M phase. Raw data from the *cln3* simulations (A) and the simulations with 90 min (B), 78 min (C), and 69 min (D) periods of forced *CLN2* expression. Cell area at budding is denoted by 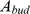, whereas 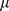 is the rate of exponential cell growth and 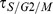 is the budded period duration. “D” and “M” stand for daughters and mothers, respectively. Simulation data is collected from eight independently generated pedigrees per forcing period.

Cell size is an important feature of cell physiology coregulating cell growth and cell division [18]. Size control in budding yeast, which results in longer G1 durations (and cycle times) for cells that are born small compared to others, has been documented previously [10], [19]. Such dependence of G1 duration on the cell size at birth (indicated by a negative slope) is also evident in our *cln3* simulations (no forced *CLN2* expression) for daughters and to a smaller extent, for mothers as shown in Figure 8A. On the other hand, with forced expression of *CLN2* (period of 90 min), budding is triggered externally and G1 size control is reduced to a great extent (compare Figures 8A and 8B). This change in G1 size control, which is more significant for daughters, agrees qualitatively with the experimental results (top row of Figure S2C in [7]). Next, we look at the changes in size control during the budded period (S/G2/M) upon forced *CLN2* expression. Similar to G1 size control, if the duration of the budded period shows dependence on the cell size at budding (the smaller the cell size at budding, the longer the budded period), we can say that budded period size control is present. As shown in Figure 9A, *cln3* simulations do not exhibit such behavior for mothers or daughters. However, once *CLN2* expression pulses are administered into the system with a period of 90 min (Figure 9B), small mothers and daughters (based on size at budding) appear. These small cells have extended budded periods compared to the cells with no forced *CLN2* expression. This qualitative observation was also reported in [7] (Figure S2C, bottom row). Such a change in the budded period size control, taken together with the reduction in the G1 size control shows that forced *CLN2* expression displaces the size control from G1 to S/G2/M. We note that the inverse of such a displacement of size control is observed in fission yeast [20], [21] by the deletion of Wee1 kinase that influences the timing of mitosis. Lack of Wee1 (*wee1*) causes early mitosis (small cell size at division). On the other hand, *WEE1* overexpression delays mitosis (large cell size at division). *wee1* mutants exhibit G1 size control, whereas wild type cells of fission yeast skip this size control, instead G2/M size control operates. These examples demonstrate the adaptive nature of size control in yeast when mutations (e.g., *wee1* in fission yeast) or dynamic perturbations (e.g., *cln3 MET3-CLN2* in budding yeast) are applied to the system. Such adaptive size control allows cells to compensate for perturbations to their natural state and prevents cell size from becoming too small or too large [22], either of which can lead to cell death.

Next, we quantify the changes in size control during G1 and S/G2/M upon forced *CLN2* expression as described in the Methods. Prior to this, the simulation data is binned as previously done in [7], [10]. Results of binning for *cln3 MET3-CLN2* and *cln3* simulation data are shown in Figures S7 (for G1 size control) and S8 (for S/G2/M size control). 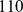 cells are collected per bin after sorting the simulation data points (in ascending order) in terms of cell areas at birth (Figure S7) and at budding (Figure S8). Table 7 shows the percent changes in the G1 and S/G2/M size control (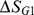 and 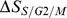) upon forced *CLN2* expression with different forcing periods. We see that at least 90% of the G1 size control is eliminated in daughters (with forcing periods of 90, 78, and 69 min) and in mothers (with forcing periods of 78 and 69 min). The only exception is the 41% drop in the mother G1 size control with 90 min period *CLN2* expression pulses, since a majority of the mother cells escape these slow pulses due to their much faster cycle time: the fraction of locked mother cells with forcing period of 90 min is less than 0.5 in the simulations. On the other hand, the increase in the strength of the budded period size control becomes more pronounced as *CLN2* expression pulses come more frequently (shorter pulse periods). We observe at least a 258% increase in the budded period size control for both mothers and daughters with three pulse periods, the only exception again being mothers with forcing period of 90 min (112% increase). The strength of the budded period size control in small mothers and daughters (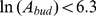 in Figures 9B, 9C, and 9D), ranges 0.54–0.58 for mothers and 0.63–0.75 for daughters with forcing periods of 90, 78, and 69 min. In other words, our model predicts that the strength of this size control on small cells (based on size at budding) is consistent regardless of the exact pulse period. The increase in the fraction of cells budding at a small size (Table 7, rightmost column) with decreased pulse period causes the overall change in the size control strength (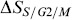) to go up. We also note that the range 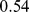
–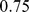 of the size control strength 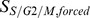 on these small mothers and daughters aligns with the strength of G1 size control (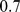) reported in [10].

**Table 7:** Changes in G1 and S/G2/M size control upon forced *CLN2* expression with different forcing periods.

	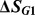	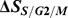	Percentage of small cells at budding
D (90 min period)	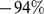	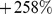	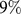
M (90 min period)	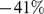	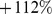	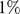
D (78 min period)	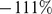	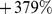	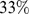
M (78 min period)	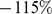	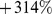	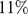
D (69 min period)	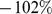	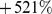	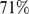
M (69 min period)	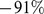	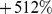	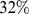

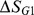
 and 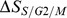 (both defined in the Methods section) quantify the changes in the size control strength in G1 and S/G2/M phases, respectively for mother (M) or daughter (D) cells. For small cells at budding (in Figures 9B, 9C, and 9D), 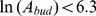. Here, the cell area at budding is denoted by 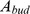. The percentage of small cells at budding increases as the pulses become more frequent. Changes in size control are computed from the aggregation of eight independently generated pedigrees per forcing period.

### Model predicts bimodality of G1 duration under frequent *CLN2* expression pulses

G1 duration is unimodal in *cln3* (no forcing) simulations (Figure 8A) and *cln3 MET3-CLN2* simulations with 90 min forcing period (Figure 8B) whereas it exhibits bimodality among cells that are small at birth with forcing periods of 78 and 69 min (Figures 8C and 8D). This bimodality is a model prediction for which no experimental data is reported. We only know that experimentally G1 duration is not bimodal, but unimodal, for *cln3* and *cln3 MET3-CLN2* (forcing period of 90 min) according to Figure S2B in [7], which aligns with the simulation data in Figures 8A and 8B.

Here, we will investigate the reason behind the bimodality observed in the simulations with 78 and 69 min period *CLN2* expression pulses. First, we modify the simulation code so that small-born cells with long and short G1 durations can be grouped separately and the extent of overlap between the *CLN2* expression pulses and the G1 periods of cells can be recorded. Then, we repeat the simulations with forcing periods of 78 and 69 min. For each period value, a group of eight pedigrees are generated (about 5000 cycles for each group of pedigrees) before collecting the data of cell sizes at birth and G1 durations. For clarity, we recall that each division event gives rise to two offspring cells: the larger cell (with 58% mean and 2.9% standard deviation of the dividing cell's size) is called the mother cell, and the smaller cell (with the remaining part of the dividing cell's size) is called the daughter cell. These newborn cells spend a certain period of time in G1 phase (the unbudded period of the cell cycle). Then, they initiate a new round of DNA synthesis and produce a new bud nearly simultaneously, entering the budded period of the cell cycle (S-G2-M phases). At the end of the budded period, the cell divides asymmetrically to form the next generation of mother and daughter cells. Dividing cells can be classified as “daughter” or “mother” cells, depending on the label they received when they were born.

In Figure S9A (forcing period of 78 min), cells in the green box have short G1 durations (depicted by the green bars in Figures S9D and S9E) because they happen to be born (the left end of each green bar) shortly before or after the start point of a *CLN2* expression pulse, which induces each cell to bud and start a new round of DNA synthesis more-or-less synchronously with the end of the *CLN2* expression pulse (the right end of each green bar). Cells in the orange box in Figures S9A have long G1 durations (the orange bars in Figures S9B and S9C) because they happen to be born shortly after the end point of a *CLN2* expression pulse, and they remain in G1 phase (unbudded) for about 60 min, when the next *CLN2* expression pulse arrives and induces them to bud (the right end of each orange bar in Figures S9B and S9C).

To understand why cells in the orange box are born right after a *CLN2* pulse, we examined the parents of these small-born cells with long G1 periods. Surprisingly, all of these parents were found in the green box in Figure S9A. In other words, it is only small-born cells with short G1 periods that give birth to small-born cells with long G1 periods. A common feature of these parent cells was their extended budded period (S-G2-M phase) with an average greater than 75 min, whereas the average budded period of all parent cells in the simulations was less than 50 min. The cause of this extended budded period is the “budded period size control” (depicted in Figure 9C), whereby small-born cells with short G1 periods tend to compensate by having a long budded period. We have already seen that small-born cells with short G1 (i.e., cells in the green box in Figure S9A) tend to enter the budded phase of the cell cycle at the end of a *CLN2* pulse. Since the duration of their budded period is greater than 75 min on average, they tend to divide at the end of the following *CLN2* pulse, in which case they give rise occasionally to small progeny cells that are born at the end of a *CLN2* pulse. These are the 42 cells in the orange box (Figure S9A); cells that are small-born and have a long G1 phase. Of these 23 daughter cells and 19 mother cells in this box, there are 17 mother-daughter pairs derived from the same parent cell in the green box, and almost all of these parent cells are themselves “daughters”. These correlations are due to the fact that daughter cells are more likely than mother cells to be small-born (compare the relative abundances of blue and red dots in the green box of Figure S9A) and then experience an extended budded phase. When they divide, these cells are still smaller than average, giving rise to small progeny cells (mothers and daughters) that predominantly populate the orange box in Figure S9A.

Figures S9B and S9C reveal another interesting feature of small-born cells with long G1 durations. These cells were born during the second half of the 700 min simulations, because budded period size control affects cells that are smaller at budding more than the larger cells born earlier. Detailed statistics of G1 duration bimodality with 78 min period pulses are summarized in Table S12. About 1% of all the cells (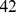 of 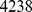) are small-born and have long G1 durations. The extent of such cells increases to 12% with only a 9 min drop in the forcing period (69 min). This change is due to size control: the percentage of cells affected by size control during the budded period (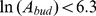 in Figures 9B, 9C, and 9D) with a 69 min forcing period is more than double of the same percentage with the 78 min period for mothers and daughters (Table 7). Figure S10 and Table S12 give a close look at the G1 duration bimodality with a 69 min forcing period. The results are similar to those for the 78 min forcing period with the only exception of the aforementioned higher fraction of small-born cells with long G1 duration among the whole population.

As we previously mentioned, according to Figure 6B, 69 and 78 min forcing periods are better choices than the 90 min period for synchronizing the mother cells in budding yeast populations. 69 and 78 min periods favor mother cells since they are more aligned with the natural mother cycle time (71 min). However, according to the model predictions, 69 and 78 min forcing periods are also the only periods that give rise to bimodality of G1 duration among small-born cells given birth by small-born daughters with extended budded periods. Such bimodality works against synchronization compared to an otherwise unimodal G1 distribution among small-born cells, which supports synchronization. This points out an important trade-off between increased synchronization levels among mother cells with more frequent *CLN2* expression pulses versus unimodality of G1 duration with longer periods that are too slow to affect (or lock) mother cycles. Hence, the asymmetric nature of cell division in budding yeast (resulting in shorter mother cycle times), which is an adaptive trait under nutrient limitations [23], [24] and necessary for cell diversity [25], seems to be a critical factor setting an upper limit to the level of synchronization within cell colonies for a given strength and duration of forced *CLN2* expression from the *MET3* promoter.

As shown in Table S13, with 78 min forcing period, increasing the promoter strength from medium level (1) to high level (4) leads to an 84% increase in the level of synchronization. However, this increased level of synchrony among the cells comes at the expense of cycle failures (4% of the cycles fail due to incorrect order of cell cycle events). These failures originate from the “Execution Error 4” in Table S9, namely the alignment of SPN before bud emergence. The premature alignment of SPN is caused by high Cln2 activity (due to strong strength of *CLN2* expression pulses) resulting in high Clb5 and Clb2 activities. Cln2 is an activator of Clb5, Clb5 is an activator of Clb2, whereas Clb2 directly controls (activates) spindle alignment that is quantified by SPN. The high activity levels of Clb5 and Clb2 activities are temporary due to a negative feedback loop. Because high Clb2 activity suppresses the high *CLB5* and *CLN2* expressions by inhibiting SBF (transcription factor for Cln2 and Clb5 synthesis). This makes a major impact on the speed of budding since Cln2 and Clb5 are the driving forces behind budding. In simple terms, with high strength *CLN2* expression pulses, budding event cannot match the speed-up (compared to medium strength pulses) of the spindle alignment and other cell cycle events.

According to Table S14, increasing the pulse duration from 20 to 30 min with 78 min forcing period has a negligible effect on the synchronization level, whereas 40 min pulses result in a 36% increase in 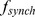. Further increase in the pulse duration decreases the level of synchrony among the cells because these longer pulses overlap not only with the unbudded G1 period but also with the budded S/G2/M period of the cycles. S/G2/M period is unresponsive to cyclin expression pulses since the cells are already budded in this period. Interestingly, 40 min pulse duration period beyond which 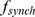 drops, coincides with the G1 duration of daughter cells in the simulations with 78 min period pulses (Table 3) supporting our conclusion regarding the unresponsiveness of cells to periodic *CLN2* expression pulses beyond the 40 min pulse duration.

A common trend observed in Tables S13 and S14 is that the variability of 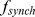 increases as we move away from the optimal pulse conditions (weaker *MET3* promoter strength and shorter/longer pulse duration). The only exception is the lowered CV of 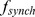 with high *MET3* promoter strength (0.24 with medium strength to 0.17 with high strength). However, this decreased variability and higher mean 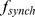 value (0.25 with medium strength and 0.46 with high strength) comes at the expense of cycle failures, hence not adhering with the optimality criterion of failure-free cycles. The observed higher 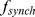 variability as the forcing conditions diverge from the optimal conditions support our conclusions regarding the optimal promoter strength (medium) and optimal pulse duration (20–40 min).

## Conclusions

In this study, we use a stochastic differential equation model to explore the potential of periodically forced expression of *CLN2* cyclin to synchronize the cell division cycle of budding yeast cells. We calibrate this model by constraining its deterministic version with the observed phenotypes of 110 mutant strains while simultaneously fitting the statistical properties of asynchronous populations of cells to the stochastic aspects of the model. Calibration of the model with these extensive data sets allows us to constrain the model parameters and make credible predictions regarding the changes in cell cycle dynamics upon forced periodic expression of *CLN2* while comparing our results with the experimental findings in [7]. Without further adjustment of parameter values, the stochastic model correctly predicts among mother and daughter cells under different experimental conditions: (1) levels of synchronization, (2) changes in the cell cycle statistics, and (3) changes in the size control during the G1 and budded periods. The model also predicts that under frequent *CLN2* expression pulses, small-born daughter cells that bud quickly have an extended budded period that results in an extended G1 period in the following cycle among some cells, hence leading to bimodality of G1 duration among small-born cells.

Our results provide insights into the interplay between cell cycle dynamics, size control, and synchronization of cell colonies under cyclin expression pulses. Our approach to validating a complex budding yeast cell cycle model is an important example for the integration of data sets from a wide variety of experiments into a predictive model of an important biological control system. We believe that by further exploring the model response under a variety of inputs, we can guide the future experimental research in identifying optimal inputs that drive budding yeast cell populations into high levels of synchrony.

## Supporting Information

Figure S1 **Budding index trajectories under different conditions.** Evolution of the budding index for the unforced cells (*cln3*, green lines) and the cells with forced *CLN2* expression with forcing period of 78 min (*cln3 MET3-CLN2*: black lines, *MET3-CLN2*: red lines). Each individual trajectory represents a colony initiated by a single daughter cell. The blue shaded areas represent the time intervals in which *MET3-CLN2* is active (time lag for the MET3 promoter turn-on/turn-off is taken into account).
(TIFF)

Figure S2 **Simulated return maps of asynchronous cells.** Control return maps with periods of 90 min (in A and D), 69 min (in B and E), and 60 min (in C and F). “D” and “M” stand for daughters and mothers, respectively. All maps are with no forced *CLN2* expression (*cln3* cells). Colors represent the fraction of data points in each map region as depicted in the color map on the right. Only the bright colors of this map are used in the return maps except for the map regions with very low data density.
(TIFF)

Figure S3
**Color density plot of mean fitting error for locking time ranges.** Mean absolute error is 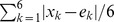, where 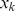 and 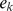 are the 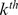 locked fraction values in simulations and experiments, respectively. Here, we have six data points: three pulse periods (90, 78, and 69 min), each with a daughter and mother locked fraction. Each candidate locking range is a point on the 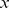-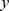 plane. The 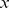 axis represents the minimum value of the time range, whereas the 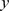 axis represents the maximum. Ranges are 10–22 min long and are generated by LH sampling. Optimal locking regime for the model is depicted by the red square in the lower left corner.(TIFF)

Figure S4
**Simple vs. complex** *MET3* **promoter dynamics.** With complex promoter dynamics, periodic *CLN2* expression from the *MET3* promoter is gradually turned on and gradually turned off (represented by a parabolic function described in Text S2), whereas the simpler promoter dynamics that exhibit immediate turn on and turn off are represented by a step function. 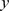 axis represents the promoter activity which evolves as a fraction of the maximum promoter activity with respect to time.(TIFF)

Figure S5
**Fractions of locked daughters and mothers with simple and complex promoter dynamics.** Forced *CLN2* expression with six forcing periods: simulation values for daughters (in A) and mothers (in B). Black vertical lines represent the natural (*cln3*, no forced *CLN2* expression) mother and daughter cycle times. The range of each locked fraction in the simulations (mean 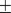 standard deviation) is depicted by the blue error bars with simple promoter dynamics (square pulses), whereas the red bars correspond to the ranges of locked fractions with complex promoter dynamics (parabolic pulses). Each range from the simulations is computed from 15 independent realizations. Each realization contains eight independently generated pedigrees of cells generated over the course of 700 min starting from a single daughter or mother cell.(TIFF)

Figure S6
**Size calibration curve.** This curve is used to convert simulation cell size (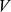) to the probable experimental cell area (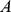) in size control analysis. Best linear fit is extracted from six data points: average mother and daughter cell size/area values at birth and budding, with and without forced *CLN2* expression (forcing period of 90 min). Experimental cell area values are from [7].(TIFF)

Figure S7
**Characterization of size control in the G1 phase.** Binned simulation data (110 cells per bin) from the *cln3* simulations (A) and the simulations with 90 min (B), 78 min (C), and 69 min (D) periods of forced *CLN2* expression. Cell area at birth is denoted by 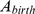, whereas 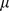 is the rate of exponential cell growth, and 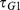 is the G1 duration. Mean and standard deviation values for each bin are depicted by circles and vertical lines, respectively. Thick black lines show the best linear fits. “D” and “M” stand for daughters and mothers, respectively.(TIFF)

Figure S8
**Characterization of size control in the S/G2/M phase.** Binned simulation data (110 cells per bin) from the *cln3* simulations (A) and the simulations with 90 min (B), 78 min (C), and 69 min (D) periods of forced *CLN2* expression. Cell area at budding is denoted by 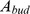, whereas 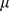 is the rate of exponential cell growth, and 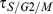 is the budded period duration. Mean and standard deviation values for each bin are depicted by circles and vertical lines, respectively. Thick black lines show the best linear fits. “D” and “M” stand for daughters and mothers, respectively.(TIFF)

Figure S9
**Predicted bimodality of G1 duration with 78 min forcing period.** (A) Model predicts bimodal G1 duration (time elapsed from cell birth to budding) among cells with small birth size under forced *CLN2* expression. Unbudded G1 periods are represented by horizontal orange lines for cells with long G1 durations, whereas green lines represent the G1 periods for cells with short G1 durations. Middle column (B and D): daughter cells, right column (C and E): mother cells. 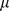 is the rate of exponential cell growth, and 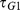 is the G1 duration. The blue shaded areas represent the time intervals in which *MET3-CLN2* is active (time lag for the MET3 promoter turn-on/turn-off is taken into account).(TIFF)

Figure S10
**Predicted bimodality of G1 duration with 69 min forcing period.** (A) Model predicts bimodal G1 duration (time elapsed from cell birth to budding) among cells with small birth size under forced *CLN2* expression. Unbudded G1 periods are represented by horizontal orange lines for cells with long G1 durations, whereas green lines represent the G1 periods for cells with short G1 durations. Middle column (B and D): daughter cells, right column: mother cells (C and E). 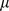 is the rate of exponential cell growth, and 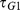 is the G1 duration. The blue shaded areas represent the time intervals in which *MET3-CLN2* is active (time lag for the MET3 promoter turn-on/turn-off is taken into account).(TIFF)

File S1 **The zip file contains Matlab and C++ scripts with MEX files that reproduce the cell cycle statistics listed in** **Tables 1** **,** **2** **, and** **3** **.** File A is Matlab script that reproduces the statistics of *cln3* simulations (rightmost column of Table 1). An individual text file for each statistical data point is generated upon execution of File A. File B is Matlab script that reproduces the statistics of wild type simulations (rightmost column of Table 2). An individual text file for each statistical data point is generated upon execution of File B. File C is a Matlab script that reproduces the statistics of *cln3 MET3-CLN2* simulations (forcing period of 90 min) with medium *MET3* strength (Table 3: fourth column from the left). An individual text file for each statistical data point is generated upon execution of File C. File D is a Matlab file that needs to be renamed to “findnearest.m” for the execution of Files A, B, and C. File E is a data file that needs to be renamed to “paramdeterm.txt” for the execution of Files A, B, and C. File F is a data file that needs to be renamed to “uoptim.txt” for the execution of Files A, B, and C. File G is a data file that needs to be renamed to “numbmolectime0cln3del.txt” for the execution of Files A and C. File H is a data file that needs to be renamed to “numbmolectime0.txt” for the execution of File B. File I is a C++ file that needs to be renamed to “integratepulse.cpp” for the execution of File C. File J is a MEX file that needs to be renamed to “integratepulse.mexw64” for the execution of File C. File K is a C++ file that needs to be renamed to “integrateic.cpp” for the execution of Files A, B, and C. File L is a MEX file that needs to be renamed to “integrateic.mexw64” for the execution of Files A, B, and C. File M is a C++ file needs to be renamed to “integrate.cpp” for the execution of Files A and B. File N is a MEX file that needs to be renamed to “integrate.mexw64” for the execution of Files A and B.
(ZIP)

Table S1 **Deterministic model variables.** Each variable corresponds to a single ODE. Deterministic model variables, which represent the concentration values, are converted to stochastic model variables that represent the numbers of molecules. The conversion process is described in [8], [11].
(PDF)

Table S2 **Parameters exclusive to the stochastic model.** These parameters are only present in the stochastic model equations.
(PDF)

Table S3 **List of 119 phenotypes.**
(PDF)

Table S4 **Nine phenotypes that are not captured by the deterministic model.** Mismatches between the deterministic simulations and the experimental phenotypes. In the simulations, double-period oscillations are considered inviable even if the events are executed in the right order.
(PDF)

Table S5
**Cell cycle events in the deterministic simulations.** Each event corresponds to a concentration value and its specific threshold. Subscript 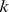 stands for the current time step, whereas 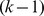 represents the previous time step in the simulations. Events 2 and 3 (also 4 and 5) can interchange order without the loss of viability.(PDF)

Table S6 **Parameters that are present both in deterministic and stochastic models.** These parameter values capture 110 phenotypes (out of 119 phenotypes in Table S3) with deterministic simulations. Phenotypes that are listed in Table S4 are not captured.
(PDF)

Table S7
**Values of parameters that are present both in deterministic and stochastic models.** In the stochastic simulations, the daughter cell mass retained after each division (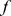) is the remainder of the total mass after the mother fraction is randomly assigned from a normal distribution with 0.58 mean and 0.029 standard deviation.(PDF)

Table S8
**Protein abundances.** Abundances of some of the key cell cycle proteins: experimental vs. model values. Abundance data is collected in the simulations as described in [8] on page 59. Simulation statistics (mean 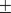 standard deviation) are computed from 15 realizations. In each realization, twenty pedigrees are generated independently. Each pedigree of cells is initiated by a single daughter (D) or mother (M) cell. In some cases, experimental abundance data from diploid cells are halved for approximating the abundances in haploid cells.(PDF)

Table S9 **The correct order of the cell cycle events in the stochastic simulations are enforced by checking for the presences of execution errors.** Event checking is based on the concentration thresholds (for ORI, SBF, Esp1, SPN, BUD, and Clb2) listed in Table S5. The numbers of molecules recorded in the stochastic simulations are converted to concentrations for this purpose. Once an execution error in a cycle is detected, the pedigree cannot continue from the particular cell that have executed the event incorrectly (no progeny can be born from this cell). In that case, the cycle is recorded as a failed cycle.
(PDF)

Table S10 **Initial condition set.** Initial concentration values in the deterministic simulations and the initial numbers of molecules in the stochastic simulations (in parentheses). The conversion of concentrations to numbers of molecules is explained in [8], [11]. In *cln3* stochastic simulations, the initial number of Cln3 molecules is set to zero, other values are shown as above. For *MET3-CLN2 cln3* stochastic simulations (with forced *CLN2* expression), the initial numbers of molecules come from the end points of 2000 min *cln3* simulations in order to mimic the experimental conditions in [7].
(PDF)

Table S11
**Start/end time points (min) of the forced** *CLN2* **expression pulses.** 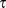 is the period of forced *CLN2* expression (in minutes). Start and end points of the pulses (in minutes) with *MET3* promoter delay decide the actual time intervals during which *CLN2* expression pulses are administered into the system during the simulations. Start time points without promoter delay are used to compute the time between the closest starting point and budding in the current cycle (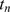) and the subsequent cycle (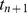), as it was done in the experimental study [7]. During *cln3* (control) simulations, forced *CLN2* expression is not present. In these simulations, 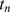 and 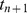 are computed as the differences between the time points of budding in the current and subsequent cycles with the closest preceding pulse start points without promoter delay, respectively (Figure S2 and Figures 4A and 4C). This procedure was also followed in the experimental study [7]. With or without forced *CLN2* expression, the closest pulse start time that is selected to compute 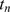 (or 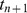) is before the time point of budding [7] (i.e., start point precedes budding).(PDF)

Table S12 **Summary of the statistics regarding the G1 duration bimodality observed in Figures S9 and S10.** Statistics of the small-born cells exhibiting bimodality of the G1 duration with 78 and 69 min periods of forced *CLN2* expression.
(PDF)

Table S13
**Synchronization levels with different** *MET3pr* **strengths (forcing period of 78 min).** Pulse duration is 20 min. 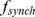 is the fraction of time points (between 300–700 min in the simulations) at which more than 95% or less than 5% of the cells are budded. The simulation statistics (mean 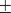 standard deviation) are computed from 15 independent realizations per promoter strength. In each realization, a budding index trajectory is generated from a pedigree. Each trajectory starts from a single cell and the number of cells within the pedigree increases exponentially due to cell division. The number of the failed cycles (due to event execution errors listed in Table S9) normalized by the number of complete cycles is the cycle failure ratio.(PDF)

Table S14
**Synchronization levels with different pulse durations (medium** *MET3pr* **strength, forcing period of 78 min).** 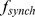 is the fraction of time points (between 300–700 min in the simulations) at which more than 95% or less than 5% of the cells are budded. The simulation statistics (mean 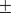 standard deviation) are computed from 15 independent realizations per promoter strength. In each realization, a budding index trajectory is generated from a pedigree. Each trajectory starts from a single cell and the number of cells within the pedigree increases exponentially due to cell division. The number of the failed cycles (due to event execution errors listed in Table S9) normalized by the number of complete cycles is the cycle failure ratio.(PDF)

Text S1 **Identification of the model's locking time window.**
(PDF)

Text S2 **Model predictions with complex** *MET3* **promoter dynamics.**
(PDF)
