# Supplementary figures and images for: A Stochastic Model Correctly Predicts Changes in Budding Yeast Cell Cycle Dynamics upon Periodic Expression of CLN2
Source: PLoS One. 2014 May 9;9(5):e96726. doi: 10.1371/journal.pone.0096726 (PMC4016136; doi:10.1371/journal.pone.0096726)

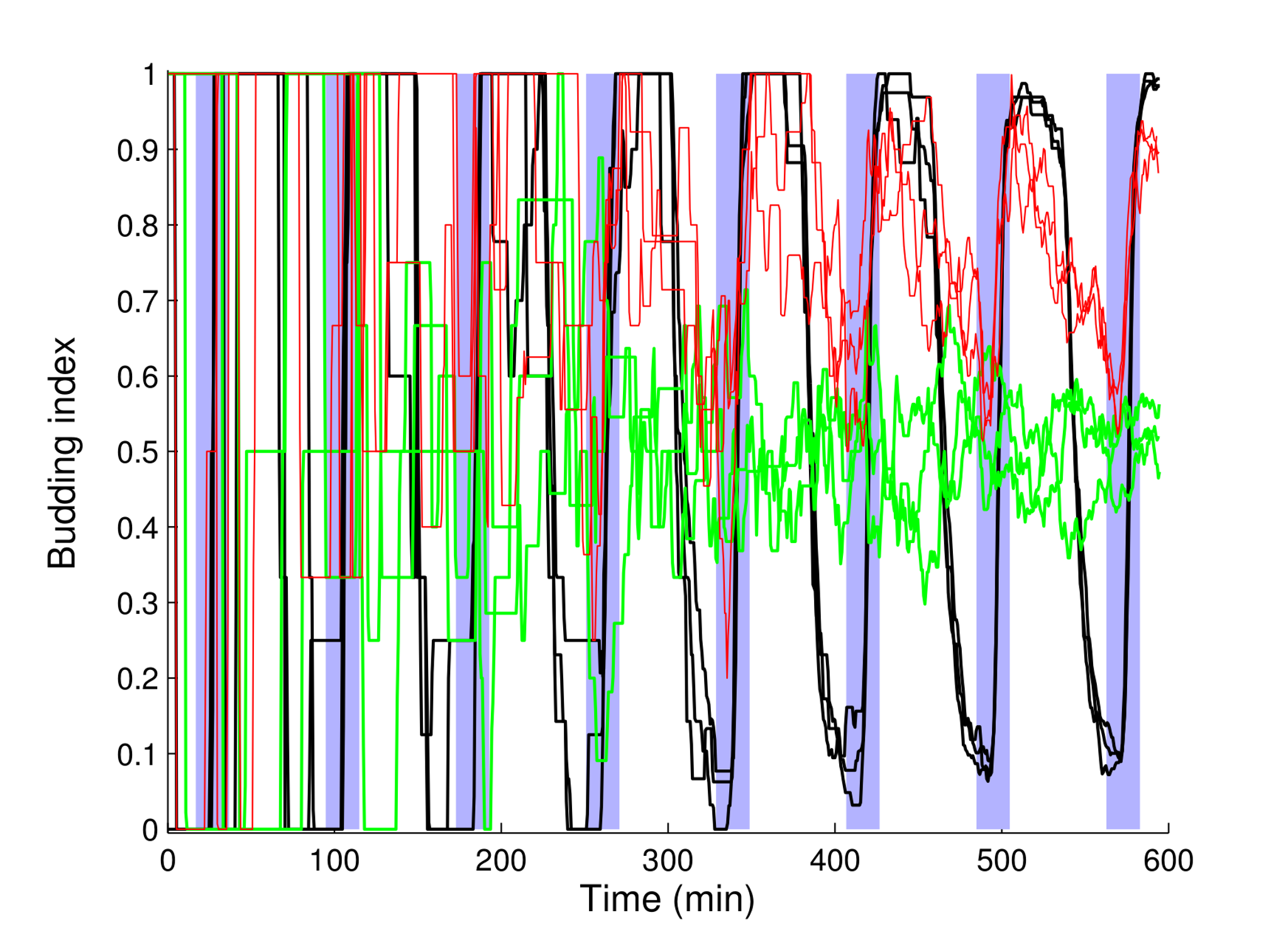

Supplement: Figure S1 — Budding index trajectories under different conditions. Evolution of the budding index for the unforced cells (cln3, green lines) and the cells with forced CLN2 expression with forcing period of 78 min (cln3 MET3-CLN2: black lines, MET3-CLN2: red lines). Each individual trajectory represents a colony initiated by a single daughter cell. The blue shaded areas represent the time intervals in which MET3-CLN2 is active (time lag for the MET3 promoter turn-on/turn-off is taken into account). (TIFF) [file pone.0096726.s001.tiff]

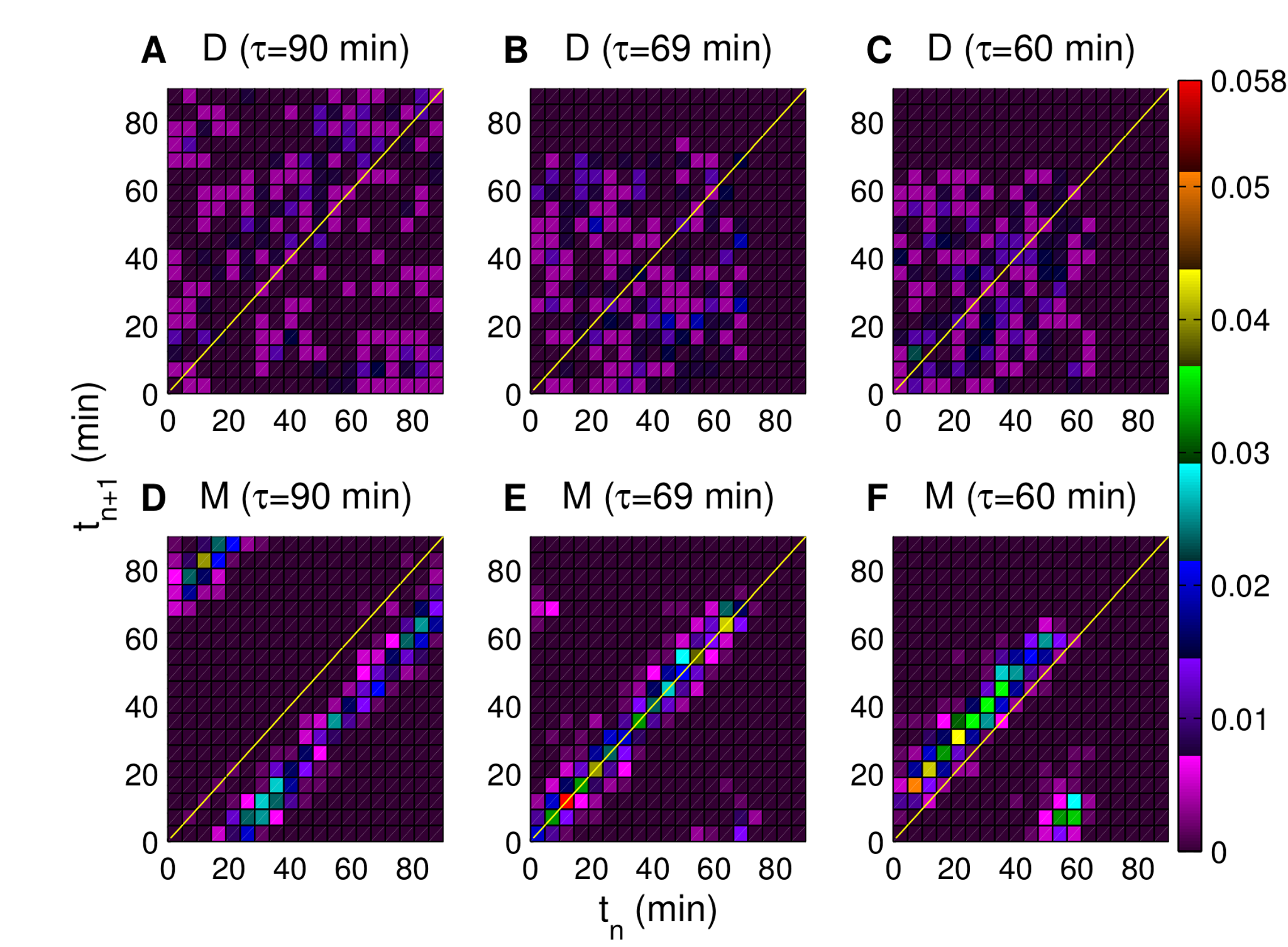

Supplement: Figure S2 — Simulated return maps of asynchronous cells. Control return maps with periods of 90 min (in A and D), 69 min (in B and E), and 60 min (in C and F). “D” and “M” stand for daughters and mothers, respectively. All maps are with no forced CLN2 expression (cln3 cells). Colors represent the fraction of data points in each map region as depicted in the color map on the right. Only the bright colors of this map are used in the return maps except for the map regions with very low data density. (TIFF) [file pone.0096726.s002.tiff]

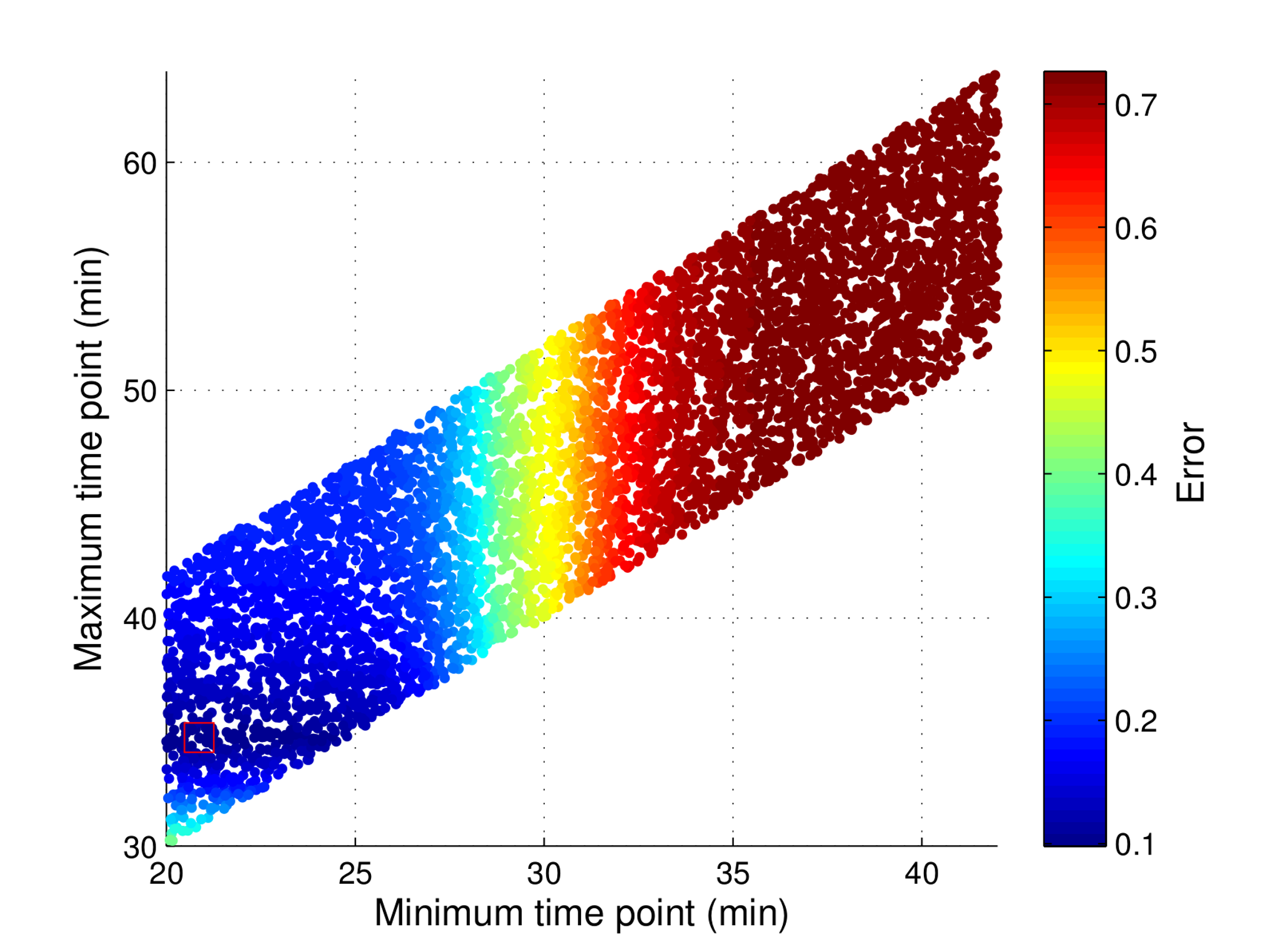

Supplement: Figure S3 — Color density plot of mean fitting error for locking time ranges. Mean absolute error is , where and are the locked fraction values in simulations and experiments, respectively. Here, we have six data points: three pulse periods (90, 78, and 69 min), each with a daughter and mother locked fraction. Each candidate locking range is a point on the - plane. The axis represents the minimum value of the time range, whereas the axis represents the maximum. Ranges are 10–22 min long and are generated by LH sampling. Optimal locking regime for the model is depicted by the red square in the lower left corner. (TIFF) [file pone.0096726.s003.tiff]

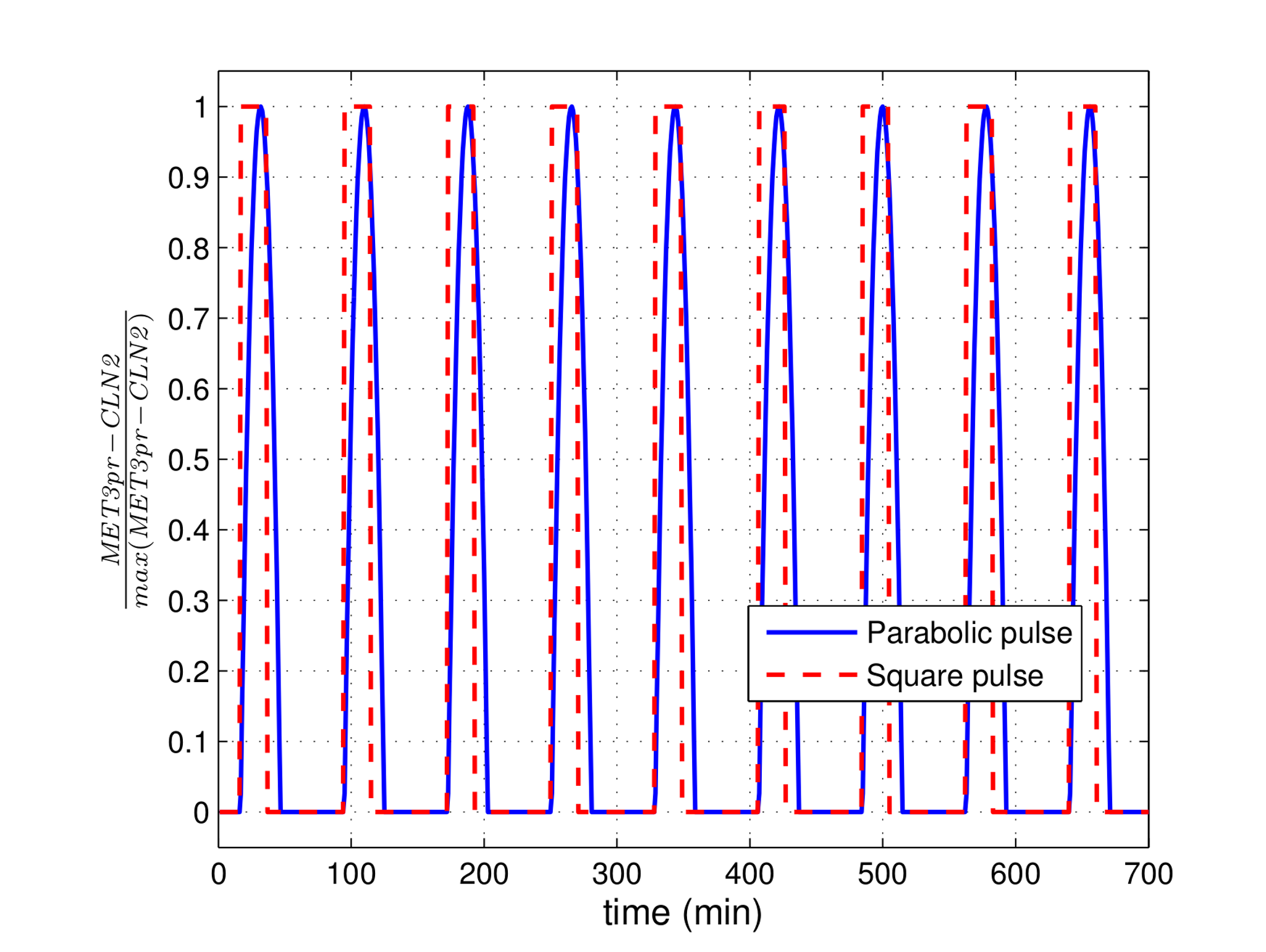

Supplement: Figure S4 — Simple vs. complex MET3 promoter dynamics. With complex promoter dynamics, periodic CLN2 expression from the MET3 promoter is gradually turned on and gradually turned off (represented by a parabolic function described in Text S2), whereas the simpler promoter dynamics that exhibit immediate turn on and turn off are represented by a step function. axis represents the promoter activity which evolves as a fraction of the maximum promoter activity with respect to time. (TIFF) [file pone.0096726.s004.tiff]

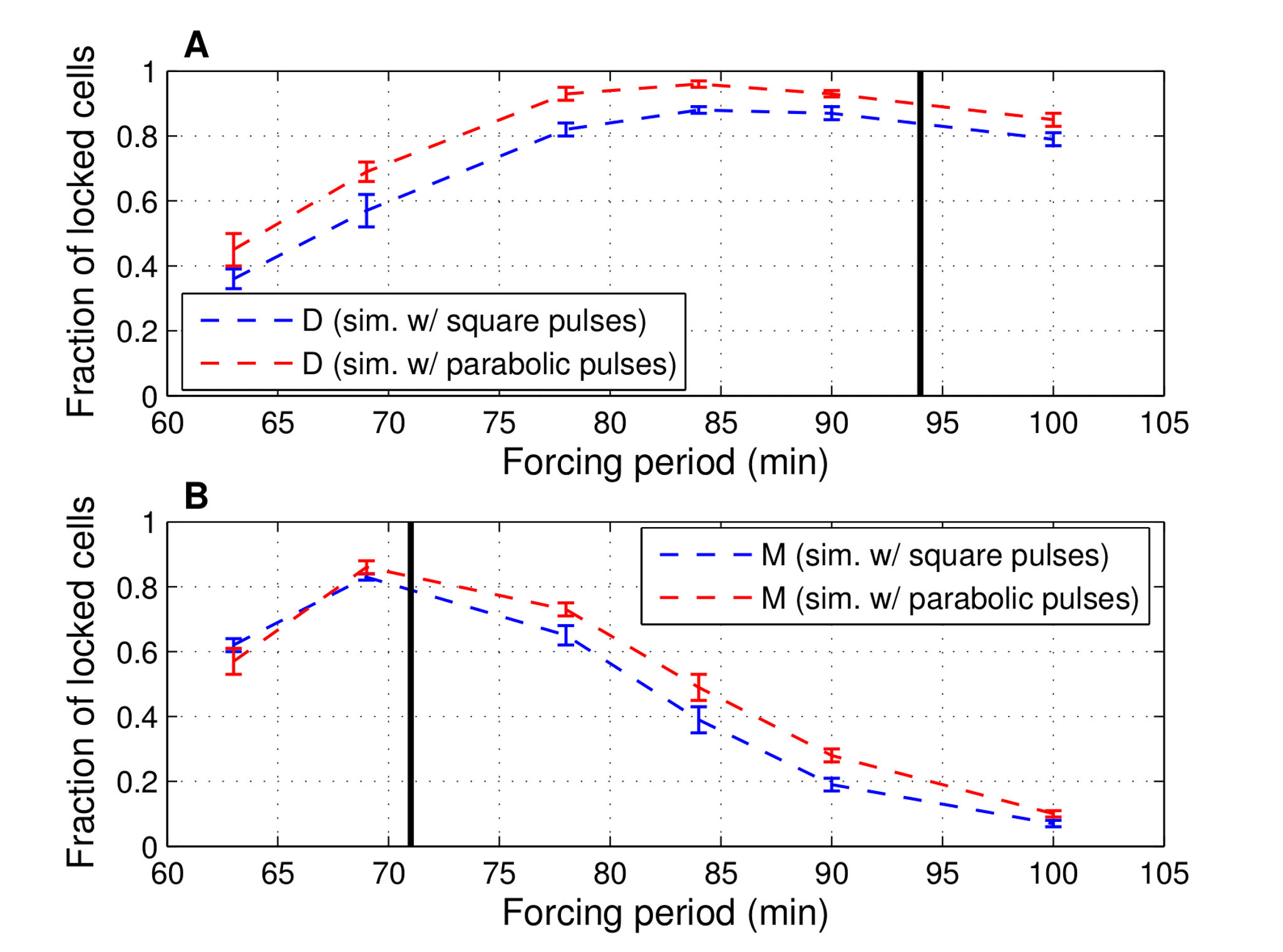

Supplement: Figure S5 — Fractions of locked daughters and mothers with simple and complex promoter dynamics. Forced CLN2 expression with six forcing periods: simulation values for daughters (in A) and mothers (in B). Black vertical lines represent the natural (cln3, no forced CLN2 expression) mother and daughter cycle times. The range of each locked fraction in the simulations (mean standard deviation) is depicted by the blue error bars with simple promoter dynamics (square pulses), whereas the red bars correspond to the ranges of locked fractions with complex promoter dynamics (parabolic pulses). Each range from the simulations is computed from 15 independent realizations. Each realization contains eight independently generated pedigrees of cells generated over the course of 700 min starting from a single daughter or mother cell. (TIFF) [file pone.0096726.s005.tiff]

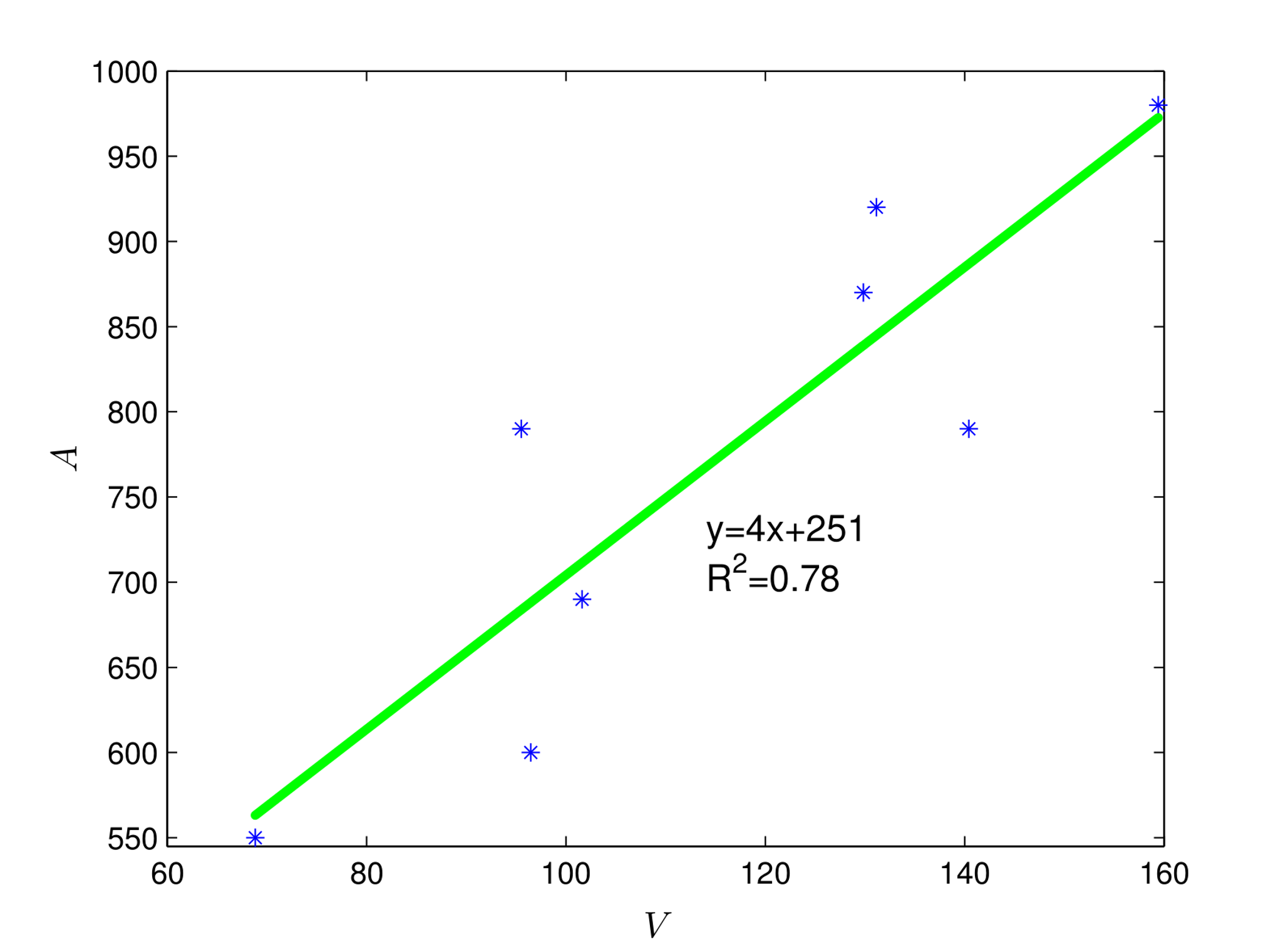

Supplement: Figure S6 — Size calibration curve. This curve is used to convert simulation cell size () to the probable experimental cell area () in size control analysis. Best linear fit is extracted from six data points: average mother and daughter cell size/area values at birth and budding, with and without forced CLN2 expression (forcing period of 90 min). Experimental cell area values are from [7]. (TIFF) [file pone.0096726.s006.tiff]

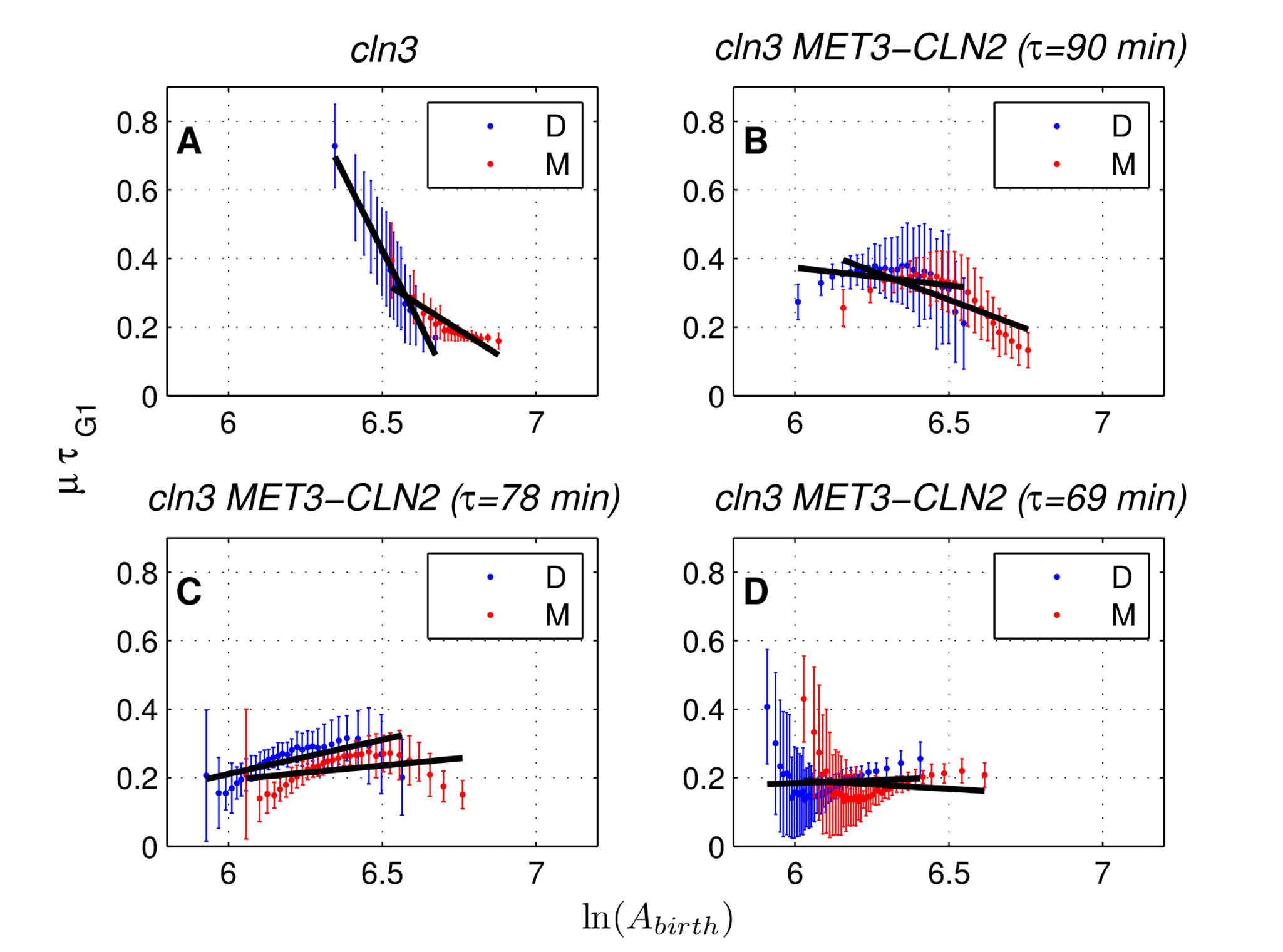

Supplement: Figure S7 — Characterization of size control in the G1 phase. Binned simulation data (110 cells per bin) from the cln3 simulations (A) and the simulations with 90 min (B), 78 min (C), and 69 min (D) periods of forced CLN2 expression. Cell area at birth is denoted by , whereas is the rate of exponential cell growth, and is the G1 duration. Mean and standard deviation values for each bin are depicted by circles and vertical lines, respectively. Thick black lines show the best linear fits. “D” and “M” stand for daughters and mothers, respectively. (TIFF) [file pone.0096726.s007.tiff]

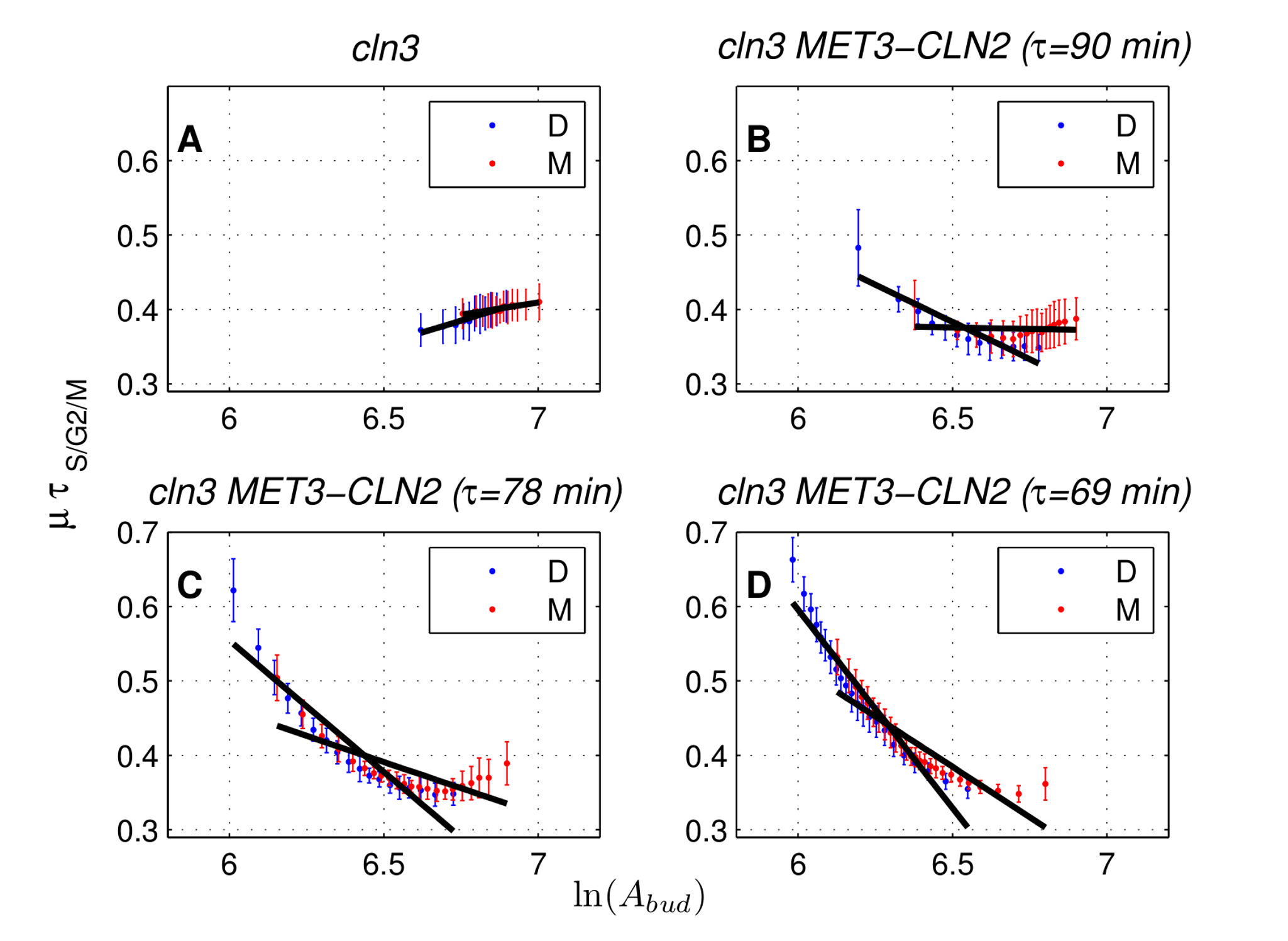

Supplement: Figure S8 — Characterization of size control in the S/G2/M phase. Binned simulation data (110 cells per bin) from the cln3 simulations (A) and the simulations with 90 min (B), 78 min (C), and 69 min (D) periods of forced CLN2 expression. Cell area at budding is denoted by , whereas is the rate of exponential cell growth, and is the budded period duration. Mean and standard deviation values for each bin are depicted by circles and vertical lines, respectively. Thick black lines show the best linear fits. “D” and “M” stand for daughters and mothers, respectively. (TIFF) [file pone.0096726.s008.tiff]

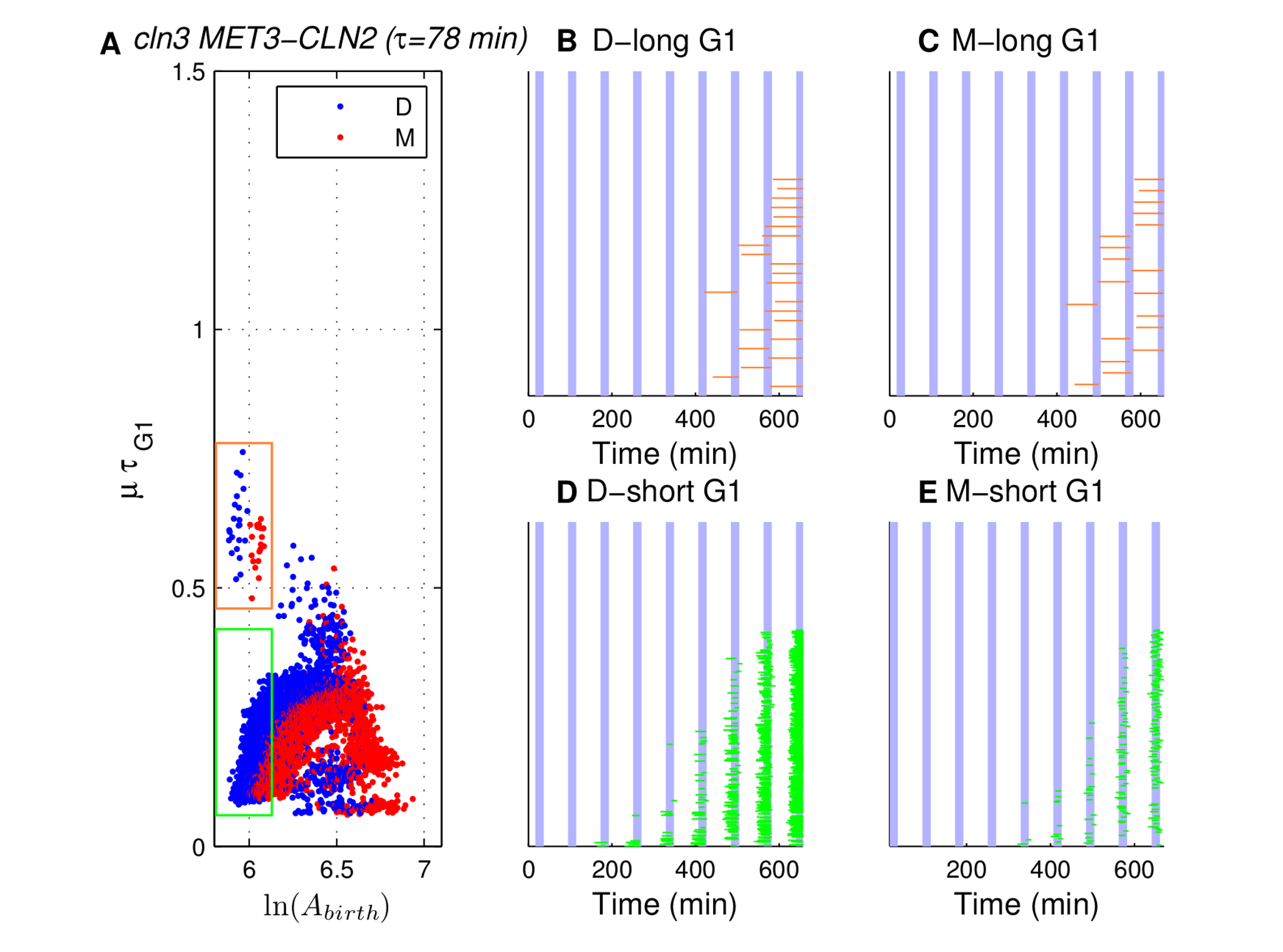

Supplement: Figure S9 — Predicted bimodality of G1 duration with 78 min forcing period. (A) Model predicts bimodal G1 duration (time elapsed from cell birth to budding) among cells with small birth size under forced CLN2 expression. Unbudded G1 periods are represented by horizontal orange lines for cells with long G1 durations, whereas green lines represent the G1 periods for cells with short G1 durations. Middle column (B and D): daughter cells, right column (C and E): mother cells. is the rate of exponential cell growth, and is the G1 duration. The blue shaded areas represent the time intervals in which MET3-CLN2 is active (time lag for the MET3 promoter turn-on/turn-off is taken into account). (TIFF) [file pone.0096726.s009.tiff]

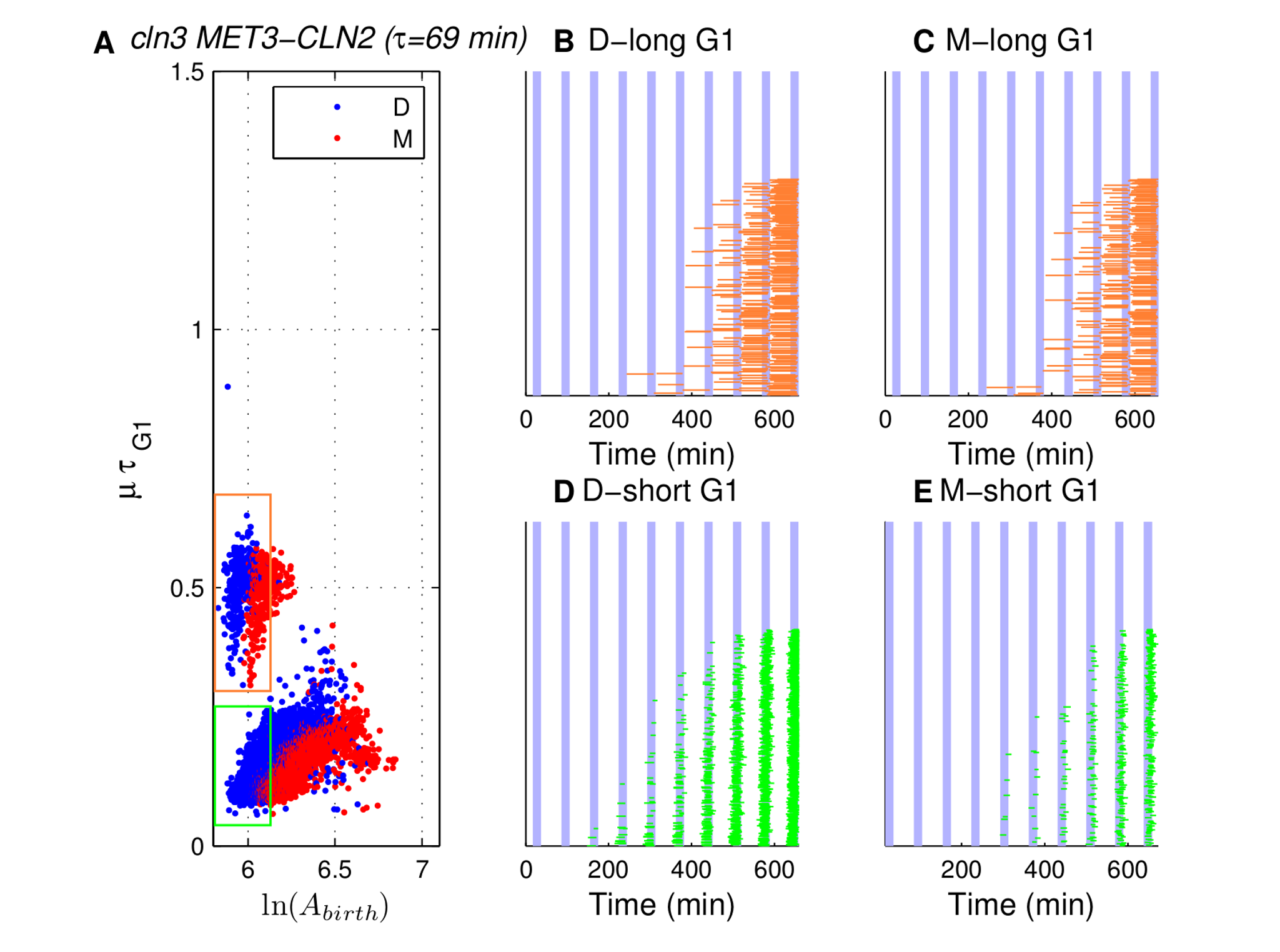

Supplement: Figure S10 — Predicted bimodality of G1 duration with 69 min forcing period. (A) Model predicts bimodal G1 duration (time elapsed from cell birth to budding) among cells with small birth size under forced CLN2 expression. Unbudded G1 periods are represented by horizontal orange lines for cells with long G1 durations, whereas green lines represent the G1 periods for cells with short G1 durations. Middle column (B and D): daughter cells, right column: mother cells (C and E). is the rate of exponential cell growth, and is the G1 duration. The blue shaded areas represent the time intervals in which MET3-CLN2 is active (time lag for the MET3 promoter turn-on/turn-off is taken into account). (TIFF) [file pone.0096726.s010.tiff]
