# Supplementary material for: A Stochastic Model Correctly Predicts Changes in Budding Yeast Cell Cycle Dynamics upon Periodic Expression of CLN2
Source: PLoS One. 2014 May 9;9(5):e96726. doi: 10.1371/journal.pone.0096726 (PMC4016136; doi:10.1371/journal.pone.0096726)
Supplement: Table S1 — Deterministic model variables. Each variable corresponds to a single ODE. Deterministic model variables, which represent the concentration values, are converted to stochastic model variables that represent the numbers of molecules. The conversion process is described in [8], [11]. (PDF) [file pone.0096726.s012.pdf]

Table S1. Deterministic model variables

Variable	Description
V	Cell size
[Cln3]	Concentration of G1 cyclin Cln3
[Bck2]	Concentration of Bck2, an activator for START transition
[WHI5dep]	Concentration of dephosphorylated (active) Whi5
[SBFdep]	Concentration of dephosphorylated SBF
[Cln2]	Total concentration of G1 cyclins Cln1,2
[CKI_T]	Total concentration of Sic1+Cdc6 (stoichiometric inhibitors of Clb5,6 and Clb1,2)
[CKI_P]	Concentration of phosphorylated form of Sic1+Cdc6
[$\mathrm{Clb5}_T$]	Total concentration of free B cyclins Clb5,6
[$\mathrm{Clb2}_T$]	Total concentration of free B cyclins Clb1,2
[BUD]	Variable for bud emergence progression
[ORI]	Variable for DNA synthesis progression
[SPN]	Variable for spindle assembly progression
[$\mathrm{Swi5}_T$]	Total concentration (number of molecules) of Swi5, transcription factor for CKI synthesis
[$\mathrm{CDC20}_T$]	Total concentration of Cdc20, a protein involved in Clb5,6, Clb1,2 and Pds1 degradation
[$\mathrm{Mad2}_A$]	Concentration of active Mad2, a spindle checkpoint protein that sequesters (inactivates) Cdc20
[APCP]	Concentration of phosphorylated (active) APC, activates Cdc20 by complex formation
[$\mathrm{Cdh1}_A$]	Concentration of active Cdh1 degrading Clb1,2 and Polo kinase
[Net1dep]	Concentration of dephosphorylated (active) Net1, a stoichiometric inhibitor of Cdc14
[PPX]	Concentration of phosphatase that dephosphorylates Net1
[$\mathrm{Pds1}_T$]	Concentration of securin, Esp1's stoichiometric inhibitor
[Cdc15]	Concentration of a kinase, when complexed with Tem1, phosphorylates Net1
[Tem1]	Concentration of a protein, when complexed with Cdc15, phosphorylates Net1
[Polo_T]	Total concentration of Polo kinase that activates Tem1
[Polo_A]	Concentration of phosphorylated (active) Polo kinase that activates Tem1
[UDNA]	Set to 1 when [ORI]=1, and set back to 0 when cell divides.
[SPNALIGN]	Set to 1 when [SPN]=1, and set back to 0 when cell divides.
[ORIFLAG]	Set to 0 when [ORI]=1, and set back to 1 when [ORI]=0.

Each variable corresponds to a single ODE. Deterministic model variables, which represent the concentration values, are converted to stochastic model variables that represent the numbers of molecules. The conversion process is described in [8,11].
