# Supplementary material for: A Stochastic Model Correctly Predicts Changes in Budding Yeast Cell Cycle Dynamics upon Periodic Expression of CLN2
Source: PLoS One. 2014 May 9;9(5):e96726. doi: 10.1371/journal.pone.0096726 (PMC4016136; doi:10.1371/journal.pone.0096726)
Supplement: Table S2 — Parameters exclusive to the stochastic model. These parameters are only present in the stochastic model equations. (PDF) [file pone.0096726.s013.pdf]

Table S2. Parameters exclusive to the stochastic model

Name	Initial value	Optimized value	Range of LH sampling	Parameter description
k_{tr1}	0.22	0.3870	0.044–1.1	Translation rate of [Cln3]
k_{dm1}	0.7	2.9459	0.14–3.5	mRNA degradation rate of [Cln3]
m_{min1}	1	5	0.2–5	Minimum number of mRNA molecules for [Cln3]
m_{fluc1}	1	1	-	Magnitude of mRNA noise for [Cln3]
k_{tr2}	0.22	0.6166	0.044–1.1	Translation rate of [Bck2]
k_{dm2}	0.7	0.6033	0.14–3.5	mRNA degradation rate of [Bck2]
m_{min2}	4	17	0.8–20	Minimum number of mRNA molecules for [Bck2]
m_{fluc2}	1	1	-	Magnitude of mRNA noise for [Bck2]
k_{tr3}	0.22	0.0761	0.044–1.1	Translation rate of [Cln2]
k_{dm3}	0.7	2.9502	0.14–3.5	mRNA degradation rate of [Cln2]
m_{min3}	1	2	0.2–5	Minimum number of mRNA molecules for [Cln2]
m_{fluc3}	1	1	-	Magnitude of mRNA noise for [Cln2]
k_{tr4}	0.22	0.2024	0.044–1.1	Translation rate of [CKI_T]
k_{dm4}	0.7	1.4652	0.14–3.5	mRNA degradation rate of [CKI_T]
m_{min4}	4	1	0.8–20	Minimum number of mRNA molecules for [CKI_T]
m_{fluc4}	1	1	-	Magnitude of mRNA noise for [CKI_T]
k_{tr5}	0.22	0.6878	0.044–1.1	Translation rate of [$Clb5_T$]
k_{dm5}	0.7	0.1975	0.14-3.5	mRNA degradation rate of [$Clb5_T$]
m_{min5}	4	8	0.8–20	Minimum number of mRNA molecules for [$Clb5_T$]
m_{fluc5}	1	1	-	Magnitude of mRNA noise for [$Clb5_T$]
k_{tr6}	0.22	0.6974	0.044–1.1	Translation rate of [$Clb2_T$]
k_{dm6}	0.7	1.6668	0.14–3.5	mRNA degradation rate of [$Clb2_T$]
m_{min6}	4	15	0.8–20	Minimum number of mRNA molecules for [$Clb2_T$]
m_{fluc6}	1	1	-	Magnitude of mRNA noise for [$Clb2_T$]
k_{tr7}	0.22	0.8867	0.044–1.1	Translation rate of [$Swi5_T$]
k_{dm7}	0.7	2.4182	0.14–3.5	mRNA degradation rate of [$Swi5_T$]
m_{min7}	4	16	0.8–20	Minimum number of mRNA molecules for [$Swi5_T$]
m_{fluc7}	1	1	-	Magnitude of mRNA noise for [$Swi5_T$]
k_{tr8}	0.22	0.7344	0.044–1.1	Translation rate of [$CDC20_T$]
k_{dm8}	0.7	3.4411	0.14–3.5	mRNA degradation rate of [$CDC20_T$]
m_{min8}	4	6	0.8–20	Minimum number of mRNA molecules for [$CDC20_T$]
m_{fluc8}	1	1	-	Magnitude of mRNA noise for [$CDC20_T$]
k_{tr9}	0.22	0.6737	0.044–1.1	Translation rate of [$Pds1_T$]
k_{dm9}	0.7	1.2706	0.14–3.5	mRNA degradation rate of [$Pds1_T$]
m_{min9}	4	9	0.8–20	Minimum number of mRNA molecules for [$Pds1_T$]
m_{fluc9}	1	1	-	Magnitude of mRNA noise for [$Pds1_T$]
k_{tr10}	0.22	0.4258	0.044–1.1	Translation rate of [$Polo_T$]
k_{dm10}	0.7	0.1369	0.14–3.5	mRNA degradation rate of [$Polo_T$]
m_{min10}	4	5	0.8–20	Minimum number of mRNA molecules for [$Polo_T$]
m_{fluc10}	1	1	-	Magnitude of mRNA noise for [$Polo_T$]
c_{cln3}	10	9.0957	5–20	Characteristic concentration of Cln3
c_{bck2}	10	16.3317	5–20	Characteristic concentration of Bck2
c_{whi5}	22	21.8688	11–44	Characteristic concentration of Whi5
c_{sbf}	22	21.8688	11–44	Characteristic concentration of SBF
c_{cln2}	45	84.2260	22.5–90	Characteristic concentration of Cln2
c_{cki}	80	101.9969	40–160	Characteristic concentration of CKI
c_{clb5}	80	101.9969	40–160	Characteristic concentration of Clb5
c_{clb2}	80	101.9969	40–160	Characteristic concentration of Clb2
c_{swi5}	57.5	50.4561	28.75–115	Characteristic concentration of Swi5
c_{cdc20} (c_{mad2})	100	93.1338	50–200	Characteristic concentration of Cdc20 (Mad2)
c_{cdh1}	100	59.4664	50–200	Characteristic concentration of Cdh1
c_{apcp}	100	93.1338	50–200	Characteristic concentration of APCP
c_{cdc14}	14	20.2049	7–28	Characteristic concentration of Cdc14
c_{net1}	14	20.2049	7–28	Characteristic concentration of Net1
c_{ppx}	100	81.0649	50–200	Characteristic concentration of PPX
c_{pds1}	3.3	2.3993	1.65–6.6	Characteristic concentration of Pds1
c_{esp1}	3.3	2.3993	1.65–6.6	Characteristic concentration of Esp1
c_{cdc15}	8	8.7958	4–16	Characteristic concentration of Cdc15
c_{tem1}	8	8.7958	4–16	Characteristic concentration of Tem1
c_{men}	8	8.7958	4–16	Characteristic concentration of MEN
c_{polo}	100	155.2614	50–200	Characteristic concentration of Polo
c_{mcm1}	100	183.1687	50–200	Characteristic concentration of Mcm1
c_{vol}	28	28	-	Characteristic volume (in femtoliters)
