# Supplementary material for: A Stochastic Model Correctly Predicts Changes in Budding Yeast Cell Cycle Dynamics upon Periodic Expression of CLN2
Source: PLoS One. 2014 May 9;9(5):e96726. doi: 10.1371/journal.pone.0096726 (PMC4016136; doi:10.1371/journal.pone.0096726)
Supplement: Table S3 — List of 119 phenotypes. (PDF) [file pone.0096726.s014.pdf]

Table S3. List of 119 phenotypes

Phenotype #	Phenotype name	Viability	Changes in WT parameters
1	WT in glucose	Viable	NONE
2	WT in galactose	Viable	MDT=150; f =0.39;
3	*cln3*Δ	Viable	D_{n3}=0; [CLN3]=0;
4	*bck2*Δ	Viable	ks_{k2}=0; [BCK2]=0;
5	*cln3*Δ *bck2*Δ	Inviable	D_{n3}=0; [CLN3]=0; ks_{k2}=0;[BCK2]=0;
6	*cln3*Δ *bck2*Δ multicopy *CLN2*	Inviable	D_{n3}=0; [CLN3]=0; ks_{k2}=0; [BCK2]=0; ks_{n2bf}=ks_{n2bf}*2;
7	*cln3*Δ *bck2*Δ *sic*Δ	Inviable	D_{n3}=0; [CLN3]=0; ks_{k2}=0; [BCK2]=0; ks_{ki}=ks_{ki}*0.125; $ks_{ki,swi5}$=$ks_{ki,swi5}$*0.125; [CKIT]=0.2;
8	*cln3*Δ *bck2*Δ *whi5*Δ	Viable	D_{n3}=0; [CLN3]=0; ks_{k2}=0; [BCK2]=0; WHI5T=0; [WHI5A]=0;
9	*GAL-CLN3*	Viable	MDT=150; f =0.39; D_{n3}=D_{n3}*20;
10	Multicopy *BCK2*	Viable	ks_{k2}=ks_{k2}*17;
11	*cln1*Δ *cln2*Δ	Viable	ks_{n2}=0; ks_{n2bf}=0; [CLN2]=0;
12	*cln1*Δ *cln2*Δ *bck2*Δ	Viable	ks_{n2}=0; ks_{n2bf}=0; [CLN2]=0; ks_{k2}=0; [BCK2]=0;
13	*cln1*Δ *cln2*Δ *sic1*Δ	Viable	ks_{n2}=0; ks_{n2bf}=0; [CLN2]=0; ks_{ki}=ks_{ki}*0.125; $ks_{ki,swi5}$=$ks_{ki,swi5}$*0.125; [CKIT]=0.2;
14	*cln1*Δ *cln2*Δ *cki*Δ	Viable	ks_{n2}=0; ks_{n2bf}=0; [CLN2]=0; ks_{ki}=0; $ks_{ki,swi5}$=0; [CKIT]=0; [CKIP]=0;
15	*cln1*Δ *cln2*Δ *GAL-SIC1*	Inviable	ks_{n2}=0; ks_{n2bf}=0; [CLN2]=0; MDT=150; f =0.39; ks_{ki}=ks_{ki}*15;
16	*cln1*Δ *cln2*Δ *GAL-CLN2*	Viable	ks_{n2bf}=0; MDT=150; f =0.39; ks_{n2}=0.15;
17	*cln1*Δ *cln2*Δ *GAL-SIC1 GAL-CLN2*	Viable	ks_{n2bf}=0; MDT=150; f =0.39; ks_{n2}=0.15; ks_{ki}=ks_{ki}*15;
18	*cln1*Δ *cln2*Δ *cdh1*Δ	Viable	ks_{n2}=0; ks_{n2bf}=0; [CLN2]=0; CDH1T=0; [CDH1A]=0;
19	*cln1*Δ *cln2*Δ *cdh1*Δ *GAL-SIC1*	Inviable	ks_{n2}=0; ks_{n2bf}=0; [CLN2]=0; CDH1T=0; [CDH1A]=0; MDT=150; f =0.39; ks_{ki}=ks_{ki}*15;
20	*cln1*Δ *cln2*Δ *cdh1*Δ *GAL-CLN2*	Viable	ks_{n2bf}=0; CDH1T=0; [CDH1A]=0; MDT=150; f =0.39; ks_{n2}=0.15;
21	*cln1*Δ *cln2*Δ *cdh1*Δ *GAL-SIC1 GAL-CLN2*	Viable	ks_{n2bf}=0; CDH1T=0; [CDH1A]=0; MDT=150; f =0.39; ks_{n2}=0.15; ks_{ki}=ks_{ki}*15;
22	*cln1*Δ *cln2*Δ *cln3*Δ	Inviable	ks_{n2}=0; ks_{n2bf}=0; [CLN2]=0; D_{n3}=0; [CLN3]=0;
23	*cln1*Δ *cln2*Δ *cln3*Δ *GAL-CLN2*	Viable	ks_{n2bf}=0; D_{n3}=0; [CLN3]=0;

Continued on next page

Table S3 – *Continued from previous page*

Phenotype #	Phenotype name	Viability	Changes in WT parameters
			MDT=150; f=0.39; ks_{n2}=0.15;
24	*cln1Δ cln2Δ cln3Δ GAL-CLN3*	Viable	ks_{n2}=0; ks_{n2bf}=0; [CLN2]=0;<br>MDT=150; f =0.39; D_{n3}=D_{n3}*20;
25	*cln1Δ cln2Δ cln3Δ sicΔ*	Viable	ks_{n2}=0; ks_{n2bf}=0; [CLN2]=0;<br>D_{n3}=0; [CLN3]=0;<br>ks_{ki}=ks_{ki}*0.125; $ks_{ki,swi5}$=$ks_{ki,swi5}$*0.125;<br>[CKIT]=0.2;
26	*cln1Δ cln2Δ cln3Δ* multicopy *BCK2*	Viable	ks_{n2}=0; ks_{n2bf}=0; [CLN2]=0;<br>D_{n3}=0; [CLN3]=0;<br>ks_{k2}=ks_{k2}*17;
27	*cln1Δ cln2Δ cln3Δ bck2Δ GAL-CLN2*	Viable	ks_{n2bf}=0;<br>D_{n3}=0; [CLN3]=0;<br>ks_{k2}=0; [BCK2]=0;<br>MDT=150; f=0.39; ks_{n2}=0.15;
28	*cln1Δ cln2Δ cln3Δ* multicopy *CLB5*	Viable	ks_{n2}=0; ks_{n2bf}=0; [CLN2]=0;<br>D_{n3}=0; [CLN3]=0;<br>ks_{b5}=ks_{b5}=*5.33; $ks_{b5,bf}$=$ks_{b5,bf}$*5.33;
29	*cln1Δ cln2Δ cln3Δ GAL-CLB5*	Viable	ks_{n2}=0; ks_{n2bf}=0; [CLN2]=0;<br>D_{n3}=0; [CLN3]=0;<br>MDT=150; f =0.39; ks_{b5}=0.029;
30	*cln1Δ cln2Δ cln3Δ GAL-CLB2*	Inviable	ks_{n2}=0; ks_{n2bf}=0; [CLN2]=0;<br>D_{n3}=0; [CLN3]=0;<br>MDT=150; f =0.39; ks_{b2}=0.154;
31	*cln1Δ cln2Δ cln3Δ cdh1Δ*	Inviable	ks_{n2}=0; ks_{n2bf}=0; [CLN2]=0;<br>D_{n3}=0; [CLN3]=0;<br>CDH1T=0; [CDH1A]=0;
32	*cln2Δ cln3Δ apc-ts*	Inviable	ks_{n2}=0; ks_{n2bf}=0; [CLN2]=0;<br>D_{n3}=0; [CLN3]=0;<br>ks_{20}=0; $ks_{20,m1}$=0; CDH1T=0; [CDH1A]=0;
33	*cdh1Δ*	Viable	CDH1T=0; [CDH1A]=0;
34	*CDH1* constitutively active	Inviable	CDH1T=CDH1T*3; $ki_{h1,e}$=0;
35	*sic1Δ*	Viable	ks_{ki}=ks_{ki}*0.125; $ks_{ki,swi5}$=$ks_{ki,swi5}$*0.125;<br>[CKIT]=0.2;
36	*GAL-SIC1*	Viable	MDT=150; f=0.39; ks_{ki}=ks_{ki}*15;
37	*GAL-SIC1-dbΔ*	Inviable	MDT=150; f =0.39; ks_{ki}=ks_{ki}*15; kd_{kip}=0;
38	*sic1Δ cdc6Δ (ckiΔ)*	Viable	ks_{ki}=0; $ks_{ki,swi5}$=0; [CKIT]=0; [CKIP]=0;
39	*swi5Δ*	Viable	ks_{swi5}=0; $ks_{swi5,m1}$=0; [SWI5T]=0;
40	*sic1Δ cdc6Δ2-49 cdh1Δ*	Inviable	ks_{ki}=0; $ks_{ki,swi5}$=0; [CKIT]=0; [CKIP]=0;<br>CDH1T=0; [CDH1A]=0;
41	*swi5Δ cdh1Δ*	Inviable	ks_{swi5}=0; $ks_{swi5,m1}$=0; [SWI5T]=0;<br>CDH1T=0; [CDH1A]=0;
42	*swi5Δ cdh1Δ GAL-SIC1*	Viable	ks_{swi5}=0; $ks_{swi5,m1}$=0; [SWI5T]=0;<br>CDH1T=0; [CDH1A]=0;<br>MDT=150; f =0.39; ks_{ki}=ks_{ki}*15;
43	*clb5Δ clb6Δ*	Viable	ks_{b5}=0; $ks_{b5,bf}$=0; [CLB5T]=0;
44	*clb5Δ clb6Δ cln1Δ cln2Δ*	Inviable	ks_{b5}=0; $ks_{b5,bf}$=0; [CLB5T]=0;

Continued on next page

Table S3 – *Continued from previous page*

Phenotype #	Phenotype name	Viability	Changes in WT parameters
			ks_{n2}=0; ks_{n2bf}=0; [CLN2]=0;
45	*CLB5-db*Δ	Viable	$kd_{b5,20}$=0; $kd_{b5,20,i}$=0;
46	*CLB5-db*Δ *sic1*Δ	Inviable	$kd_{b5,20}$=0; $kd_{b5,20,i}$=0; ks_{ki}=ks_{ki}*0.125; $ks_{ki,swi5}$=$ks_{ki,swi5}$*0.125; [CKIT]=0.2;
47	*GAL-CLB5*	Viable	MDT=150; f=0.39; ks_{b5}=0.029;
48	*GAL-CLB5 sic1*Δ	Inviable	MDT=150; f =0.39; ks_{b5}=0.029; ks_{ki}=ks_{ki}*0.125; $ks_{ki,swi5}$=$ks_{ki,swi5}$*0.125; [CKIT]=0.2;
49	*GAL-CLB5 cdh1*Δ	Inviable	MDT=150; f =0.39; ks_{b5}=0.029; CDH1T=0; [CDH1A]=0;
50	*GAL-CLB5-db*Δ	Inviable	MDT=150; f =0.39; ks_{b5}=0.029; $kd_{b5,20}$=0; $kd_{b5,20,i}$=0;
51	*clb1*Δ *clb2*Δ	Inviable	ks_{b2}=0; $ks_{b2,m1}$=0; [CLB2T]=0;
52	*clb1*Δ *clb2*Δ *clb5*Δ *clb6*Δ	Inviable	ks_{b2}=0; $ks_{b2,m1}$=0; [CLB2T]=0; ks_{b5}=0; $ks_{b5,bf}$=0; [CLB5T]=0;
53	*GAL-CLB2*	Viable	MDT=150; f=0.39; ks_{b2}=0.154;
54	Multicopy *GAL-CLB2*	Inviable	MDT=150; f =0.39; ks_{b2}=ks_{b2}*242.42;
55	*GAL-CLB2 sic1*Δ	Inviable	MDT=150; f =0.39; ks_{b2}=0.154; ks_{ki}=ks_{ki}*0.125; $ks_{ki,swi5}$=$ks_{ki,swi5}$*0.125; [CKIT]=0.2;
56	*GAL-CLB2 cdh1*Δ	Inviable	MDT=150; f =0.39; ks_{b2}=0.154; CDH1T=0; [CDH1A]=0;
57	*GAL-CLB2 swi5*Δ	Inviable	MDT=150; f =0.39; ks_{b2}=0.154; ks_{swi5}=0; $ks_{swi5,m1}$=0; [SWI5T]=0;
58	*CLB2-db*Δ	Inviable	$kd_{b2,20}$=0; $kd_{b2,h1}$=$kd_{b2,h1}$*0.09; $kd_{b2,20,i}$=0;
59	*CLB2-db*Δ in galactose	Inviable	$kd_{b2,20}$=0; $kd_{b2,h1}$=$kd_{b2,h1}$*0.09; $kd_{b2,20,i}$=0; MDT=150; f =0.39;
60	*CLB2-db*Δ *GAL-SIC1*	Viable	$kd_{b2,20}$=0; $kd_{b2,h1}$=$kd_{b2,h1}$*0.09; $kd_{b2,20,i}$=0; MDT=150; f =0.39; ks_{ki}=ks_{ki}*15;
61	*CLB2-db*Δ multicopy *SIC1*	Viable	$kd_{b2,20}$=0; $kd_{b2,h1}$=$kd_{b2,h1}$*0.09; $kd_{b2,20,i}$=0; ks_{ki}=ks_{ki}*65; $ks_{ki,swi5}$=$ks_{ki,swi5}$*65;
62	*CLB2-db*Δ *clb5*Δ *clb6*Δ	Inviable	$kd_{b2,20}$=0; $kd_{b2,h1}$=$kd_{b2,h1}$*0.09; $kd_{b2,20,i}$=0; ks_{b5}=0; $ks_{b5,bf}$=0; [CLB5T]=0;
63	*CLB2-db*Δ *clb5*Δ *clb6*Δ in galactose	Viable	$kd_{b2,20}$=0; $kd_{b2,h1}$=$kd_{b2,h1}$*0.09; $kd_{b2,20,i}$=0; ks_{b5}=0; $ks_{b5,bf}$=0; [CLB5T]=0; MDT=150; f =0.39;
64	*GAL-CLB2-db*Δ	Inviable	$kd_{b2,20}$=0; $kd_{b2,h1}$=$kd_{b2,h1}$*0.09; $kd_{b2,20,i}$=0; MDT=150; f =0.39; ks_{b2}=0.154;
65	*CLB1 clb2*Δ	Viable	ks_{b2}=ks_{b2}*0.33; $ks_{b2,m1}$=$ks_{b2,m1}$*0.33;
66	*CLB1 clb2*Δ *cdh1*Δ	Inviable	ks_{b2}=ks_{b2}*0.33; $ks_{b2,m1}$=$ks_{b2,m1}$*0.33; CDH1T=0; [CDH1A]=0;
67	*CLB1 clb2*Δ *pds1*Δ	Inviable	ks_{b2}=ks_{b2}*0.33; $ks_{b2,m1}$=$ks_{b2,m1}$*0.33; ks_{pds}=0; [PDS1T]=0;
68	*cdc20-ts* (*cdc20*Δ)	Inviable	ks_{20}=0; $ks_{20,m1}$=0; [CDC20T]=0; [CDC20A-APCP]=0;
69	*clb5*Δ *clb6*Δ *cdc20*Δ	Inviable	ks_{b5}=0; $ks_{b5,bf}$=0; [CLB5T]=0; ks_{20}=0; $ks_{20,m1}$=0; [CDC20T]=0; [CDC20A-APCP]=0;

Continued on next page

Table S3 – *Continued from previous page*

Phenotype #	Phenotype name	Viability	Changes in WT parameters
70	*cdc20Δ pds1Δ*	Inviable	ks_{20}=0; $ks_{20,m1}$=0; [CDC20T]=0; [CDC20A-APCP]=0; ks_{pds}=0; [PDS1T]=0;
71	*clb5Δ clb6Δ cdc20Δ pds1Δ*	Viable	ks_{b5}=0; $ks_{b5,bf}$=0; [CLB5T]=0; ks_{20}=0; $ks_{20,m1}$=0; [CDC20T]=0; [CDC20A-APCP]=0; ks_{pds}=0; [PDS1T]=0;
72	*CLB5-dbΔ cdc20Δ pds1Δ*	Inviable	$kd_{b5,20}$=0; $kd_{b5,20,i}$=0; ks_{20}=0; $ks_{20,m1}$=0; [CDC20T]=0; [CDC20A-APCP]=0; ks_{pds}=0; [PDS1T]=0;
73	*CLB5-dbΔ pds1Δ*	Viable	$kd_{b5,20}$=0; $kd_{b5,20,i}$=0; ks_{pds}=0; [PDS1T]=0;
74	*GAL-CDC20*	Inviable	MDT=150; f=0.39; ks_{20}=10;
75	*GALL-CDC20 sic1Δ cdh1Δ*	Viable	MDT=150; f =0.39; ks_{20}=ks_{20}*1000; ks_{ki}=ks_{ki}*0.125; $ks_{ki,swi5}$=$ks_{ki,swi5}$*0.125; [CKIT]=0.2; CDH1T=0; [CDH1A]=0;
76	*GALL-CDC20 sic1Δ Cdc6Δ2-49 cdh1Δ*	Viable	MDT=150; f=0.39; ks_{20}=ks_{20}*1000; ks_{ki}=0; $ks_{ki,swi5}$=0; [CKIT]=0; [CKIP]=0; CDH1T=0; [CDH1A]=0;
77	*APC-A*	Viable	$ka_{cp,b2}$=0;
78	*APC-A sic1Δ*	Viable	$ka_{cp,b2}$=0; ks_{ki}=ks_{ki}*0.125; $ks_{ki,swi5}$=$ks_{ki,swi5}$*0.125; [CKIT]=0.2;
79	*APC-A sic1Δ cdc6Δ2-49*	Viable	$ka_{cp,b2}$=0; ks_{ki}=0; $ks_{ki,swi5}$=0; [CKIT]=0; [CKIP]=0;
80	*APC-A cdh1Δ*	Inviable	$ka_{cp,b2}$=0; CDH1T=0; [CDH1A]=0;
81	*APC-A cdh1Δ* in galactose	Inviable	$ka_{cp,b2}$=0; CDH1T=0; [CDH1A]=0; MDT=150; f =0.39;
82	*APC-A cdh1Δ GAL-SIC1*	Viable	$ka_{cp,b2}$=0; CDH1T=0; [CDH1A]=0; MDT=150; f =0.39; ks_{ki}=ks_{ki}*15;
83	*APC-A cdh1Δ* multicopy *SIC1*	Viable	$ka_{cp,b2}$=0; CDH1T=0; [CDH1A]=0; ks_{ki}=ks_{ki}*65; $ks_{ki,swi5}$=$ks_{ki,swi5}$*65;
84	*APC-A cdh1Δ* multicopy *CDC20*	Viable	$ka_{cp,b2}$=0; CDH1T=0; [CDH1A]=0; ks_{20}=ks_{20}*2; $ks_{20,m1}$=$ks_{20,m1}$*2;
85	*APC-A GAL-CLB2*	Inviable	$ka_{cp,b2}$=0; MDT=150; f=0.39; ks_{b2}=0.154;
86	*pds1Δ*	Viable	ks_{pds}=0; [PDS1T]=0;
87	*PDS1-dbΔ*	Inviable	$kd_{pds,20}$=0;
88	*GAL-PDS1-dbΔ*	Inviable	MDT=150; f =0.39; ks_{pds}=ks_{pds}*3.33; $kd_{pds,20}$=0;
89	*esp1-ts*	Inviable	ESP1T=0;
90	*GAL-PDS1-dbΔ esp1-ts*	Inviable	MDT=150; f =0.39; ks_{pds}=ks_{pds}*3.33; $kd_{pds,20}$=0; ESP1T=0;
91	*GAL-ESP1 cdc20-ts*	Inviable	MDT=150; f=0.39; ESP1T=ESP1T*2; ks_{20}=0; $ks_{20,m1}$=0; [CDC20T]=0; [CDC20A-APCP]=0;

Continued on next page

Table S3 – *Continued from previous page*

Phenotype #	Phenotype name	Viability	Changes in WT parameters
92	*ppx*Δ	Viable	PPXT=0; [PPX]=0;
93	*GAL-PPX*	Viable	MDT=150; f=0.39; PPXT=PPXT*2;
94	*tem1*Δ	Inviable	TEM1T=0; [TEM1]=0;
95	*net1-ts*	Viable	kas_{net}=kas_{net}*0.45;
96	*tem1*Δ *net1-ts*	Viable	TEM1T=0; [TEM1]=0; kas_{net}=kas_{net}*0.45;
97	*GAL-TEM1*	Viable	TEM1T=TEM1T*5;
98	*tem1-ts GAL-CDC15*	Viable	TEM1T=0; [TEM1]=0; MDT=150; f =0.39; CDC15T=CDC15T*10;
99	*tem1-ts* multicopy *CDC14*	Viable	TEM1T=0; [TEM1]=0; CDC14T=CDC14T*2;
100	Multicopy *CDC15*	Viable	CDC15T=CDC15T*20;
101	*tem1-ts* multicopy *CDC15*	Viable	TEM1T=0; [TEM1]=0; CDC15T=CDC15T*20;
102	*net1-ts cdc20-ts*	Inviable	kas_{net}=kas_{net}*0.45; ks_{20}=0; $ks_{20,m1}$=0; [CDC20T]=0; [CDC20A-APCP]=0;
103	*cdc15*Δ	Inviable	CDC15T=0;
104	*cdc15*Δ *net1-ts*	Viable	CDC15T=0; kas_{net}=kas_{net}*0.45;
105	*cdc15*Δ *net1-ts cdh1*Δ	Viable	CDC15T=0; kas_{net}=kas_{net}*0.45; CDH1T=0; [CDH1A]=0;
106	*cdc15-ts* multicopy *TEM1*	Inviable	CDC15T=0; TEM1T=TEM1T*5;
107	*cdc15-ts* multicopy *CDC14*	Viable	CDC15T=0; CDC14T=CDC14T*2;
108	*TAB6-1*	Viable	kas_{net}=kas_{net}*0.48;
109	*cdc15*Δ *TAB6-1*	Viable	CDC15T=0; kas_{net}=kas_{net}*0.48;
110	*TAB6-1 clb5*Δ *clb6*Δ	Inviable	kas_{net}=kas_{net}*0.48; ks_{b5}=0; $ks_{b5,bf}$=0; [CLB5T]=0;
111	*TAB6-1 CLB1 clb2*Δ	Viable	kas_{net}=kas_{net}*0.48; ks_{b2}=ks_{b2}*0.33; $ks_{b2,m1}$=$ks_{b2,m1}$*0.33;
112	*cdc14-ts*	Inviable	CDC14T=0;
113	*cdc14-ts sic1*Δ	Inviable	CDC14T=0; ks_{ki}=ks_{ki}*0.125; $ks_{ki,swi5}$=$ks_{ki,swi5}$*0.125; [CKIT]=0.2;
114	*cdc14-ts cdh1*Δ	Inviable	CDC14T=0; CDH1T=0; [CDH1A]=0;
115	*cdc14-ts GAL-SIC1*	Inviable	CDC14T=0; MDT=150; f =0.39; ks_{ki}=ks_{ki}*15;
116	*cdc14-ts GAL-CLN2*	Inviable	CDC14T=0; MDT=150; f =0.39; ks_{n2}=0.15;
117	*GAL-NET1*	Inviable	MDT=150; f =0.39; NET1T=NET1T*10.85;
118	*GAL-CDC14*	Inviable	MDT=150; f =0.39; CDC14T=CDC14T*7;
119	*GAL-NET1 GAL-CDC14*	Viable	MDT=150; f =0.39; CDC14T=CDC14T*7; NET1T=NET1T*10.85;
