# Supplementary material for: A Stochastic Model Correctly Predicts Changes in Budding Yeast Cell Cycle Dynamics upon Periodic Expression of CLN2
Source: PLoS One. 2014 May 9;9(5):e96726. doi: 10.1371/journal.pone.0096726 (PMC4016136; doi:10.1371/journal.pone.0096726)
Supplement: Table S4 — Nine phenotypes that are not captured by the deterministic model. Mismatches between the deterministic simulations and the experimental phenotypes. In the simulations, double-period oscillations are considered inviable even if the events are executed in the right order. (PDF) [file pone.0096726.s015.pdf]

Table S4. Nine phenotypes that are not captured by the deterministic model

Phenotype #	Experimental phenotype	Source of mismatch between the model and the experimental phenotype
15	Inviable	Viable in the simulations.
19	Inviable	Viable in the simulations.
38	Viable	Events executed in the correct order, but oscillations have double-period.
49	Inviable	Viable in the simulations.
66	Inviable	Viable in the simulations.
67	Inviable	Viable in the simulations (Esp1 is always active since *PDS1* is deleted).
78	Viable	Multiple ORI activation events per cycle.
79	Viable	Events executed in the correct order, but oscillations have double-period.
105	Viable	No events executed after first division

Mismatches between the deterministic simulations and the experimental phenotypes. In the simulations, double-period oscillations are considered inviable even if the events are executed in the right order.
