# Supplementary material for: A Stochastic Model Correctly Predicts Changes in Budding Yeast Cell Cycle Dynamics upon Periodic Expression of CLN2
Source: PLoS One. 2014 May 9;9(5):e96726. doi: 10.1371/journal.pone.0096726 (PMC4016136; doi:10.1371/journal.pone.0096726)
Supplement: Table S5 — Cell cycle events in the deterministic simulations. Each event corresponds to a concentration value and its specific threshold. Subscript stands for the current time step, whereas represents the previous time step in the simulations. Events 2 and 3 (also 4 and 5) can interchange order without the loss of viability. (PDF) [file pone.0096726.s016.pdf]

Table S5. Cell cycle events in the deterministic simulations

Event #	Event name	Related species	Event description
1	Cell division	Active Clb2	$[\text{Clb2}]_k \leq 0.2$ & $[\text{Clb2}]_{k-1} > 0.2$
2 (3)	ORI relicensing	ORI	$[\text{ORI}]_k = 0$
3 (2)	START transition	Active SBF	$[\text{SBFdep}]_k \geq SBF_T*0.5$ & $[\text{SBFdep}]_{k-1} < SBF_T*0.5$
4 (5)	Bud emergence	BUD	$[\text{BUD}]_k \geq 1$ & $[\text{BUD}]_{k-1} < 1$
5 (4)	ORI activation	ORI	$[\text{ORI}]_k \geq 1$ & $[\text{ORI}]_{k-1} < 1$
6	SPN alignment	SPN	$[\text{SPN}]_k \geq 1$ & $[\text{SPN}]_{k-1} < 1$
7	Esp1 activation	Active Esp1	$[\text{Esp1}]_k \geq 0.2$ & $[\text{Esp1}]_{k-1} < 0.2$

Each event corresponds to a concentration value and its specific threshold. Subscript k stands for the current time step, whereas $(k-1)$ represents the previous time step in the simulations. Events 2 and 3 (also 4 and 5) can interchange order without the loss of viability.
