# Supplementary material for: A Stochastic Model Correctly Predicts Changes in Budding Yeast Cell Cycle Dynamics upon Periodic Expression of CLN2
Source: PLoS One. 2014 May 9;9(5):e96726. doi: 10.1371/journal.pone.0096726 (PMC4016136; doi:10.1371/journal.pone.0096726)
Supplement: Table S6 — Parameters that are present both in deterministic and stochastic models. These parameter values capture 110 phenotypes (out of 119 phenotypes in Table S3) with deterministic simulations. Phenotypes that are listed in Table S4 are not captured. (PDF) [file pone.0096726.s017.pdf]

Table S6. Parameters that are present both in deterministic and stochastic models

Parameter	Description	Parameter	Description
γ_{Whi5}	Time scale for Whi5 activation	kd_{swi5}	Degradation rate of Swi5
γ_{ki}	CKI inactivation time scale	$ka_{swi5,14}$	Swi5 activation by Cdc14
γ_{cp}	APC activation time scale	$ki_{swi5,b2}$	Swi5 inactivation by Clb2
γ_{tem}	Tem1 activation time scale	$ka_{m1,b2}$	Mcm1 activation by Clb2
σ_{Whi5}	Sigmoidicity of Whi5 activation	ki_{m1}	Basal Mcm1 inactivation
σ_{net}	Sigmoidicity of Net1 activation	ks_{20}	Basal Cdc20 synthesis
ks_{n3}	Cln3 synthesis rate	$ks_{20,m1}$	Mcm1-dependent Cdc20 synthesis
J_{n3}	Michaelis-Menten constant	kd_{20}	Cdc20 degradation
D_{n3}	Dosage of CLN3 gene	ka_{20}	Basal Cdc20 activation
kd_{n3}	Cln3 degradation rate	$kd_{b5,20,i}$	Clb5 degradation by Cdc20
ks_{k2}	Bck2 synthesis rate	$kd_{clb2,20,i}$	Clb2 degradation by Cdc20
kd_{k2}	Bck2 degradation rate	$ki_{20,ori}$	Cdc20 inactivation by spindle checkpoint
kdp_{i5}	Basal Whi5 dephosphorylation	$ka_{cp,b2}$	APC phosphorylation by Clb2
kdp_{i514}	Whi5 dephosphorylation by Cdc14	ki_{cp}	APC inactivation
kp_{i5}	Basal Whi5 phosphorylation	ka_{h1}	Basal Cdh1 activation
kp_{i5n3}	Whi5 phosphorylation by Cln3	$ka_{h1,14}$	Cdh1 activation by Cdc14
kp_{i5k2}	Whi5 phosphorylation by Bck2	ki_{h1}	Basal inactivation of Cdh1
kp_{i5n2}	Whi5 phosphorylation by Cln2	$ki_{h1,e}$	Cdh1 inactivation rate
kp_{i5b5}	Whi5 phosphorylation by Clb5	$e_{h1,n3}$	Cdh1 inactivation by Cln3
kdp_{bf}	Basal SBF dephosphorylation	$e_{h1,n2}$	Cdh1 inactivation by Cln2
kp_{bfb2}	SBF phosphorylation by Clb2	$e_{h1,b5}$	Cdh1 inactivation by Clb5
ks_{n2}	Basal Cln2 synthesis rate	$e_{h1,b2}$	Cdh1 inactivation by Clb2
ks_{n2bf}	SBF-dependent Cln2 synthesis	kdp_{net}	Basal Net1 dephosphorylation
kd_{n2}	Cln2 degradation	$kdp_{net,14}$	Net1 dephosphorylation by Cdc14
ks_{ki}	Basal CKI synthesis rate	$kdp_{net,px}$	Net1 dephosphorylation by PPX
$ks_{ki,swi5}$	Swi5-dependent CKI synthesis	kp_{net}	Basal Net1 phosphorylation
kd_{ki}	Degradation rate of CKI	$kp_{net,b2}$	Net1 phosphorylation by Clb2
kd_{kip}	Degradation rate of CKIP	$kp_{net,en}$	Net1 phosphorylation by MEN
$kp_{ki,e}$	CKI phosphorylation rate	$kp_{net,15}$	Net1 phosphorylation by Cdc15
$e_{ki,n3}$	CKI phosphorylation by Cln3	ka_{px}	Basal PPX activation
$e_{ki,k2}$	CKI phosphorylation by Bck2	ki_{px}	Basal PPX inactivation
$e_{ki,n2}$	CKI phosphorylation by Cln2	$ki_{px,p1}$	PPX inactivation by Esp1
$e_{ki,b5}$	CKI phosphorylation by Clb5	ks_{pds}	Basal Pds1 synthesis
$e_{ki,b2}$	CKI phosphorylation by Clb2	kd_{pds}	Basal Pds1 degradation
kdp_{ki}	Basal dephosphorylation of CKI	$kd_{pds,20}$	Pds1 degradation by Cdc20A
$kdp_{ki,14}$	CKI dephosphorylation by Cdc14	$kd_{pds,20,i}$	Pds1 degradation by Cdc20
ks_{b5}	Basal Clb5 synthesis	ka_{15}	Basal Cdc15 activation
$ks_{b5,bf}$	SBF-dependent Clb5 synthesis	$ka_{15,14}$	Cdc15 activation by Cdc14
kd_{b5}	Basal Clb5 degradation	ki_{15}	Basal Cdc15 inactivation
$kd_{b5,20}$	Clb5 degradation by Cdc20A	$ki_{15,b2}$	Cdc15 inactivation by Clb2
ks_{b2}	Basal Clb2 synthesis	ka_{tem}	Basal Tem1 activation
$ks_{b2,m1}$	Mcm1-dependent Clb2 synthesis	$ka_{tem,lo}$	Tem1 activation by Polo
kd_{b2}	Basal Clb2 degradation	$ka_{tem,p1}$	Tem1 activation by Esp1
$kd_{b2,20}$	Clb2 degradation by Cdc20A	ki_{tem}	Basal inactivation of Tem1

Continued on next page

Table S6 – *Continued from previous page*

Parameter	Description	Parameter	Description
$kd_{b2,h1}$	Clb2 degradation by Cdh1A	$ki_{tem,px}$	Tem1 inactivation by PPX
$ks_{bud,e}$	Time scale for BUD synthesis	ks_{lo}	Basal Polo synthesis
$e_{bud,n3}$	Cln3 activation of BUD	$ks_{lo,m1}$	Mcm1-dependent synthesis of Polo
$e_{bud,n2}$	Cln2 activation of BUD	kd_{lo}	Basal Polo degradation
$e_{bud,b5}$	Clb5 activation of BUD	$kd_{lo,h1}$	Polo degradation by Cdh1
$e_{bud,b2}$	Clb2 activation of BUD	ka_{lo}	Basal Polo activation
kd_{bud}	BUD degradation	$ka_{lo,b2}$	Polo activation by Clb2
ks_{spn}	SPN synthesis	ki_{lo}	Basal Polo inactivation
kd_{spn}	SPN degradation	kas_{net}	Efficiency of Cdc14-Net1 complex (RENT) formation
J_{spn}	SPN synthesis threshold	f	Fraction of mass retained by daughter at division
$ks_{ori,e}$	Time scale for ORI synthesis	MDT	Mass doubling time
$e_{ori,b5}$	Clb5 activation of ORI	$Whi5_T$	Total Whi5
$e_{ori,b2}$	Clb2 activation of ORI	SBF_T	Total SBF
kd_{ori}	Degradation of ORI	$Mcm1_T$	Total Mcm1
ks_{swi5}	Basal Swi5 synthesis	APC_T	Total APC
$ks_{swi5,m1}$	Mcm1-dependent Swi5 synthesis	$Cdh1_T$	Total Cdh1
σ_{SBF}	Sigmoidicity of SBF activation	$Net1_T$	Total Net1
σ_{CKI}	Sigmoidicity of CKI activation	$Cdc14_T$	Total Cdc14
σ_{Swi5}	Sigmoidicity of Swi5 activation	PPX_T	Total PPX
σ_{Cdh1}	Sigmoidicity of Cdh1 activation	$Esp1_T$	Total Esp1
σ_{Mcm1}	Sigmoidicity of Mcm1 activation	$Cdc15_T$	Total Cdc15
σ_{APC}	Sigmoidicity of APC activation	$Tem1_T$	Total Tem1
σ_{Mad2}	Sigmoidicity of Mad2 activation	$Mad2_T$	Total Mad2
σ_{PPX}	Sigmoidicity of PPX activation	γ_{SBF}	Time scale for SBF activation
σ_{Polo}	Sigmoidicity of Polo activation	γ_{Cdh1}	Time scale for Cdh1 activation
σ_{Tem1}	Sigmoidicity of Tem1 activation	γ_{Mad2}	Time scale for Mad2 activation
σ_{Cdc15}	Sigmoidicity of Cdc15 activation	γ_{PPX}	Time scale for Cdc15 activation
K_{EZ}	Cell divides when [Clb2] drops below K_{EZ}	γ_{Net1}	Time scale for Net1 activation
K_{EZ2}	[ORI] and [SPN] are reset to 0 when [Clb2]+[Clb5] drops below K_{EZ2}	γ_{Polo}	Time scale for Polo activation
		γ_{Cdc15}	Time scale for Cdc15 activation

These parameter values capture 110 phenotypes (out of 119 phenotypes in Table S3) with deterministic simulations. Phenotypes that are listed in Table S4 are not captured.
