# Supplementary material for: A Stochastic Model Correctly Predicts Changes in Budding Yeast Cell Cycle Dynamics upon Periodic Expression of CLN2
Source: PLoS One. 2014 May 9;9(5):e96726. doi: 10.1371/journal.pone.0096726 (PMC4016136; doi:10.1371/journal.pone.0096726)
Supplement: Table S7 — Values of parameters that are present both in deterministic and stochastic models. In the stochastic simulations, the daughter cell mass retained after each division () is the remainder of the total mass after the mother fraction is randomly assigned from a normal distribution with 0.58 mean and 0.029 standard deviation. (PDF) [file pone.0096726.s018.pdf]

Table S7. Values of parameters that are present both in deterministic and stochastic models

Parameter	Value	Parameter	Value
γ_{Whi5}	1	kd_{swi5}	0.08
γ_{ki}	10	$ka_{swi5,14}$	2
γ_{cp}	0.8	$ki_{swi5,b2}$	8.5
γ_{tem}	0.1	$ka_{m1,b2}$	10
σ_{Whi5}	10	ki_{m1}	3
σ_{net}	8	ks_{20}	0.006
ks_{n3}	0.22	$ks_{20,m1}$	0.6
J_{n3}	6	kd_{20}	0.3
D_{n3}	1	ka_{20}	0.4
kd_{n3}	0.2	$kd_{b5,20,i}$	0.0145
ks_{k2}	0.0135	$kd_{clb2,20,i}$	0.037
kd_{k2}	0.25	$ki_{20,mad}$	20
kdp_{i5}	1	$ka_{cp,b2}$	1.3
kdp_{i514}	0.5	ki_{cp}	1
kp_{i5}	0	ka_{h1}	1
kp_{i5n3}	3	$ka_{h1,14}$	4.3
kp_{i5k2}	4	ki_{h1}	0
kp_{i5n2}	2	$ki_{h1,e}$	1
kp_{i5b5}	0	$e_{h1,n3}$	0.25
kdp_{bf}	1	$e_{h1,n2}$	0.7
kp_{bfb2}	5	$e_{h1,b5}$	9
ks_{n2}	0	$e_{h1,b2}$	5.6
ks_{n2bf}	0.24	kdp_{net}	0.1
kd_{n2}	0.12	$kdp_{net,14}$	0.1
ks_{ki}	0.0120	$kdp_{net,px}$	3
$ks_{ki,swi5}$	0.12	kp_{net}	0
kd_{ki}	0.005	$kp_{net,b2}$	0.25
kd_{kip}	2.5	$kp_{net,en}$	1
$kp_{ki,e}$	1	$kp_{net,15}$	0.03
$e_{ki,n3}$	1.05	ka_{px}	1
$e_{ki,k2}$	0.1	ki_{px}	0
$e_{ki,n2}$	0.4	$ki_{px,p1}$	3
$e_{ki,b5}$	2	ks_{pds}	0.03
$e_{ki,b2}$	4	kd_{pds}	0.05
kdp_{ki}	1	$kd_{pds,20}$	3
$kdp_{ki,14}$	1.8	$kd_{pds,20,i}$	0.3
ks_{b5}	0.0003	ka_{15}	0
$ks_{b5,bf}$	0.0145	$ka_{15,14}$	15
kd_{b5}	0.012	ki_{15}	1
$kd_{b5,20}$	0.1	$ki_{15,b2}$	0.5
ks_{b2}	0.0033	ka_{tem}	0
$ks_{b2,m1}$	0.022	$ka_{tem,lo}$	4.25
kd_{b2}	0.003	$ka_{tem,p1}$	0

Continued on next page

Table S7 – *Continued from previous page*

Parameter	Value	Parameter	Value
$kd_{b2,20}$	0.14	ki_{tem}	1
$kd_{b2,h1}$	0.6	$ki_{tem,px}$	22
$ks_{bud,e}$	0.3	ks_{lo}	0
$e_{bud,n3}$	0.05	$ks_{lo,m1}$	0.03
$e_{bud,n2}$	0.5	kd_{lo}	0.01
$e_{bud,b5}$	0.5	$kd_{lo,h1}$	0.01
$e_{bud,b2}$	0	ka_{lo}	0
kd_{bud}	0.01	$ka_{lo,b2}$	10
ks_{spn}	0.1	ki_{lo}	1
kd_{spn}	0.06	kas_{net}	1
J_{spn}	0.13	f	0.42
$ks_{ori,e}$	2	MDT	84 (or 100)
$e_{ori,b5}$	0.4	$Whi5_T$	2.5
$e_{ori,b2}$	0.7	SBF_T	1
kd_{ori}	0.06	$Mcm1_T$	1
ks_{swi5}	0.0050	APC_T	25
$ks_{swi5,m1}$	0.08	$Cdh1_T$	1
σ_{SBF}	10	$Net1_T$	3.55
σ_{CKI}	10	$Cdc14_T$	2
σ_{Swi5}	10	PPX_T	1
σ_{Cdh1}	10	$Esp1_T$	0.5
σ_{Mcm1}	10	Cdc15$_T$	1
σ_{APC}	10	$Tem1_T$	2
σ_{Mad2}	10	$Mad2_T$	25
σ_{PPX}	10	γ_{SBF}	1
σ_{Polo}	10	γ_{Cdh1}	1
σ_{Tem1}	10	γ_{Mad2}	1
σ_{Cdc15}	10	γ_{PPX}	1
K_{EZ}	0.2	γ_{Net1}	1
K_{EZ2}	0.2	γ_{Polo}	1
		γ_{Cdc15}	1

In the stochastic simulations, the daughter cell mass retained after each division (f) is the remainder of the total mass after the mother fraction is randomly assigned from a normal distribution with 0.58 mean and 0.029 standard deviation.
