# Supplementary material for: A Stochastic Model Correctly Predicts Changes in Budding Yeast Cell Cycle Dynamics upon Periodic Expression of CLN2
Source: PLoS One. 2014 May 9;9(5):e96726. doi: 10.1371/journal.pone.0096726 (PMC4016136; doi:10.1371/journal.pone.0096726)
Supplement: Table S8 — Protein abundances. Abundances of some of the key cell cycle proteins: experimental vs. model values. Abundance data is collected in the simulations as described in [8] on page 59. Simulation statistics (mean standard deviation) are computed from 15 realizations. In each realization, twenty pedigrees are generated independently. Each pedigree of cells is initiated by a single daughter (D) or mother (M) cell. In some cases, experimental abundance data from diploid cells are halved for approximating the abundances in haploid cells. (PDF) [file pone.0096726.s019.pdf]

Table S8. Protein abundances

Protein	Experiment [15,16] (per haploid cell)	Simulation before parameter optimization	Simulation after parameter optimization
Whi5	1440	1683 ± 10	1712 ± 14
Net1	1590	1521 ± 9	2246 ± 18
Cdc15	238	245 ± 1	275 ± 2
Tem1	573	490 ± 3	551 ± 4
Cln3	108	81 ± 2	76 ± 1
Cln2	1589	1473 ± 33	2800 ± 65
Clb5	438	964 ± 15	1239 ± 25
Clb2	639	389 ± 16	516 ± 25
CKI	768	532 ± 16	668 ± 31

Abundances of some of the key cell cycle proteins: experimental vs. model values. Abundance data is collected in the simulations as described in [8] on page 59. Simulation statistics (mean $\pm$ standard deviation) are computed from 15 realizations. In each realization, twenty pedigrees are generated independently. Each pedigree of cells is initiated by a single daughter (D) or mother (M) cell. In some cases, experimental abundance data from diploid cells are halved for approximating the abundances in haploid cells.
