# Supplementary material for: A Stochastic Model Correctly Predicts Changes in Budding Yeast Cell Cycle Dynamics upon Periodic Expression of CLN2
Source: PLoS One. 2014 May 9;9(5):e96726. doi: 10.1371/journal.pone.0096726 (PMC4016136; doi:10.1371/journal.pone.0096726)
Supplement: Table S9 — The correct order of the cell cycle events in the stochastic simulations are enforced by checking for the presences of execution errors. Event checking is based on the concentration thresholds (for ORI, SBF, Esp1, SPN, BUD, and Clb2) listed in Table S5. The numbers of molecules recorded in the stochastic simulations are converted to concentrations for this purpose. Once an execution error in a cycle is detected, the pedigree cannot continue from the particular cell that have executed the event incorrectly (no progeny can be born from this cell). In that case, the cycle is recorded as a failed cycle. (PDF) [file pone.0096726.s020.pdf]

**Table S9. The correct order of the cell cycle events in the stochastic simulations are enforced by checking for the presences of execution errors.**

| Execution error # | Execution error |
|---|---|
| 1 | Bud emergence executed before START transition |
| 2 | Cell division executed before START transition |
| 3 | Cell division executed before bud emergence |
| 4 | SPN alignment executed before bud emergence |
| 5 | START transition not executed before ORI activation |
| 6 | Cell division executed before ORI activation |
| 7 | ORI not relicensed before ORI activation |
| 8 | SPN is aligned before ORI activation |
| 9 | START transition not executed before SPN alignment |
| 10 | Bud does not emerge before SPN alignment |
| 11 | ORI is not activated before SPN alignment |
| 12 | START transition not executed before cell division |
| 13 | Bud does not emerge before cell division |
| 14 | ORI activation is not executed before cell division |
| 15 | SPN alignment is not executed before cell division |
| 16 | Esp1 is not activated before cell division |
| 17 | Esp1 is activated before SPN alignment |
| Additional error | $V > 20$ at any time during the simulation<br>Wild type $V$ at division= 2.7 arbitrary units<br>with deterministic model (mass doubling time= 100 min) |

Event checking is based on the concentration thresholds (for ORI, SBF, Esp1, SPN, BUD, and Clb2) listed in Table S5. The numbers of molecules recorded in the stochastic simulations are converted to concentrations for this purpose. Once an execution error in a cycle is detected, the pedigree cannot continue from the particular cell that have executed the event incorrectly (no progeny can be born from this cell). In that case, the cycle is recorded as a failed cycle.
