# Supplementary material for: A Stochastic Model Correctly Predicts Changes in Budding Yeast Cell Cycle Dynamics upon Periodic Expression of CLN2
Source: PLoS One. 2014 May 9;9(5):e96726. doi: 10.1371/journal.pone.0096726 (PMC4016136; doi:10.1371/journal.pone.0096726)
Supplement: Table S10 — Initial condition set. Initial concentration values in the deterministic simulations and the initial numbers of molecules in the stochastic simulations (in parentheses). The conversion of concentrations to numbers of molecules is explained in [8], [11]. In cln3 stochastic simulations, the initial number of Cln3 molecules is set to zero, other values are shown as above. For MET3-CLN2 cln3 stochastic simulations (with forced CLN2 expression), the initial numbers of molecules come from the end points of 2000 min cln3 simulations in order to mimic the experimental conditions in [7]. (PDF) [file pone.0096726.s021.pdf]

Table S10. Initial condition set

Variable	Value
V_0	1.1350
$[\text{Cln3}]_0$	0.3115 (54)
$[\text{Bck2}]_0$	0.1283 (40)
$[\text{WHI5dep}]_0$	1.3762 (575)
$[\text{SBFdep}]_0$	0.4945 (206)
$[\text{Cln2}]_0$	0.1237 (199)
$[\text{CKI}_T]_0$	0.1596 (311)
$[\text{CKI}_P]_0$	0 (0)
$[\text{Clb5}_T]_0$	0.1848 (360)
$[\text{Clb2}_T]_0$	0.1666 (325)
$[\text{BUD}]_0$	0.0305
$[\text{ORI}]_0$	0.0104
$[\text{SPN}]_0$	0.0404
$[\text{Swi5}_T]_0$	0.6532 (630)
$[\text{CDC20}_T]_0$	1.3909 (2478)
$[\text{Mad2}_A]_0$	0.4497 (801)
$[\text{APCP}]_0$	0.1627 (289)
$[\text{Cdh1}_A]_0$	0.9370 (1066)
$[\text{Net1dep}]_0$	0.1785 (69)
$[\text{PPX}]_0$	0.0117 (18)
$[\text{Pds1}_T]_0$	0.0252 (1)
$[\text{Cdc15}]_0$	0.9756 (164)
$[\text{Tem1}]_0$	0.7519 (126)
$[\text{Polo}_T]_0$	0.4182 (1242)
$[\text{Polo}_A]_0$	0.3726 (1106)
$[\text{UDNA}]_0$	0
$[\text{SPNALIGN}]_0$	0
$[\text{ORIFLAG}]_0$	1

Initial concentration values in the deterministic simulations and the initial numbers of molecules in the stochastic simulations (in parentheses). The conversion of concentrations to numbers of molecules is explained in [8,11]. In *cln3* stochastic simulations, the initial number of Cln3 molecules is set to zero, other values are shown as above. For *MET3-CLN2 cln3* stochastic simulations (with forced *CLN2* expression), the initial numbers of molecules come from the end points of 2000 min *cln3* simulations in order to mimic the experimental conditions in [7].
