# Supplementary material for: A Stochastic Model Correctly Predicts Changes in Budding Yeast Cell Cycle Dynamics upon Periodic Expression of CLN2
Source: PLoS One. 2014 May 9;9(5):e96726. doi: 10.1371/journal.pone.0096726 (PMC4016136; doi:10.1371/journal.pone.0096726)
Supplement: Table S11 — Start/end time points (min) of the forced CLN2 expression pulses. is the period of forced CLN2 expression (in minutes). Start and end points of the pulses (in minutes) with MET3 promoter delay decide the actual time intervals during which CLN2 expression pulses are administered into the system during the simulations. Start time points without promoter delay are used to compute the time between the closest starting point and budding in the current cycle () and the subsequent cycle (), as it was done in the experimental study [7]. During cln3 (control) simulations, forced CLN2 expression is not present. In these simulations, and are computed as the differences between the time points of budding in the current and subsequent cycles with the closest preceding pulse start points without promoter delay, respectively (Figure S2 and Figures 4A and 4C). This procedure was also followed in the experimental study [7]. With or without forced CLN2 expression, the closest pulse start time that is selected to compute (or ) is before the time point of budding [7] (i.e., start point precedes budding). (PDF) [file pone.0096726.s022.pdf]

Table S11. Start/end time points (min) of the forced *CLN2* expression pulses

With delay?	No	No	Yes	Yes	No	No	Yes	Yes	No	No	Yes	Yes
Pulse #	Start τ =90	End τ =90	Start τ =90	End τ =90	Start τ =78	End τ =78	Start τ =78	End τ =78	Start τ =69	End τ =69	Start τ =69	End τ =69
1	0	20	16.8	36.8	0	20	16.8	36.8	0	20	16.8	36.8
2	90	110	106.8	126.8	78	98	94.8	114.8	69	89	85.8	105.8
3	180	200	196.8	216.8	156	176	172.8	192.8	138	158	154.8	174.8
4	270	290	286.8	306.8	234	254	250.8	270.8	207	227	223.8	243.8
5	360	380	376.8	396.8	312	332	328.8	348.8	276	296	292.8	312.8
6	450	470	466.8	486.8	390	410	406.8	426.8	345	365	361.8	381.8
7	540	560	556.8	576.8	468	488	484.8	504.8	414	434	430.8	450.8
8	630	650	646.8	666.8	546	566	562.8	582.8	483	503	499.8	519.8
9	-	-	-	-	624	644	640.8	660.8	552	572	568.8	588.8
10	-	-	-	-	-	-	-	-	621	641	637.8	657.8
11	-	-	-	-	-	-	-	-	690	710	706.8	726.8

τ is the period of forced *CLN2* expression (in minutes). Start and end points of the pulses (in minutes) with *MET3* promoter delay decide the actual time intervals during which *CLN2* expression pulses are administered into the system during the simulations. Start time points without promoter delay are used to compute the time between the closest starting point and budding in the current cycle (t_n) and the subsequent cycle (t_{n+1}), as it was done in the experimental study [7]. During *cln3* (control) simulations, forced *CLN2* expression is not present. In these simulations, t_n and t_{n+1} are computed as the differences between the time points of budding in the current and subsequent cycles with the closest preceding pulse start points without promoter delay, respectively (Figure S2 and Figures 4A and 4C). This procedure was also followed in the experimental study [7]. With or without forced *CLN2* expression, the closest pulse start time that is selected to compute t_n (or t_{n+1}) is before the time point of budding [7] (i.e., start point precedes budding).
