# Supplementary material for: A Stochastic Model Correctly Predicts Changes in Budding Yeast Cell Cycle Dynamics upon Periodic Expression of CLN2
Source: PLoS One. 2014 May 9;9(5):e96726. doi: 10.1371/journal.pone.0096726 (PMC4016136; doi:10.1371/journal.pone.0096726)
Supplement: Table S12 — Summary of the statistics regarding the G1 duration bimodality observed in Figures S9 and S10. Statistics of the small-born cells exhibiting bimodality of the G1 duration with 78 and 69 min periods of forced CLN2 expression. (PDF) [file pone.0096726.s023.pdf]

**Table S12. Summary of the statistics regarding the G1 duration bimodality observed in Figures S9 and S10**

| Pulse period ($\tau$) | 78 min | 78 min | 69 min | 69 min |
|---|---|---|---|---|
| Description of the cell group | Small-born daughters with long G1 duration (orange box:Figure S9) | Small-born mothers with long G1 duration (orange box:Figure S9) | Small-born daughters with long G1 duration (orange box:Figure S10) | Small-born mothers with long G1 duration (orange box:Figure S10) |
| # cells in the group | 23 | 19 | 349 | 292 |
| # parents of the cells in the group that are mothers (% in green box of Figure S9/S10) | 2 (100%) | 0 - | 67 (69%) | 21 (100%) |
| # parents of the cells in the group that are daughters (% in green box of Figure S9/S10) | 21 (100%) | 19 (100%) | 282 (96%) | 271 (96%) |
| # of parent cells common to small-born daughters and mothers with long G1 duration | 17 of 23 | 17 of 19 | 262 of 349 | 262 of 292 |
| # all parents in the pedigree that are mothers | 2305 | | 2853 | |
| # all parents in the pedigree that are daughters | 1933 | | 2468 | |
| Average budded period length among parents of cells in the group | 75.22 min (std=6.96 min) | 77.99 min (std=4.20 min) | 70.81 min (std=6.80 min) | 72.54 min (std=6.01 min) |
| Average budded period length of all parent cells in the pedigree that are daughters | 48.93 min (std=8.69 min) | | 58.45 min (std=10.82 min) | |
| Average budded period length of all parent cells in the pedigree that are mothers | 45.69 min (std=4.60 min) | | 50.18 min (std=6.53 min) | |

Statistics of the small-born cells exhibiting bimodality of the G1 duration with 78 and 69 min periods of forced *CLN2* expression.
