# Supplementary material for: A Stochastic Model Correctly Predicts Changes in Budding Yeast Cell Cycle Dynamics upon Periodic Expression of CLN2
Source: PLoS One. 2014 May 9;9(5):e96726. doi: 10.1371/journal.pone.0096726 (PMC4016136; doi:10.1371/journal.pone.0096726)
Supplement: Table S13 — Synchronization levels with different MET3pr strengths (forcing period of 78 min). Pulse duration is 20 min. is the fraction of time points (between 300–700 min in the simulations) at which more than 95% or less than 5% of the cells are budded. The simulation statistics (mean standard deviation) are computed from 15 independent realizations per promoter strength. In each realization, a budding index trajectory is generated from a pedigree. Each trajectory starts from a single cell and the number of cells within the pedigree increases exponentially due to cell division. The number of the failed cycles (due to event execution errors listed in Table S9) normalized by the number of complete cycles is the cycle failure ratio. (PDF) [file pone.0096726.s024.pdf]

Table S13. Synchronization levels with different *MET3pr* strengths (forcing period of 78 min)

MET3pr strength	f_{synch}	Cycle failure ratio
0.5	0.02 ± 0.04	0.00 ± 0.00
1	0.25 ± 0.06	0.00 ± 0.00
4	0.46 ± 0.08	0.04 ± 0.01

Pulse duration is 20 min. f_{synch} is the fraction of time points (between 300–700 min in the simulations) at which more than 95% or less than 5% of the cells are budded. The simulation statistics (mean ± standard deviation) are computed from 15 independent realizations per promoter strength. In each realization, a budding index trajectory is generated from a pedigree. Each trajectory starts from a single cell and the number of cells within the pedigree increases exponentially due to cell division. The number of the failed cycles (due to event execution errors listed in Table S9) normalized by the number of complete cycles is the cycle failure ratio.
