# Supplementary material for: A Stochastic Model Correctly Predicts Changes in Budding Yeast Cell Cycle Dynamics upon Periodic Expression of CLN2
Source: PLoS One. 2014 May 9;9(5):e96726. doi: 10.1371/journal.pone.0096726 (PMC4016136; doi:10.1371/journal.pone.0096726)
Supplement: Text S1 — Identification of the model's locking time window. (PDF) [file pone.0096726.s026.pdf]

Text S1

Identification of the model's locking time window

The extent of locking for mothers or daughters is the fraction of cells that fall within the locking time window around the line $t_n = t_{n+1}$ on each return map. A locking time window of 20 min–42 min (for both t_n and t_{n+1}) was used in [7]. Since the extent of locking can be sensitive to the exact position of the locking time window, we sample 10000 potential time windows using LH sampling (described in the Supplementary Text of [9]) . These sample time windows have lower bounds ranging from 20 to 42 min and upper bounds ranging from 21 to 64 min. In other words, we allow the upper bound of the locking range to exceed the experimental value (42 min) in order to find the time window that gives the best agreement between the simulated and experimentally observed locking extents. About half our samples (5445 of 10000) are 10 minute or longer time windows. For each of these 5445 time windows, we compute the mean absolute error as $\sum_{k=1}^{6} \left| x_k - e_k \right| /6$, where x_k and e_k are the kth locked fraction values in simulations and experiments, respectively. Here, we have six data points: three pulse periods (90, 78, and 69 min), each with a daughter and mother locked fraction. Figure S3 shows the mean absolute error among the 5445 sampled time windows that are 10–22 minutes long. The optimal time window with the minimum error is 20.87–34.76 min (marked with the red square) with a mean absolute error of 0.10 (10%). According to Figure S3, as the lower (upper) bound of the locking time window is increased beyond 28 (39) min, the error is exceedingly high (> 0.33). This shows that our model performs better in the experimentally relevant part of the locking regime. Also, our optimal time window (20.87–34.76 min) is within the experimental range (20–42 min) even though we sample beyond this range up to 64 min. However, when we enforce 20–42 min as the locking window in our simulations, the error nearly doubles (0.18) pointing out the sensitivity of the locked fractions to the exact position of the locking window.
