# Supplementary material for: A Stochastic Model Correctly Predicts Changes in Budding Yeast Cell Cycle Dynamics upon Periodic Expression of CLN2
Source: PLoS One. 2014 May 9;9(5):e96726. doi: 10.1371/journal.pone.0096726 (PMC4016136; doi:10.1371/journal.pone.0096726)
Supplement: Text S2 — Model predictions with complex MET3 promoter dynamics. (PDF) [file pone.0096726.s027.pdf]

Text S2

Model predictions with complex *MET3* promoter dynamics

We assess the robustness of the model predictions regarding the fractions of locked mothers and daughters (shown in Figure 6) with respect to promoter dynamics. For this assessment, we perform simulations in which periodic *CLN2* expression from the *MET3* promoter is gradually turned on and gradually turned off instead of the simpler promoter dynamics based on a step function (immediate turn on and turn off). This is a more realistic way to simulate the forced *CLN2* expression from the *MET3* promoter based on the experimental characterization of *MET3* in [17]. Taking into account the amount of time needed for complete shut-off of the *MET3* promoter (10 min) following the media shift (no methionine to methionine) from [17], we increase the duration of actual forced *CLN2* expression from 20 min to 30 min. In other words, even though the duration of the methionine absence (*MET3* is active without methionine in the media) is 20 min, the actual duration of the *CLN2* expression pulse has a longer duration (also observed experimentally in [17]). We modify the function describing the promoter activity with respect to time so that the promoter would reach its maximum activity gradually (maximum activity is reached at the midpoint of the 30 min period during which *CLN2* is expressed from the *MET3* promoter). The experimentally measured lag of 16.8 min [17] is also taken into account as before. Hence, when *MET3* is removed from the system in the simulations, no *CLN2* is expressed from the *MET3-CLN2* construct for 16.8 min, and this is followed by gradual increase of *MET3* activity for 15 min until the maximum promoter activity is reached. In the following 15 min of the *MET3* activity, forced *CLN2* expression declines to "zero" gradually. In order to mathematically represent the promoter activity with the dynamics we just described (during the 30 min period of *CLN2* activity), the following parabolic function is used in the simulations:

$$MET3pr = \big(-2 \times max(MET3pr) \times (mod(t,\tau) - 31.8)^2\big)/225 + \big(max(MET3pr)\big)^2.$$

Here, $max(MET3pr)$ represents the maximum promoter activity, t is the time point in the simulations (in minutes), τ is the forcing period, and $mod(t,\tau)$ is the remainder of the division of t by τ. This function is active during the simulations only when the following condition is satisfied: $16.8 \leq mod(t,\tau) \leq 46.8$. For simulation time points at which this condition is not satisfied, there is no *CLN2* expression from the *MET3* promoter. For the simple promoter dynamics (step function), instead of the parabolic function, the promoter is activity is simply *max(MET3pr)* when $16.8 \leq mod(t,\tau) \leq 46.8$.

Figure S4 shows the comparison of the *MET3-CLN2* activity with respect to time with the simple promoter dynamics (periodic step function) and the complex promoter dynamics (periodic parabolic function). We note that, in order to compensate for the gradual *MET3* promoter turn on as opposed to instant turn on, the maximum promoter activity with complex promoter dynamics is the double of the maximum activity with step function. However, even with this doubling, the *MET3* promoter activity is still within the experimentally reported physiological range of the *MET3* promoter strength [17] with respect to the *CLN2* expression from the native *CLN2* copy.

Figure S5 shows that the model predictions for the fraction of locked cells with six different forcing periods are approximately the same with and without the complex promoter dynamics. In other words, these predictions are robust to the level of detail in the simulated promoter dynamics. We also note that the observed variabilities of these predictions is low (standard deviation less than 15% of the mean) in both cases.
